# Distinct sources of decision-related signals in visual cortex are represented in different local field potential bands

**DOI:** 10.1371/journal.pbio.3003873

**Published:** 2026-06-22

**Authors:** Yueyue Sapphire Hou, Pooya Laamerad, Liu D. Liu, Christopher C. Pack

**Affiliations:** 1 Department of Neurology and Neurosurgery, Montreal Neurological Institute, McGill University, Montreal, Quebec, Canada; 2 Department of Neuroscience, University of Pennsylvania, Philadelphia, Pennsylvania, United States of America; University of Glasgow, UNITED KINGDOM OF GREAT BRITAIN AND NORTHERN IRELAND

## Abstract

Fluctuations in single-neuron activity in the sensory cortex often correlate with perceptual decisions. This kind of correlation is often hypothesized to reflect a causal influence of sensory signals on decisions, but it can be attributed to various noncausal factors as well. To disentangle these different possibilities, we have examined local field potentials (LFPs) recorded from the middle temporal (MT) area and area V4 of nonhuman primates (*Macaca mulatta*) while they performed two different perceptual decision-making tasks. Compared to single-neuron spiking, LFPs have the advantage of being decomposable into frequency bands that are associated with different anatomical sources of input. More importantly, they persist when spiking activity is inactivated, which precludes a causal influence of the corresponding neural activity on behavior. We found that high-gamma frequency (70–150 Hz) LFP power was correlated with perceptual decisions and that this correlation disappeared when spikes were inactivated, consistent with a causal role for this frequency band in decision-making. These signals overlapped in time with decision signals in the lower gamma band (30–70 Hz), which persisted after spiking inactivation, suggesting a noncausal input. Interestingly, lower-frequency LFP signals (5–30 Hz) reflected both impending perceptual decisions and the outcome of preceding trials, suggesting a modulatory influence of recent experience on neural dynamics. Our results, therefore, reveal that neural activity multiplexes different sources of information about perceptual decisions and that these types of information can be estimated reliably from different LFP frequencies.

## Abstract 2

Neuronal activity in sensory cortex correlates with perceptual decisions, but whether these correlations reflect causal influences or other factors is unclear. This study shows that LFP frequency bands dissociate causal and non-causal signals, with high-gamma activity reflecting causal contributions and lower frequencies indexing contextual influences.

## Introduction

Many previous studies have characterized the relationship between spiking activity and perceptual decisions [1–4]. In these studies, the degree to which single-neuron activity predicts a subject’s decision is often quantified with a metric called choice probability (CP). Despite its widespread use, the interpretation of CP is still debated [5]. CP was originally assumed to represent a feedforward, or causal, influence of neural activity on decisions [1], while subsequent work favored a feedback influence of decision-making processes on neural activity [6]. A third alternative is that another factor, such as reward [7] or motor planning [8], influences both neural activity and decision-making.

Under reasonable assumptions [9–11], one can infer the importance of these different influences by examining the dynamics of CP over time [6,12,13]. For instance, feedback is often assumed to be slower than feedforward processing, so that it is more prominent during the later phases of the neuronal response to a stimulus. However, this is not always the case [14], and spike timing can be affected by many factors that are not necessarily related to decisions [14,15]. Given the correlational nature of CP, disentangling the various contributions to decision-related signals remains a significant challenge in the field.

As an alternative to single neurons, one can examine decision-related signals in local field potentials (LFPs), which reflect the coordinated activity of neural populations [16,17]. LFPs are characterized in terms of oscillations, and there is evidence that higher-frequency LFP oscillations are predominantly linked to feedforward sensory encoding [18–22], while lower-frequency LFPs primarily represent feedback processing [18,21–23]. Thus, examining CP in LFPs across specific frequency bands could provide information about the composite nature of CP signals.

To investigate this possibility, we recorded spikes and LFPs from two visual cortex structures (the middle temporal (MT) area and area V4) in four macaque monkeys performing either a random dot motion discrimination task or a match-to-sample form discrimination task. This allowed us to compute CP in different LFP frequency bands and to provisionally estimate the properties of causal and noncausal contributions to CP. To test these estimates more directly, we temporarily silenced local spiking activity pharmacologically, thereby eliminating the possibility of a causal influence of local neural activity on perceptual decision-making.

We found that significant CP signals were present in the high-gamma frequency range (70–150 Hz), suggesting a feedforward influence. Consistent with this idea, inactivation of spiking activity abolished CP in this frequency band. In contrast, CP in the low-gamma frequency band (30–70 Hz) persisted despite inactivation of spiking activity, suggesting that these CP signals were not causal in nature. CP detected in alpha and beta frequency bands (5–30 Hz) appeared to reflect a third type of influence, which was largely attributable to the outcome of previous trials. Our findings thus demonstrate that CP reflects multiple decision-related signals, which can be effectively distinguished through LFPs in different frequency bands.

## Results

The goal of this study was to disentangle the different hypothesized roles of decision-related neural signals in visual cortex. To do so, we calculated CP, using LFPs recorded from four rhesus macaque monkeys performing perceptual decision-making tasks. We then compared CP before and after local inactivation of spiking activity, to distinguish between causal and noncausal components of decision-related signals.

### Behavioral responses before and after inactivation

**Fig 1:**
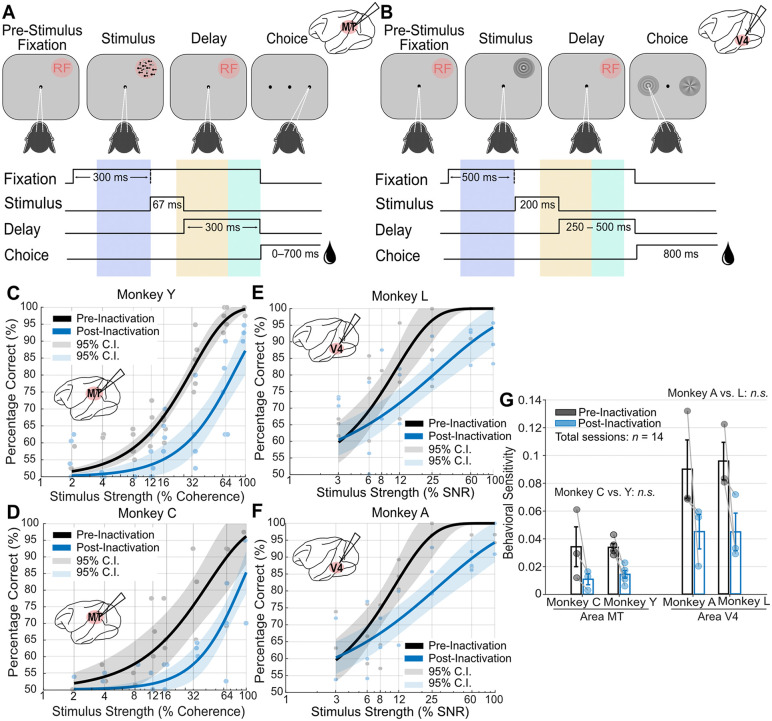
Behavioral performance before and after cortical inactivation in MT and V4. **(A)** Two rhesus macaques performed a random dot motion discrimination task. The stimulus was positioned in the receptive field (RF, pink circle) of recorded MT neurons. Animals reported the perceived motion direction by making a saccade to one of two choice targets. Task difficulty was varied by adjusting motion coherence, defined as the percentage of dots moving coherently in one direction. **(B)** Two other macaques performed a form discrimination task using circular or radial gratings placed in the RF of recorded V4 neurons. Animals reported which of the two targets matched the stimulus. Task difficulty was varied by adjusting the signal-to-noise ratio (SNR) of the form stimulus. **(A–B)** Pink elements and arrows are for illustration only and were not shown to the animals. The colored shades mark the epochs used to compute choice probability (CP) (blue: baseline; yellow: stimulus-response; green: delay). This period excludes the first 100 ms of fixation and the final 100–400 ms preceding the Choice phase, thereby isolating stimulus-driven neural activity without contamination from sensorimotor signals. **(C–F)** Psychometric curves for individual monkeys before (black) and after (blue) muscimol inactivation of local spiking activity pooled across experimental sessions: **(C)** Monkey Y and **(D)** Monkey C from the MT task; **(E)** Monkey L and **(F)** Monkey A from the V4 task. Curves show model-based Weibull fits with 95% confidence intervals. Small semi-transparent dots indicate correct percentages computed separately for each session at each stimulus level. Inactivation of MT or V4 caused a rightward shift in the psychometric functions, reflecting reduced sensitivity and confirming the causal contribution of these areas to their respective tasks. **(G)** Behavioral sensitivity (1/threshold) for each session and monkey. The underlying numerical data are provided at https://doi.org/10.5281/zenodo.20583873. Gray lines link paired sessions recorded on the same day. Each semi-transparent dot represents the sensitivity estimate obtained from a single session-level psychometric fit. Bars represent mean ± standard error of the mean (SEM) across sessions. “n.s.” denotes no significant differences that were observed between animals within either area MT or V4, indicating comparable behavioral performance across subjects.

We recorded neural activity from cortical area MT in two macaque monkeys that were trained to perform a motion discrimination task (Fig 1A) with random dot kinematograms (RDKs) [4,24]. In separate experiments, we recorded from area V4 [25] in two other monkeys trained to perform a form discrimination task (Fig 1B). These tasks were chosen to best match the functional selectivity of the recorded areas.

During each trial of both tasks (Fig 1A and 1B), the monkeys fixated a central point on the screen while a motion or form stimulus appeared within the receptive fields (RFs) of concurrently recorded neurons. Following a fixed delay, they reported their perceptual decision by making a saccade to one of two choice targets. In the MT task, these targets were positioned along the motion axis, and the monkeys were rewarded for choosing the direction of the RDK’s coherent motion. In the V4 task, one of the two targets matched the presented form, and monkeys were rewarded for selecting the matching target. Task difficulty was titrated on each trial by randomly varying the signal-to-noise (SNR) ratio of each stimulus [4,25].

To assess the causal contributions of these areas to perception, we pharmacologically inactivated the corresponding cortical site using muscimol, a GABA_A_ receptor agonist that silences local spiking activity [24,26]. Crucially, all included sessions (*N* = 14) contained both pre- and post-inactivation blocks recorded on the same day, ensuring a concurrent measure of neural and behavioral changes. Figure 1C–1F shows the behavioral results for each animal before and after inactivation pooled across all experimental sessions. As expected, inactivation consistently shifted psychometric functions to the right, indicating reduced performance and confirming a causal role of these areas in their respective tasks. For the most difficult stimulus condition (2% motion coherence for MT and 3% SNR for V4), performance approached chance levels (55%–60% correct).

For each task, we quantified session-wise behavioral performance by fitting the Weibull function to generate psychometric curves (see *Methods* for details). Figure 1G shows the sensitivity values for each monkey before and after inactivation, illustrating the consistency of behavioral performance across animals performing the same task. Specifically, sensitivity did not differ significantly between monkeys in either area MT (monkey C: 0.034 ± 0.014 versus monkey Y: 0.034 ± 0.003; *p* = 0.492, Wilcoxon rank-sum (WRS) test) or area V4 (monkey A: 0.090 ± 0.021 versus monkey L: 0.096 ± 0.014; *p* = 0.589, WRS).

### CP as a function of LFP frequency and behavioral epoch

**Fig 2:**
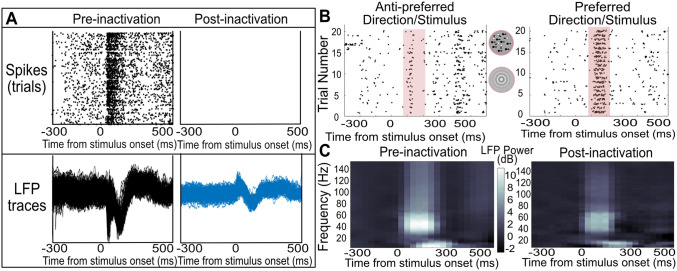
Measuring and computing LFP-based choice probability (CP). **(A)** A 16-channel linear electrode array was used to simultaneously record multi-unit spiking activity and local field potentials (LFPs) from the same cortical site. Within each session, the GABA_A_ receptor agonist muscimol was injected locally to silence spiking activity while preserving LFP signals. Example single-unit spike rasters across task trials and LFP traces from area MT illustrate the loss of spiking activity and persistence of LFP responses after inactivation. **(B)** The preferred and anti-preferred stimulus conditions for each recording site were determined from multi-unit spike counts during the stimulus-response period prior to muscimol injection (pink shades). The stimulus was either the neuron’s preferred or anti-preferred motion direction (MT) or form (V4). **(C)** Example time-frequency representations of LFP power before and after inactivation in area MT. Colors indicate power (in dB) normalized to the pre-stimulus baseline across frequencies from 0 to 150 Hz. LFP power decreased overall after inactivation, with a consistent change across frequencies.

For both experiments, we defined the preferred stimulus for each recording site based on multi-unit spike counts (Fig 2B), which exhibit greater selectivity than LFP signals [17,27] (see *Methods* for details). We then computed CP values from the distribution of LFP power across trials [16,17]. CP values above 0.5 indicate higher LFP power on trials in which behavioral choices aligned with the recorded neurons’ preferred stimulus; values below 0.5 indicate an inverse relationship between LFP power and preferred stimulus choices. Due to the nonnormal distribution of LFP power, we computed CP in the LFPs using robust scaling—normalization based on median and interquartile range—rather than the pooled z-scoring method that is commonly used for spike data. We validated this approach through simulations (see *Methods* and *Supplementary Information*).

In each session, we computed LFP-based CP before and during muscimol inactivation of spiking activity. Although muscimol consistently reduced spiking rates to zero [24], LFP activity persisted (Fig 2A), suggesting that it contained information about input to the MT and V4 recording sites [27]. A polarity analysis further shows that the evoked peak sign reversal in the lower panel of Fig 2A only occurred in a subset of channels, whereas response magnitude was consistently reduced (S6 Fig). Indeed, following muscimol inactivation, LFP power in response to the visual stimuli decreased by an average of 76.0%, with no consistent difference across frequencies (Fig 2C, Spearman’s ρ = 0.067, permutation test, *p* = 0.420).

Because neural-behavioral correlations have been shown sensitive to task difficulty [28,29], we equated behavioral performance between pre- and post-inactivation sessions by matching stimulus difficulty levels (see *Methods* for details). We then computed LFP-based CP across three behavioral epochs: the baseline period (−200–0 ms, pre-stimulus fixation), the stimulus-response period (50–250 ms after stimulus onset), and the delay period (250–400 ms, prior to the presentation of two choices). Among these, the stimulus-response epoch is the most informative for interpreting CP, as this is when sensory evidence first reaches the recorded cortical sites and before the motor response is initiated. We considered CP in three canonical LFP frequency bands commonly reported in prior literature: alpha-beta [5–30 Hz 17,30,31], low-gamma [30–70 Hz 32–34], and high-gamma [70–150 Hz 30,35]). Analysis of the stimulus-evoked LFP power spectra confirmed a largely broadband spectral structure before and after inactivation, with no change in narrowband oscillatory peaks (S7 Fig).

### High-gamma CP is consistent with a feedforward influence

**Fig 3:**
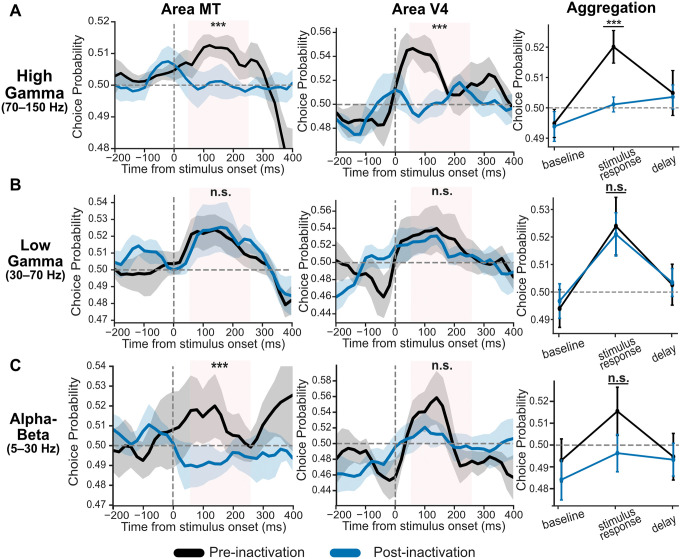
Frequency- and epoch-specific choice probability (CP) before and after cortical inactivation. **(A–C)** LFP-based CP traces (mean ± SEM) are shown for each frequency band across time from stimulus onset. CP was computed in 200 ms sliding windows advanced in a 20 ms step size. Each row represents one frequency band: (A) high-gamma (70–150 Hz), (B) low-gamma (30–70 Hz), and (C) alpha-beta (5–30 Hz). Within each row, panels display area-wise averages for area MT (left) and area V4 (middle), followed by the aggregated data across all four monkeys (right). Black and blue traces denote pre- and post-inactivation sessions, respectively. The pink shaded region indicates the stimulus-response epoch (50–250 ms after stimulus onset), during which statistical comparisons were conducted. Significance markers reflect results from Wilcoxon rank-sum (WRS) tests comparing pre- and post-inactivation CP values within this epoch (*p* < 0.05, * *p* < 0.01, ** *p* < 0.001, ***, “n.s.” denotes nonsignificant difference). Shaded regions in the left and middle panels indicate the SEM of time-resolved CP across sessions at each time point; smaller SEM values reported in the right panel and text reflect epoch-averaged CP collapsed across time. In the aggregated data, during the stimulus-response epoch, **(A)** high-gamma CP before inactivation was again significantly above chance (CP = 0.521 ± 0.005, *p* < 0.001, WSR test) but decreased to chance levels after inactivation (CP = 0.501 ± 0.001, *p* = 0.237, WSR test), with a significant reduction between drug conditions (*p* < 0.001, WRS test). **(B)** Low-gamma CP before inactivation was 0.526 ± 0.009 (*p* < 0.001, WSR) and 0.521 ± 0.006 (*p* < 0.001, WSR) after inactivation, with no reliable difference between drug conditions (*p* = 0.622, WRS). **(C)** Alpha-beta CP showed heterogeneous effects across areas and individuals. The underlying numerical data are provided at https://doi.org/10.5281/zenodo.20583873.

For high-gamma oscillations (70–150 Hz), CP peaked in the stimulus-response epoch, and the average CP value was significantly above chance before inactivation in both areas MT and V4 (Fig 3A). In MT, the mean CP was 0.509 ± 0.001 (mean CP ± SEM, *p* < 0.001, two-sided Wilcoxon signed-rank (WSR) test against 0.5), and in V4, 0.532 ± 0.006 (*p* < 0.001, WSR test). Thus, consistent with previous studies of spiking activity [4,36], there was a small but consistent relationship between trial-to-trial LFP fluctuations and perceptual decisions.

With inactivation and at an equivalent behavioral performance level, average CP values decreased significantly following inactivation (two-sided WRS test to compare before and after inactivation; area MT: *p* < 0.001; area V4: *p* < 0.001) and were no longer reliably different from chance, despite a marginal deviation in V4 (area MT: 0.499 ± 0.002, *p* = 0.342; area V4: 0.503 ± 0.001, *p* = 0.032; WSR test), which did not survive correction for multiple comparisons and was negligible in magnitude. These findings were consistent across individual monkeys (S1 Fig). In contrast, inactivation had no effect on CP measured during the baseline (*p* = 1.000, WRS) or the delay (*p* = 0.597, WRS) epochs (Fig 3A). These results were further confirmed by a linear mixed-effects ANOVA (S1Table), in which the epoch x inactivation interaction was marginally significant in the high-gamma band (*p* = 0.049) with no other significant main or interaction effects (all other *p* > 0.05).

In comparison to the decrease in CP, inactivation had a smaller effect on stimulus coding. A linear decoder trained on to classify stimulus identity from the population of high-gamma LFP signals (see *Methods*) maintained comparable accuracy before and after inactivation in MT (pre: 0.566 ± 0.029; post; 0.519 ± 0.027; *p* = 0.156, session-wise paired WSR test; S5A Fig) and V4 (pre: 0.626 ± 0.053; post: 0.616 ± 0.039; *p* = 1.000, paired WSR test; S5B Fig). Thus, the disruption of high-gamma CP with muscimol inactivation was likely not entirely due to a loss of stimulus encoding, but rather to a block in the transmission of sensory signals from visual cortex.

These results indicate that decision-related high-gamma activity depends critically on local spiking output [22,37]. When spiking was silenced, the corresponding CP signal disappeared, consistent with the causal hypothesis that high-gamma LFP power reflects sensory signals contributing directly to perceptual decisions. This interpretation aligns with prior findings that high-gamma oscillations closely track local spiking rates and the feedforward propagation of sensory information to downstream cortical areas [21,31].

### Low-gamma CP reflects correlated decision information

Low-gamma oscillations (30–70 Hz) also carried decision-related signals during the stimulus-response epoch (Fig 3B). Before inactivation, mean CP values were significantly above chance in both areas (MT: 0.517 ± 0.009, *p* < 0.001; V4: 0.535 ± 0.019, *p* < 0.001, WSR). Critically, these decision-related signals persisted after muscimol inactivation. Post-inactivation CP values remained significantly above chance (MT: 0.518 ± 0.011, *p* < 0.001; V4: 0.524 ± 0.009, *p* < 0.001, WSR), and no significant differences were observed between pre- and post-inactivation sessions (MT: *p* = 0.429; V4: *p* = 0.437; WRS test). This pattern was consistent across monkeys (S2 Fig), with all individuals showing low-gamma CP values that did not differ significantly before and after inactivation (Monkey Y, *p* = 0.984; Monkey C, *p* = 0.950; Monkey L, *p* = 0.192; Monkey A, *p* = 0.384; WRS). As with the high-gamma band, low-gamma CP values were at chance levels during the other trial epochs, with no change after inactivation (Fig 3B; baseline, pre-inactivation CP = 0.499 ± 0.010, *p* = 0.922, post-inactivation CP = 0.494 ± 0.007, *p* = 0.432; delay, pre CP = 0.507 ± 0.008, *p* = 0.275, post CP = 0.503 ± 0.007, *p* = 0.846, WSR). A mixed-effects ANOVA (S2 Table) confirmed these findings, showing a significant main effect of epoch (*p* < 0.05), but no inactivation or interaction effects (all other *p* > 0.05). Thus, despite the elimination of local spiking, the continued presence of low-gamma decision signals strongly suggests a component of CP that is not causally connected to behavior [38].

### Spectral weighting on decomposition of LFP population signals

Previous work has suggested that, at the population-level, some decision-related signals are distinct from those that encode the relevant sensory stimuli [39]. This suggests a parcellation of population activity into stimulus-related and decision-related activity. Given the different roles for decision signals described in the previous sections, we wondered whether a similar distinction would be found in a population analysis of LFP signals.

**Fig 4:**
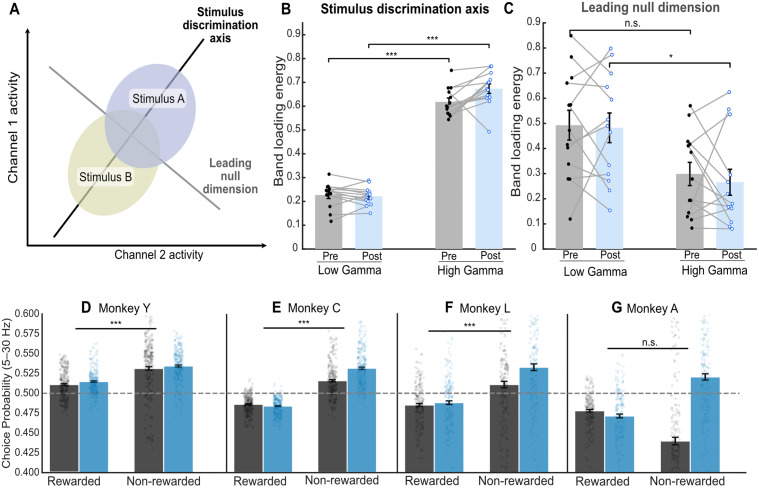
Frequency-specific decomposition of decision-related LFP activity. **(A)** Schematic illustration of the population activity space. **(B)** Frequency band loading energy for the stimulus axis, shown separately for low-gamma and high-gamma before (gray) and after (blue) inactivation. **(C)** Frequency band loading energy for the leading null dimension, shown separately for low-gamma and high-gamma before (gray) and after (blue) inactivation. (B-C) Bars indicate mean ± SEM across sessions, each dot represents one session, filled circles indicate before inactivation, open circles indicate after inactivation, and gray lines connect matched sessions. **(D–G)** Baseline (−300 to 0 ms) CP in the alpha-beta range (5–30 Hz) for trials preceded by rewarded and nonrewarded outcomes. (D) Monkey Y and (E) Monkey C, recorded in area MT. (F) Monkey L and (G) Monkey A, recorded in area V4. Black and blue bars indicate before and after inactivation sessions, respectively. Semi-transparent dots overlaid on each bar represent individual session-level CP values computed within the specified time-frequency window (−300 to 0 ms relative to stimulus onset, 5-30 Hz). The underlying numerical data are provided at https://doi.org/10.5281/zenodo.20583873. Statistical significance markers reflect WRS tests comparing rewarded and nonrewarded trials aggregated across pre- and post-inactivation conditions within this epoch (*p* < 0.05, * *p* < 0.01, ** *p* < 0.001, ***); n.s. denotes nonsignificant differences between reward conditions. Error bars represent the SEM across sessions.

For each session, we trained a linear classifier to discriminate stimulus identity, using the full LFP power spectrum (see *Methods* for details). The weight vector recovered by the decoder was taken as the stimulus axis. We then projected this axis out of the population activity and applied principal component analysis (PCA) to the residual matrix. The first principal component was taken as the leading null dimension orthogonal to the decoder-defined stimulus axis [39,40]. We compared the spectral composition of the stimulus axis with that of the null dimension in the same population activity space (Fig 4A).

Across areas and subjects, the stimulus axis was consistently high-gamma weighted (Fig 4B). High-gamma loading energy on that axis was 0.618±0.016 before inactivation and 0.674±0.020 after inactivation, whereas low-gamma loading was substantially smaller (before: 0.227±0.015, after: 0.222±0.011; one-sided WSR test, *p* < 0.001 in both conditions). By contrast, the null dimension showed a different spectral tendency (Fig 4C): low-gamma loading exceeded high-gamma loading both before and after inactivation (low- versus high-gamma; before: 0.493±0.059 versus 0.299±0.046 before inactivation; 0.482±0.059 versus 0.266±0.052 after inactivation), reaching significance after inactivation (post:  p<0.05, one-sided WSR test). This low-gamma weighting along the null dimension did not change significantly after inactivation (*p* = 0.787, two-sided WSR test).

Together, these results indicated that stimulus-aligned structure remained concentrated in high-gamma, whereas the persistent low-gamma signal was preferentially expressed in a dimension orthogonal to the stimulus axis. This pattern was consistent with prior work showing that the dominant activity patterns in a source population do not necessarily coincide with the most task-relevant subspace [41,42], and that choice-related signals can occupy dimensions misaligned with stimulus encoding [8,39].

### Alpha-beta CP and reward history

Decision-related signals in the alpha-beta band (5–30 Hz) were heterogeneous across monkeys, showing no consistent pattern within areas (S3 Fig). In area MT, monkey C exhibited persistent below-chance CP both before and after inactivation (before: CP = 0.494 ± 0.003, *p* < 0.001, WSR; after: CP = 0.492 ± 0.008, *p* < 0.001, WSR; *p* = 0.217, WRS), whereas monkey Y showed a strong decline from above chance CP before inactivation (CP = 0.521 ± 0.018, *p* < 0.001, WSR) to below-chance CP after inactivation (CP = 0.493 ± 0.011, *p* < 0.05, WSR); In area V4, monkey A maintained consistently positive CP values before and after inactivation (before: CP = 0.543 ± 0.034, *p* < 0.001, WSR; after: CP = 0.532 ± 0.015, *p* < 0.001, WSR; *p* = 0.197, WRS), while monkey L showed a reversal from CP at chance before inactivation (CP = 0.506 ± 0.025, *p* = 0.179, WSR) to significantly below-chance after inactivation (CP = 0.491 ± 0.021, *p* < 0.001, WSR). A mixed-effects ANOVA (S3 Table) showed no significant main or interaction effects (all *p* > 0.05), indicating that, even after accounting for individual differences across monkeys, alpha-beta CP still did not vary significantly with epoch or inactivation condition. Such inconsistency implies that this frequency band might integrate multiple influences, such as internal states or task strategies [21,31,43,44].

One possibility raised by previous studies is that CP on one trial could be influenced by the reward (or lack thereof) on the previous trial; this could affect arousal, which in turn could modulate both neural responses and behavioral performance [7,45,46]. In that case, CP during the baseline period in the alpha and beta bands might capture this covariation between neural and behavioral signals, without necessarily being related to the decision per se. To assess this possibility and to further disambiguate among individuals, we separated CP for trials that were preceded by a successful (rewarded) trial from those that had been preceded by an unsuccessful (nonrewarded) trial (see *Methods* for details).

Across monkeys, we found that CP in the alpha-beta frequency range was often higher following a nonrewarded trial than a rewarded one, a pattern observed in three of the four monkeys (Fig 4D–4F). When averaged across both pre- and post-inactivation sessions, mean CP for nonrewarded trials exceeded that for rewarded trials in Monkey Y (nonrewarded: 0.533 ± 0.001; rewarded: 0.513 ± 0.001; *p* < 0.001, WRS), Monkey L (nonrewarded: 0.522 ± 0.003; rewarded: 0.487 ± 0.001; *p* < 0.001, WRS), and Monkey C (nonrewarded: 0.524 ± 0.001; rewarded: 0.485 ± 0.000; *p* < 0.001, WRS), whereas Monkey A showed no significant difference (Fig 4G, nonrewarded: 0.480 ± 0.004; rewarded: 0.475 ± 0.001; *p* = 0.731, WRS). This reward-related component of CP persisted after muscimol inactivation. Averaging across post-inactivation sessions demonstrated a consistent pattern across all four monkeys: higher CP for nonrewarded than rewarded trials (nonrewarded versus rewarded; Monkey Y: 0.534 ± 0.001 versus 0.515 ± 0.001; Monkey C: 0.532 ± 0.001 versus 0.484 ± 0.001; Monkey L: 0.533 ± 0.004 versus 0.488 ± 0.002; Monkey A: 0.521 ± 0.004 versus 0.471 ± 0.002; all *p* < 0.001, WRS). Together, these results suggest that the alpha-beta decision signals carry a component reflecting prior reward history, which becomes more dominant when sensory-driven encoding is suppressed by inactivation of local spiking activity.

### CP latency across frequency bands

Intuitively, one might expect feedforward and feedback influences on decisions to appear at different time points in the neural response [6,47]. Indeed, feedforward and feedback inputs can occur with different latencies [48], though this is not always the case [14]. To address this issue, we quantified the latency of the emergence of CP in our data (see *Methods*). Before inactivation, the mean latencies were 100.7 ± 2.1 ms in high-gamma, 109.7 ± 3.6 ms in low-gamma, and 116.3 ± 4.1 ms in alpha-beta. However, paired comparisons across monkeys did not reveal reliable differences between bands (high-gamma versus low-gamma, *p* = 0.655; high-gamma versus alpha-beta, *p* = 0.179; low-gamma versus alpha-beta, *p* = 0.180, WSR tests). After inactivation, the mean change-point latencies were 112.1 ± 2.2 ms in high-gamma, 101.9 ± 3.0 ms in low-gamma, and 102.2 ± 4.4 ms in alpha-beta; again, none of the paired comparisons were statistically reliable (all *p* > 0.1). Thus, the relative temporal ordering across frequency bands was not significantly different across conditions.

Consistent with this result, the time of peak CP deviation was also similar across frequency bands. Mean peak times ranged from 144.2 to 154.1 ms before inactivation and from 138.0 to 150.1 ms after inactivation, and all paired comparisons were nonsignificant (all *p* > 0.1 before and after inactivation, WSR tests). Similarly, the magnitudes of peak CP were not statistically different across frequency bands before or after inactivation (all *p* > 0.1). Together, neither latency nor magnitude could reliably separate mixed sources of decision-related signals in our dataset.

## Discussion

Using LFP-based CP measurements and spiking inactivation in nonhuman primates performing visual decision tasks (Figs 1 and 2), we have identified distinct frequency-dependent features of decision-related neural activity. High-gamma frequencies appear to reflect feedforward decision signals, as they disappeared with muscimol inactivation (Figs 3A and 4B). In contrast, decision signals in the low-gamma frequency persisted with inactivation, indicating that this component of CP did not reflect a direct causal influence (Figs 3B and 4C). CP in alpha and beta bands revealed influences that likely reflect modulation based on reward history (Figs 3C and 4D,4G).

### Comparison with previous work

Previous work has used CP to investigate the neural bases of decision-making across various brain regions, including the parietal cortex [13,29,49] and early visual areas [43,50,51], using behavioral paradigms such as visual discrimination [3,4,52] and change-detection tasks [53,54]. Recent work in this area has hypothesized different time courses for feedforward and feedback influences on CP [6,11]. Feedforward components of CP are thought to arise early in the stimulus-response period, with feedback influences arising later [6,9,39,47]. These correlational studies are, however, limited by the fact that the relationship between the timing of signals and their anatomical source is not entirely straightforward in visual cortex [14]. Moreover, fluctuations in neural activity are not strictly attributable to task performance [8,24,49]. Thus, there is increasing emphasis on causal methods for probing the circuitry of decision-making [39,50].

For example, neurons in parietal cortex often exhibit strong choice-related signals, and reversible inactivation can lead to significant perceptual deficits [29]. But other studies have found little effect of parietal inactivation on behavioral performance, suggesting a noncausal component of these decision signals [13,49,55,56]. A similar pattern has emerged in area MT, with most studies finding large perceptual deficits with inactivation [13,49,55] and others finding little or none [24]. It is clear from these studies that the causal influence of a cortical area on perceptual decisions cannot be predicted from CP alone, but rather depends on the precise nature of the task, the training procedure, and the stimuli [8,24,29].

A few previous studies have attempted to evaluate CP based on LFP power [16,17]. In one study, the authors recorded spikes and LFPs from V1 and V4 while monkeys performed a two-alternative forced-choice orientation discrimination task [16]. Similar to our findings, they reported decision-related information in the alpha and beta frequencies, as well as in high-gamma frequencies during the stimulus-response period. Liu and Newsome recorded LFPs from area MT during a speed discrimination task and found significant LFP-based CP in frequencies above 40 Hz, while LFP power below 30 Hz actually exhibited below-chance CP values [17].

### Frequency-specific neural computations in decision-making

The strong reduction of high-gamma CP signals following muscimol inactivation (Fig 3A) supports the hypothesis that these signals represent feedforward processing [20,21,57]. This might result from the fact that higher-frequency LFPs are generally found in the upper layers of cortex, which send feedforward projections to higher-level areas [30], although we were unable to verify the laminar locations of our recordings. We attempted to examine the laminar dependency of CP signals in our data, but we found no reliable depth gradients or superficial-deep contrasts in any frequency band before and after inactivation (see *Methods* for details), presumably because we did not have a reliable way to estimate the laminar position of our recording channels [58].

The persistence of low-gamma CP signals (Fig 3B) after muscimol inactivation could reflect feedback signals from decision-related areas [6,9,47]. Previous studies found that low-frequency LFP components in V4 could remain intact when local spiking was strongly reduced, which suggests that such signals primarily reflected long-range synaptic input rather than local output [38,59]. Alternatively, they could reflect shared noise correlations between neural activity in MT (or V4) and other areas, such as V3A [60], MST [61], or IT [25]. These could be feedforward in nature, in the sense that their sensory activity might inform decisions, rather than the other way around. We have taken a simplified view of feedforward and feedback contributions here, but feedback needs not be noncausal; in recurrent decision circuits it can help drive commitment itself, for example, by accelerating ramping toward the bound [62], and the very short trials in our task likely limited such effects.

CP signals in the alpha and beta bands were also found before and after inactivation (Fig 3C). Given that the results were not consistent across monkeys, this alpha-beta component of CP can hardly be attributed to any mechanisms related to sensory processing. Rather, they seem to reflect the reward outcome of previous trials, as has been found for spiking activity [7]. One interpretation of this finding is that absence of reward transiently boosts arousal or attention, which influences both stimulus coding and task performance [7,44]. We could not test this possibility directly without quantifying arousal by pupil size, but we took an indirect approach assuming that arousal would affect broadband, epoch-invariant modulation [63,64]. However, we still found no evidence to favor the interpretation that arousal mediated the relationship of alpha-beta CP and reward history. Alternatively, such reward history-dependent modulation may reflect changes in internal state, which would be better addressed in paradigms designed to study learning.

### Interpretation of CP values

Although the absolute magnitude of our LFP-based CP values was typically near 0.5, such values are consistent with the literature on decision-related activity in sensory cortex [65]. Early work in area MT demonstrated that perceptual decisions could be accounted for by the activity of a relatively small number of neurons [36]. Subsequent analyses showed that CPs below 0.6 are pervasive because only a small fraction of neurons need to be activated to yield decisions [65]. From this perspective, the modest but statistically significant deviations from 0.5 observed in CP values reflected the relationship between population-level neural activity and perceptual decision.

On average, our MT results exhibited relatively lower CP magnitudes than our V4 dataset. This difference likely stemmed from the temporal structure of our behavioral paradigms. In the MT task, the brief presentation of a visual stimulus in our experiments might not have exerted a sufficiently strong influence on local neural activity, whereas the two-fold longer presentation of a visual stimulus in our V4 experiments might have allowed more time to integrate sensory information.

## Conclusions

Much previous work has been concerned with the question of whether decision-related signals in visual cortex are causal in nature. By analyzing LFP signals in different frequency bands, we detected both causal and noncausal sources of decision-related activity in visual cortex, with largely overlapping time courses. We also detected a third source related to the behavioral outcome of previous trials. The relative balance of these signals likely depends on the structure of the behavioral task and the strategies used by the subjects. Given this complexity, our results suggest that LFPs can be useful in revealing information about perceptual decision-making that is not readily apparent in spiking activity.

## Materials and methods

The experimental methods (i.e., behavioral training and adaptation) were detailed in previous papers [8,24,66]; this paper primarily involves a re-analysis of the associated data. Only the relevant information is reiterated here.

### Ethics statement

All procedures adhered to the regulations established by the Canadian Council on Animal Care and were approved by the Montreal Neurological Institute’s Animal Care Committee (protocol no. 5031). Daily operations were supervised by the institute’s veterinarians and trained animal health staff.

### Observers

Four adult female rhesus macaque monkeys (*Macaca mulatta*) (Monkey Y, age: 10 years; Monkey C, age: 8 years; Monkey L, age: 10 years; Monkey A, age: 8 years; all weighted 5–7 kg) participated in this study. The animals were housed in a temperature- and pressure-controlled socially accessible environment under a 12-hour light/dark cycle. Behavioral training and experimental recordings were conducted during the light phase of the cycle. Animals sat comfortably while head-fixed in a custom-designed primate chair. Identical stimuli, timing, and rewards were used for both monkeys of the same task. A solenoid-operated reward system was used to dispense juice reward to the monkeys (Crist Instruments Co.).

### Experimental setup

Prior to the experiments, an MRI-compatible titanium head post (Hybex Innovations, Montreal) and a plastic recording cylinder were affixed to each monkey’s skull under general anesthesia. Areas MT and V4 were identified based on transitions from white matter to gray matter, as well as the electrode depth, the prevalence of robust, direction-selective visual responses, and the relationship between RF size and eccentricity. More details could be found in the respective published work [24,67]. Eye movements were monitored with an infrared eye tracking system (EyeLink1000, SR Research) with a sampling rate of 1,000 Hz.

### Neural recording

Single units were recorded utilizing linear microelectrode arrays (V-Probe, Plexon) comprising 16 contacts. Initially, the electrode array was lowered to the designated depth, followed by the estimation of multi-channel RFs, through the manual positioning of a moving bar within the visual field in area MT, and through the manual presentation of sparse noise stimuli at various spatial locations within the visual field in area V4. The RF mapping was then done on each animal by fitting with a 2D Gaussian function to recover the RF centers and sizes [24,67].

For area MT, neuronal signals were continuously monitored during acquisition via computer display. Spike signals were thresholded in real-time, with local field potential (LFP) signals undergoing band-pass filtering at 0.5–150 Hz, while spike signals were band-pass filtered at 150–8 kHz, with monitoring conducted on an oscilloscope and loudspeaker. Spike signals were thresholded in real-time, and spikes were assigned to single units by a template-matching algorithm (Plexon MAP System). Then, spikes were manually sorted offline using a combination of automated template-matching, visual inspection of waveforms, clustering in the space defined by the principal components, and absolute refractory period (1 ms) violations (Plexon Offline Sorter).

For area V4, neuronal signals were recorded using an Intan Technologies system and filtered between 0.5 and 7 kHz. Real-time spike signals were thresholded by exceeding ±3 standard deviations, estimated for each channel. Segments surrounding each threshold crossing were extracted and clustered using UltraMegaSort 2000, a *k*-means-based clustering algorithm [68].

### Visual stimuli and behavioral paradigm

#### Random dots motion discrimination task in area MT.

Visual motion stimuli were presented at a frequency of 60 Hz with a resolution of 1,280 by 800 pixels. The viewing area covered 60° by 40° at a distance of 50 cm. The monkeys were trained to perform a motion discrimination task, with RDK. During each session, neuronal direction and speed preferences were measured using 100% coherent dot patches positioned within the RFs found at the recording site on that day. Specifically, the RF locations were quantified by fitting a 2D spatial Gaussian to the neuronal response measured across a 5 x 5 grid of stimulus positions in an offline analysis. The grid comprised of moving dot patches centered on the initially hand-mapped RF locations. We confirmed that all neurons included in our analysis had RF centers within the stimulus patch used for the behavioral experiments. The speed of the dots was chosen to match the preference of the MT neurons in proximity to the recording site. The direction of motion was consistently selected based on the preferred or anti-preferred direction of the neurons being examined. The stimulus size also matched the RF size of the MT neurons (mean radius = 6.3° ± 1.2°). The RDK coherence was randomly selected on each trial from nine values that encompassed the range of the monkey’s psychophysical threshold (0%, 2%, 4%, 8%, 12%, 16%, 32%, 64%, and 100%).

The individual trial structure of a random dot motion discrimination task is detailed below (also see Fig 1A). On each trial, animals established and maintained fixation for 300 ms, followed by a brief presentation of the dot coherence patch (typically for 67 ms) on the RF centers. The monkeys were then required to maintain fixation for another 300 ms, after which the fixation point disappeared, two choice targets appeared, and the monkey made a saccade to the corresponding target to indicate its perceived motion direction (preferred or anti-preferred relative to the isolated neurons) in exchange for juice rewards. Targets were typically positioned at 10° eccentricity, with each pair of angles sampled at 45° intervals on the screen. For example, if a neuron’s preferred direction was 45°, the targets were intentionally positioned to correspond with both the preferred and anti-preferred directions, meaning they would be placed at 45° and 225°, respectively. The distance between the two saccade targets was typically 20°. The appropriate saccade direction correlated with the most similar saccade target direction (i.e., the monkey performed the rightward saccade to correctly report rightward motion). The monkey was required to indicate its decision within 700 ms after the onset of the choice targets. In trials with no motion signal (0% coherence), rewards were randomly assigned in half of the instances. If fixation was disrupted at any time during the stimulus, the trial was aborted. In a typical experimental session, the monkeys performed the task on 20–40 repetitions of each stimulus.

#### Match-to-sample form discrimination task in area V4.

Visual stimuli were back-projected onto a semi-transparent screen using an LED video projector (VPixx Technologies, PROPixx) with a refresh rate of 120 Hz. The screen spanned an area of 80° by 50° at a distance of 81 cm. A neutral gray color (54 cd/m^2^) was used as the background for the task. The monkeys were trained to perform a form discrimination task, with circular and radial gratings. During each session, neuronal size and position preferences were measured using 100% contrast of circular and radial grating positioned within the RFs found at the recording site on that day.

The individual trial structure of a match-to-sample form discrimination task is detailed below (also see Fig 1B). Monkeys were trained to identify which of the two choice stimuli (a circular and a radial grating) matched a previously presented sample stimulus, which can be either a circular or radial grating. On each trial, an initial 500 ms fixation was followed by the presentation of a sample stimulus for 200 ms. This sample stimulus had Gaussian noise added at one of eight levels to encompass the animal’s psychophysical threshold range in terms of signal-to-noise (SNR) ratio (0%, 3%, 6%, 8%, 12%, 25%, 60%, and 100%). 0% SNR indicates pure noise, whereas 100% SNR is a noiseless stimulus. After the sample stimulus was removed, a randomized 250–500 ms delay period followed. Then, both noiseless (100% SNR) circular and radial grating stimuli appeared on either side of the fixation point as the response cues. They were positioned ±7° from the fixation point along the horizontal meridian. Their locations were randomly shuffled from trial to trial to prevent the animals from developing a fixed sensorimotor mapping. The monkeys should make a saccade towards either of the two cues that matched the sample stimulus and maintain fixation on it for 800 ms to exchange for a liquid reward. If the choice made was incorrect, a 1.50-s timeout followed the choosing period before the onset of the next trial. Note that few trials on 0% sample stimulus collected from animals on this task as they refused to do trials only containing pure noise. Thus, we combined 0% stimulus trials with other stimulus levels if needed for analyses.

### Infusion of muscimol

The linear V-Probe contained a glass capillary with an inner diameter of 40 μm. The capillary was positioned at one end between contacts 5 and 6 of the array, with contact 16 located at the most dorsal-posterior position. The outermost end was connected via polyethylene tubing (PE20, inner diameter = 0.38 mm) to a 10 μl micro-syringe (Hamilton) to ensure continuous and smooth injection.

Muscimol, a GABA_A_ agonist (Sigma), was dissolved in sterilized saline (pH ~= 7) to achieve a concentration of about 10 mg/mL [26]. In half of the sessions, muscimol (generally 2 μl at a rate of 0.05 μl/min) was administered via the fluid channel in the V-Probe to inhibit its adjacent neural activity. The spread of muscimol is typically less than 2 mm, so it is unlikely that the drug spread into nearby retinotopic regions of cortex [69].

Behavioral testing commenced 40–50 min post-infusion at each site. Muscimol infusion resulted in complete suppression of detectable spiking activity across all electrode contacts. The probes remained at the infusion depth within the cortex for the duration of the session, allowing us to verify that spiking activity remained silenced throughout the post-muscimol period, which typically lasted 2.5–3.5 hours. The inactivated area returned to its normal state typically within two days. Experiments involving muscimol were performed at a frequency not exceeding once per week.

As described previously [24,67], control saline injections did not alter spiking activity (S8 Fig) on any channel and had no effect on behavioral performance; there was no behavioral effect of injecting saline or of injecting muscimol at sites distant from the recording site, indicating that the muscimol results were due to local inactivation, rather than to any general effects of the injection.

### Analyses of neural and behavioral responses

Only recording sessions that contained pre- and post-muscimol data within the same recording session were included for analysis. We excluded trials in which monkeys failed to execute a saccade toward one of the choice targets or broke fixation during the stimulus presentation or delay periods. Overall, a total of 14 control sessions and 14 corresponding inactivation sessions were included for analyses (10 sessions for monkey Y, 6 sessions for monkey C, 6 sessions for monkey L, and 6 sessions for monkey A). On average, monkey Y completed 318 trials per session, and monkey C performed 358 trials per session, monkey L completed 1,626 trials per session, and monkey A performed 1,259 trials per session, encompassing both correct and error trials of the task. The comparable number of trials was performed after the infusion of muscimol (*p* = 0.992, Welch’s *t* test).

#### Psychometric curve fitting.

The monkeys’ behavioral performance as a function of dot coherence or form SNR level was characterized by fitting a Weibull model to the proportion of correct responses by using nonlinear regression (*fitnlm* in MATLAB). The two-parameter Weibull function is,


$$\begin{array}{c} \psi (x)=\;\text{1}\text{0}\text{0}\;\times \;(\text{1}-\text{0}.\text{5}\;e^{-{\left(\frac{x}{\alpha }\right)}^{\beta }}) \end{array}$$

where ψ(x) is the predicted proportion of correct responses at stimulus level x (motion coherence % for area MT and SNR % for area V4); α is the perceptual threshold, and β determines the slope of the function; and x is stimulus coherence or contrast in percentage. Under a conventional parameterization, α corresponds to x yielding ~81.6% correct performance. Because the fit was obtained in log-percentage space, trials with 0% stimulus strength were excluded to avoid singularities under the log transform. We plotted the resulting psychometric curves with 95% confidence intervals at each stimulus level using model-based prediction intervals. Pooled psychometric fits are shown in Fig 1C-1F, whereas Fig 1G shows statistical analyses conducted on session-wise fits. In Fig 1G, behavioral sensitivity was quantified as the inverse of the perceptual threshold at which behavioral performance reached 81.6% for each monkey in each recording session, such that larger values indicate better perceptual sensitivity.

#### LFP preprocessing and re-referencing.

LFPs from the 16th channel, located at the most dorsal-posterior position, were utilized as the reference signal, while the LFP signals from the other channels were re-referenced accordingly so that artifacts from eye blinks and other physiological factors were eliminated. The line noise at 60 Hz was removed in LFP raw signals. We utilized the Chronux toolbox [70] in MATLAB, applying its multi-taper method for Fourier power spectral estimation, using two tapers derived from discrete prolate Slepian sequences to achieve a frequency resolution of 1 Hz.

To compare LFP power before and after muscimol inactivation, we used a nonparametric statistical method. First, we calculated the log ratio of LFP power spectra (pre- versus post-inactivation) across trials from all sessions. The difference in total power before and after inactivation was quantified by computing the area under the power spectral density (PSD) curves (MATLAB’s *trapz* function). To assess whether this power difference varied significantly as a function of frequency, we computed Spearman’s rank correlation coefficient (ρ), testing statistical significance through a permutation test (*n* = 5,000 permutations).

#### Spectral parameterization of evoked LFPs.

To evaluate the spectral structure of the stimulus-evoked LFP prior to frequency band–based CP analyses, we computed PSD during the stimulus-response epoch (50–250 ms post-stimulus) at the trial level. Each trial’s spectrum was parameterized using the FOOOF algorithm [71] over a range of 5–150 Hz, to separate broadband aperiodic structure from oscillatory peaks. To ensure robust fitting across a wide range of spectral shapes, we evaluated multiple FOOOF model configurations that varied in peak width limits (1–12 Hz), maximum number of peaks (6–8), minimum peak height (0.03–0.05), and aperiodic mode (fixed or knee). For each trial, the best-fitting model was selected based on a composite score that favored higher explained variance (R²) and lower fitting error, with a small penalty applied to excessive numbers of detected peaks. For each accepted fit, we extracted aperiodic components and oscillatory peak parameters. Peak center frequencies were pooled across trials, channels, and sessions to generate descriptive histograms of oscillatory peak distributions before and after inactivation in S7 Fig.

#### LFP-based choice probabilities (CP).

The computation of LFP-based CP, a metric that quantifies trial-to-trial fluctuations between LFP power variability and psychophysical choices, was conducted, mostly using established methods developed for spiking activity [4].

We first subdivided trials into two choices defined by neurons’ stimulus selectivity and behavioral outcomes. We included sites for which the preferred stimulus had at least a 50% higher multi-unit spike count in the stimulus-response period (50–250 ms relative to stimulus onset) than the spikes in the baseline (−200–0 ms relative to stimulus onset). The stimulus-response interval was selected due to the significant correlation between the spikes observed during this time window and the animals’ behavioral choices [66]. Stimulus selectivity was assessed across multiple neurons at the highest dot coherence levels (64% and 100% for the MT task; 60% and 100% for the V4 task) for every electrode channel independently.

In this way, a preferred choice contained trials that exhibited either a preferred stimulus with a correct response or a nonpreferred stimulus with an incorrect response; an anti-preferred choice contained trials that involved either an anti-preferred stimulus with a correct response or a preferred stimulus with an incorrect response.

For the reward history analysis, current trials were additionally partitioned according to behavioral outcome of the immediately preceding trial. Specifically, for each valid trial n, we identified trial n−1 in the original behavioral dataset and used its outcome to classify the current trial as rewarded if the previous trial had been correct, or as nonrewarded if the previous trial had been incorrect. Importantly, the LFP power used for CP estimation was always taken from the current trial, not from the preceding trial. Within each reward history condition, the current trials were then subdivided into preferred choice and anti-preferred choice using the same behavior-choice rules described above.

Because CP is meant to be insensitive to the mean amplitude of neural responses, most analyses of CP first involve normalization of firing rate, using z-scoring or balanced z-scoring [72]. However, given the nonnormal distribution of LFP power, this approach has the potential to introduce biased estimate of CP. To assess this possibility, we used the NeuroDSP toolbox [73] to simulate LFP data from 100 trials of one-second duration (S4A Fig), each comprised of stochastic periodic and aperiodic components; these were generated using the function *sim_combined* with the default parameters. We implemented this procedure twice to simulate trials associated with two behavioral choices that differed in mean amplitude across frequencies, one being 100 times greater in amplitude than the other (S4A Fig). Then, we computed spectrograms to represent the simulated LFP data before normalization (S4B Fig). A proper normalization would yield CP values of 0.5, because CP computation takes into account the variation between choices, not the difference in mean. However, we found that z-scoring and balanced z-scoring failed to eliminate the mean difference between the signals associated with the two choices, largely distorting the signals that were associated with lower-frequency LFP power (S4C Fig). This distortion arises because nonGaussian distributions like LFP power are susceptible to the skewness of the PSD. Thus, z-scoring disproportionately emphasizes outliers when the distribution is nonnormal, biasing LFP-based CP estimation.

We therefore used a robust scaling method that avoids such distortions. Specifically, we first resampled trials with each of the two choices to ensure that the preferred and anti-preferred choices had equal number of trials for each stimulus condition. We then normalized separately for the preferred and anti-preferred choices. For this normalization, we used the median and interquartile range (IQR) of the distribution of LFP power for each choice:


$$\begin{array}{c} X_{normalized}=\;\frac{X-median}{IQR} \end{array}$$

where X is the original distribution and X_normalized_ is the normalized version of the original choice by its median and IQR. This approach does not rely on any specific distributional assumptions but rather quantifies the central tendency and dispersion to better resist outliers in LFP. In our simulated example, robust scaling aligned the two choices to a uniform scale with identical values, thereby recovering the accurate CP values across all frequencies (S4D Fig). This simple simulation confirmed that robust scaling effectively returned CPs to chance when the LFP mean power is the only difference between two simulated LFP profiles, demonstrating its suitability for normalizing LFP power.

Following appropriate normalization, the distributions of two normalized choices were subsequently analyzed using receiver operating characteristic (ROC) analysis to compute CP. CP was determined by calculating the area under the ROC curve. The area under the ROC curve ranges from 0.0 to 1.0, reflecting the performance of an ideal observer in determining motion direction based on the trial-to-trial LFP power. Values of 1.0 and 0.0 represent perfect classification, whereas a value of 0.5 signifies performance equivalent to random chance classification.

CP can be sensitive to overall behavioral performance, and the infusion of muscimol significantly impaired the monkeys’ performance. It was therefore necessary to equalize performance pre- and post-muscimol by selecting trials with different stimulus levels. In the MT task, for Monkey Y, we used coherence levels of 0%, 4%, and 8% before muscimol injection and 0%, 8%, 12%, and 16% after injection; for Monkey C, we used coherence levels of 0%, 4%, 8%, and 12% before muscimol injection and 0%, 8%, 12%, and 16% after injection. In the V4 task, for Monkey L, we used stimulus SNR levels of 0%, 6%, 8%, and 12% before muscimol injection and 0%, 8%, 12%, and 25% after injection; for Monkey A, we used stimulus SNR levels of 0%, 6%, 8%, and 12% before muscimol injection and 0%, 12%, 25%, and 60% after injection. These stimulus levels equated behavioral performance before and after inactivation, at ~65% correct across monkeys for analyses of area MT (Monkey Y, 66%; Monkey C, 62%); at approximately 78% correct across monkeys for analyses of area V4 (Monkey L, 80%; Monkey A, 75%). However, because differences in stimulus strength could bias CP, we included trials of 0% level in both pre- and post-inactivation conditions. These ambiguous trials, on which monkeys performed at chance, controlled for any confounds related to the stimulus.

We then estimated CPs based on LFP power. First, we calculated the CP for each frequency bin individually, utilizing power measurements obtained from a sliding window of 200 ms width, advancing in 20 ms increments. We calculated the averaged CPs across three distinct epochs relative to stimulus onset: baseline (−200–0 ms), which occurred during the pre-stimulus fixation, stimulus-response (50–250 ms), which spanned between stimulus onset and post-stimulus fixation, and delay (250–400 ms) spanning before the appearance of the choice targets. The stimulus-response epoch was defined by the time that LFP power response was maximal (Fig 2C), and to analyze CPs across the two areas, we kept the epoch length consistent. We statistically quantified CPs by averaging power across three frequency bands: alpha-beta (5–30 Hz), low-gamma (30–70 Hz), and high-gamma (70–150 Hz). We noted that spike waveforms have statistically little effect on any broadband LFP power [57].

#### CP latency.

We estimated CP latency using a threshold-free change-point analysis [74,75]. Specifically, we analyzed the CP trace from −200–250 ms relative to stimulus onset using a two-segment model. In this model, the CP trace was approximated by one mean level before a candidate transition time and a second mean level after that time. The transition time was constrained to occur within the post-stimulus interval (50–250 ms relative to stimulus onset). Before fitting, each trace was aligned to its dominant post-stimulus direction of deviation from 0.5 so that the main CP excursion was positive. We defined CP latency as the transition time that provided the best fit to the data, that is, the one that minimized the residual sum of squares. This procedure quantified the onset of a sustained shift in CP without relying on a fixed amplitude threshold. The latency analysis contained a 20 ms bin size, so each segment was required to include at least two samples (i.e., an effective minimum segment duration of 40 ms). We also measured the time of peak CP deviation within the same interval, defined as the time point at which the CP trace reached its maximum deviation from 0.5; the peak magnitude was defined as the corresponding maximum deviation from 0.5. Mean ± SEM summaries for latency, peak time, and peak magnitude were computed across sessions x frequency bins.

#### Depth dependence of CP.

To test whether CP varied in terms of spatial organization, we analyzed CP at different frequency bands as a function of relative channel position along the linear probe. Because probe angle varied across sessions and laminar boundaries were not identified, channel index was treated as a within-session measure of relative depth. For each session and frequency band, we quantified depth dependence in two ways: (1) a linear slope relating CP to relative channel depth, and (2) a superficial-deep contrast defined as the difference between mean CP in contacts closer to the probe tip versus contacts away from the tip. Channels that did not have sufficient balanced trial counts for preferred and anti-preferred choices to estimate CP were excluded. Statistical tests were performed at the session-level separately for each frequency band and condition.

#### Population decoding of stimulus information from LFP.

We trained a simple linear decoder on simultaneously recorded high-gamma LFP spectra to classify stimulus information. For each session, we focused on correct trials and restricted to the high-evidence stimulus levels (MT coherence: 32%, 64%, 100%; V4 SNR: 25%, 60%, 100%), where stimulus information is expected to be the strongest. LFP power was computed during the stimulus-response epoch (50–250 ms after stimulus onset), and features were restricted to the high-gamma band (70–150 Hz). We averaged log power within the high-gamma band for each simultaneously recorded channel, yielding one high-gamma feature per channel. Trials were balanced within each stimulus level by random downsampling. For each condition separately, a logistic regression classifier with L2 regularization (function *fitclinear*, MATLAB) was trained and evaluated using repeated stratified K-fold cross validation (5 folds, 20 repeats). Feature standardization was performed using training data only and applied to held-out test data within each condition. Decoder performance was quantified as held-out classification accuracy. For each session, the classification accuracy was first pooled across folds within each repeat and then averaged across repeats to obtain a session-level estimate. We trained decoders separately for pre- and post-inactivation data because this analysis was designed to quantify stimulus information that could be recovered from the population LFP signal within each condition. For this comparison between pre- and post-inactivation conditions, the total number of retained trials was matched across conditions within each session.

#### Decomposition of LFP population signals.

To compare stimulus-aligned and orthogonal structure in population LFP activity, we represented each session as a single-trial feature matrix $X\in R^{T\times p}$, where each row T corresponded to one trial and each column to one channel-by-frequency feature (p=C×F). Features were the log-transformed stimulus-epoch LFP power values (5–150 Hz) concatenated across simultaneously recorded channels. Using this matrix, we defined two distinct types of axes.

First, we defined a decoder-based stimulus readout axis by training a linear classifier to discriminate stimulus identity. The resulting weight vector w was normalized to unit length, $\hat{u}=w/∥w∥$, and taken as the stimulus axis, corresponding to the direction in population activity that best separated the two stimulus classes.

Second, to characterize activity structure not captured by that axis, we removed the projection of each trial onto the stimulus readout axis,


$$\begin{array}{c} X_{⊥}=X-(X\hat{u}){\hat{u}}^{⊤}, \end{array}$$

and applied PCA to the standardized residual matrix $X_{⊥}$. The first residual principal component was taken as the leading null dimension [39,40]. To verify the geometry of this decomposition, we quantified the relationship between the null dimension $v_{⊥,\text{1}}$ and the stimulus axis $\hat{u}$ using the absolute cosine similarity, $|{\hat{u}}^{⊤}v_{⊥,\text{1}}|$, and the corresponding angle, $\theta =\text{arccos}(|{\hat{u}}^{⊤}v_{⊥,\text{1}}|)$, in degrees. As expected, the null dimension was orthogonal to the stimulus axis across sessions, with cosine alignment values on the order of ${\text{10}}^{-\text{17}}$ and corresponding angles of ${\text{90}}^{\circ }$.

To assess the spectral contribution of each axis, we quantified loading energy as the fraction of squared component weight assigned to features within each frequency band. For axis k with weight vector $v_{k}$, loading energy in band b was defined as


$$\begin{array}{c} E_{k,b}=\frac{\sum_{j\in I_{b}}\overset{\text{2}}{\underset{k,j}{v}}}{\sum_{j=\text{1}}^{p}\overset{\text{2}}{\underset{k,j}{v}}}, \end{array}$$

where $I_{b}$ denotes the set of features belonging to band b. Because this metric depends on squared weights, it is invariant to the arbitrary sign of the component vector.

### Quantification and statistical analyses

We used nonparametric and ANOVA techniques for the statistical evaluation of our data. All statistical studies were performed using MATLAB’s and Python’s core tools and custom programs.

We employed the two-sided WSR test to assess the significance level of LFP-derived CPs relative to 0.5 when the sizes of the datasets were equal. The difference in LFP-derived CPs before and after muscimol infusion was evaluated using the WRS test. Significance was determined at *p* < 0.05.

To test whether the effect of inactivation interacted with behavioral epoch per frequency band, we fitted a linear mixed-effects model:

CP ~ epoch × condition + (1|monkey),

where CP was treated as a continuous dependent variable; epoch (baseline, stimulus, delay) and inactivation condition (pre, post) were fixed factors; and monkey was included as a random grouping factor to account for repeated measures across epochs. Coefficients represent changes in CP relative to the baseline epoch and post-inactivation condition, which together define the model’s reference level.

All analysis code, including the LFP-CP pipeline and validation simulations, is publicly available (see *Code and Data Availability*).

## Supporting information

S1 Table Mixed-effects ANOVA results for high-gamma (70–150 Hz) band. Results of a linear mixed-effects model testing the interaction between behavioral epoch (baseline, stimulus, delay) and inactivation condition (pre, post) in the high-gamma frequency band. Coefficients represent changes in CP relative to the baseline epoch of the post-inactivation condition, which together define the model’s reference level. The underlying numerical data are provided at https://doi.org/10.5281/zenodo.20583873.
(XLSX)

S2 Table Mixed-effects ANOVA results for low-gamma (30–70 Hz) band. Results of a linear mixed-effects model testing the interaction between behavioral epoch (baseline, stimulus, delay) and inactivation condition (pre, post) in the low-gamma frequency band. Coefficients represent changes in CP relative to the baseline epoch of the post-inactivation condition, which together define the model’s reference level. The underlying numerical data are provided at https://doi.org/10.5281/zenodo.20583873.
(XLSX)

S3 Table Mixed-effects ANOVA results for alpha-beta (5–30 Hz) band. Results of a linear mixed-effects model testing the interaction between behavioral epoch (baseline, stimulus, delay) and inactivation condition (pre, post) in the alpha-beta frequency band. Coefficients represent changes in CP relative to the baseline epoch of the post-inactivation condition, which together define the model’s reference level. The underlying numerical data are provided at https://doi.org/10.5281/zenodo.20583873.
(XLSX)

S1 Fig High-gamma (70–150 Hz) choice probability (CP) before and after cortical inactivation, shown for individual monkeys. **(A, B)** CP ± SEM traces for the two monkeys recorded in area MT. (A) Monkey Y: pre-inactivation CP = 0.511 ± 0.007 (*p* < 0.001, WSR against 0.5); post-inactivation CP = 0.502 ± 0.006 (*p* = 0.085, WSR); pre versus post inactivation comparison: *p* < 0.001 (WRS). (B) Monkey C: pre-inactivation CP = 0.507 ± 0.003 (*p* < 0.001, WSR); post-inactivation CP = 0.496 ± 0.005 (*p* < 0.001, WSR); pre versus post inactivation: *p* < 0.001 (WRS). **(C, D)** CP ± SEM traces for the two monkeys recorded in area V4. (C) Monkey L: pre-inactivation CP = 0.523 ± 0.014 (*p* < 0.001, WSR); post-inactivation CP = 0.501 ± 0.011 (*p* = 0.752, WSR); pre versus post inactivation: *p* < 0.001 (WRS). (D) Monkey A: pre-inactivation CP = 0.542 ± 0.019 (*p* < 0.001, WSR); post-inactivation CP = 0.504 ± 0.004 (*p* < 0.001, WSR); pre versus post inactivation: *p* < 0.001 (WRS). Black and blue traces denote pre- and post-inactivation sessions, respectively. The pink shaded region indicates the stimulus-response epoch (50–250 ms after stimulus onset), during which statistical comparisons were conducted. Significance markers reflect WRS tests comparing pre- and post-inactivation CP values within this epoch (*p* < 0.05 = * *p* < 0.01 = ** *p* < 0.001 = ***). Error bands represent the standard error of the mean (SEM) across sessions. The underlying numerical data are provided at https://doi.org/10.5281/zenodo.20583873.
(TIFF)

S2 Fig Low-gamma (30–70 Hz) choice probability (CP) before and after cortical inactivation, shown for individual monkeys. **(A, B)** CP ± SEM traces for the two monkeys recorded in area MT. (A) Monkey Y: pre-inactivation CP = 0.532 ± 0.009 (*p* < 0.001, WSR); post-inactivation CP = 0.535 ± 0.016 (*p* < 0.001, WSR); pre versus post inactivation: p = 0.984 (WRS). (B) Monkey C: pre-inactivation CP = 0.502 ± 0.003 (*p* = 0.155, WSR); post-inactivation CP = 0.501 ± 0.012 (*p* = 0.417, WSR); pre versus post inactivation: *p* = 0.950 (WRS). **(C, D)** CP traces for the two monkeys recorded in area V4. (C) Monkey L: pre-inactivation CP = 0.506 ± 0.015 (*p* = 0.452, WSR); post-inactivation CP = 0.509 ± 0.016 (*p* < 0.01, WSR); pre versus post inactivation: *p* = 0.192 (WRS). (D) Monkey A: pre-inactivation CP = 0.565 ± 0.040 (*p* < 0.001, WSR); post-inactivation CP = 0.538 ± 0.008 (*p* < 0.001, WSR); pre versus post inactivation: *p* = 0.384 (WRS). Black and blue traces denote pre- and post-inactivation sessions, respectively. The pink shaded region indicates the stimulus-response epoch (50–250 ms after stimulus onset), during which statistical comparisons were conducted. Significance markers reflect WRS tests comparing pre- and post-inactivation CP values within this epoch (“n.s.” denotes nonsignificant differences). Error bands represent the SEM across sessions. The underlying numerical data are provided at https://doi.org/10.5281/zenodo.20583873.
(TIFF)

S3 Fig Alpha-beta (5–30 Hz) choice probability (CP) before and after cortical inactivation, shown for individual monkeys. **(A, B)** CP ± SEM traces for the two monkeys recorded in area MT. (A) Monkey Y: pre-inactivation CP = 0.521 ± 0.018 (*p* < 0.001, WSR); post-inactivation CP = 0.493 ± 0.011 (*p* < 0.05, WSR). (B) Monkey C: pre-inactivation CP = 0.494 ± 0.003 (*p* < 0.001, WSR); post-inactivation CP = 0.492 ± 0.008 (*p* < 0.001, WSR). **(C, D)** CP traces for the two monkeys recorded in area V4. (C) Monkey L: pre-inactivation CP = 0.506 ± 0.025 (*p* = 0.180, WSR); post-inactivation CP = 0.491 ± 0.021 (*p* = 0.014, WSR); pre versus post inactivation: *p* = 0.014 (WRS). (D) Monkey A: pre-inactivation CP = 0.543 ± 0.034 (*p* < 0.001, WSR); post-inactivation CP = 0.532 ± 0.015 (*p* < 0.001, WSR); pre versus post inactivation: *p* = 0.197 (WRS). Black and blue traces denote pre- and post-inactivation sessions, respectively. The pink shaded region indicates the stimulus-response epoch (50–250 ms after stimulus onset) during which statistical comparisons were conducted. Significance markers reflect WRS tests comparing pre- and post-inactivation CP values within this epoch (*p* < 0.05 = * *p* < 0.01 = ** *p* < 0.001 = ***; n.s. = nonsignificant). Error bands represent the SEM across sessions. These results highlight the heterogeneous, monkey-specific patterns of alpha-beta decision signals. The underlying numerical data are provided at https://doi.org/10.5281/zenodo.20583873.
(TIFF)

S4 Fig Verification of normalization methods for LFP power by simulation. **(A)** A sample LFP trial trace for each choice is shown, respectively, and we simulated 100 trials for each choice using the NeuroDSP toolbox. The two choices’ averaged power spectral density (PSD) traces are shown in the log-log regime. The only difference between the two choices was the mean power of PSD across all frequencies. **(B)** Prior to normalization, the dynamic of LFP power as a function of frequency and time is shown. The patterns of the two choices looked identical, but choice 1’s PSD power levels were 10,000 times greater in amplitude than choice 2’s. **(C)** Normalization by balanced z-scoring failed to bring the two choices’ distributions of LFP power to the same scale, and PSD power level was distinctly different across two choices. **(D)** Robust scaling succeeded in normalizing LFP power. Note that the two choices shared the same scale of LFP power levels, and the LFP-based CPs computed based on these two choices were 0.5 over frequency and time. The accompanying custom code and data are provided at https://doi.org/10.5281/zenodo.20583873.
(TIFF)

S5 Fig Population decoding of stimulus in areas MT and V4. **(A, B)** A linear decoder was trained on simultaneously recorded high-gamma LFP spectra across channels to classify stimuli in (A) area MT and (B) area V4. Bars show mean ± SEM accuracy across sessions. Paired points indicate individual sessions. Filled circles represent pre-inactivation and open circles represent post-inactivation. The underlying numerical data are provided at https://doi.org/10.5281/zenodo.20583873.
(TIFF)

S6 Fig Early evoked LFP polarity before and after inactivation. **(A, B)** Circular histograms show the wrapped phase difference between post- and pre-inactivation for matched channels in (A) area MT and (B) area V4. A systematic polarity reversal computed by the Hilbert transform (2–30 Hz) would produce phase differences concentrated near π radians. Instead, (A) MT phase differences were heterogeneous and showed no significant clustering toward either 0 or π, whereas (B) V4 phase differences were significantly concentrated near 0, indicating predominant polarity preservation. Thus, the polarity inversion visible in the illustrative trace of Fig 2A was not representative across channels.
(TIFF)

S7 Fig Stimulus-evoked LFP power spectra and spectral parameterization. **(A, B)** Trial-averaged LFP power spectra computed during the stimulus-response epoch (50–250 ms after stimulus onset) are shown in solid curves before (black) and after (blue) inactivation for (A) area MT and (B) area V4. Dashed curves show the corresponding FOOOF model fits, which decompose power spectra into aperiodic and oscillatory components. **(C, D)** Histograms of fitted oscillatory peak center frequencies detected at the trial level, pooled across channels and sessions for pre- and post-inactivation conditions for (C) area MT and (D) area V4.
(TIFF)

S8 Fig Saline control example session. The session was conducted during receptive field (RF) mapping, in which the same set of images was presented before (black) and after (blue) saline injection. Each dot denotes the mean firing rate evoked by a single RF mapping image pooled across channels with isolated neurons; gray lines connect the same image pair across conditions. Semi-transparent bars indicate the group mean, error bars show SEM, and larger filled circles mark the median. The underlying numerical data are provided at https://doi.org/10.5281/zenodo.20583873.
(TIFF)

## Abbreviations

CP: choice probability
LFPs: local field potentials
MT: middle temporal
PCA: principal component analysis
PSD: power spectral density
RDKs: random dot kinematograms
ROC: receiver operating characteristic
RF: receptive field
SNR: signal-to-noise ratio
WRS: Wilcoxon rank-sum
WSR: Wilcoxon signed-rank.
