# Supplementary figures and images for: Distinct sources of decision-related signals in visual cortex are represented in different local field potential bands
Source: PLoS Biol. 2026 Jun 22;24(6):e3003873. doi: 10.1371/journal.pbio.3003873 (PMC13298987; doi:10.1371/journal.pbio.3003873)

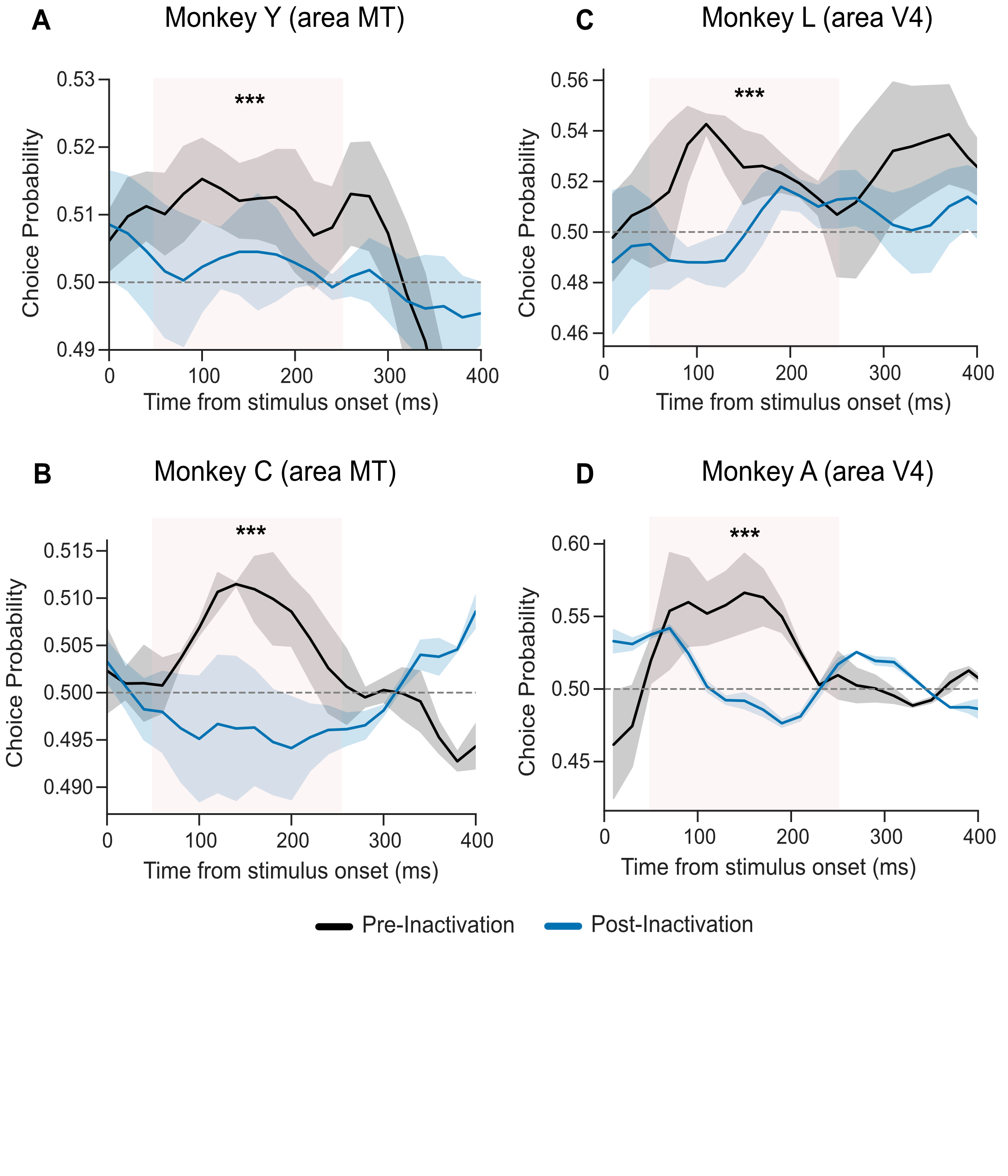

Supplement: S1 Fig — (A, B) CP ± SEM traces for the two monkeys recorded in area MT. (A) Monkey Y: pre-inactivation CP = 0.511 ± 0.007 (p < 0.001, WSR against 0.5); post-inactivation CP = 0.502 ± 0.006 (p = 0.085, WSR); pre versus post inactivation comparison: p < 0.001 (WRS). (B) Monkey C: pre-inactivation CP = 0.507 ± 0.003 (p < 0.001, WSR); post-inactivation CP = 0.496 ± 0.005 (p < 0.001, WSR); pre versus post inactivation: p < 0.001 (WRS). (C, D) CP ± SEM traces for the two monkeys recorded in area V4. (C) Monkey L: pre-inactivation CP = 0.523 ± 0.014 (p < 0.001, WSR); post-inactivation CP = 0.501 ± 0.011 (p = 0.752, WSR); pre versus post inactivation: p < 0.001 (WRS). (D) Monkey A: pre-inactivation CP = 0.542 ± 0.019 (p < 0.001, WSR); post-inactivation CP = 0.504 ± 0.004 (p < 0.001, WSR); pre versus post inactivation: p < 0.001 (WRS). Black and blue traces denote pre- and post-inactivation sessions, respectively. The pink shaded region indicates the stimulus-response epoch (50–250 ms after stimulus onset), during which statistical comparisons were conducted. Significance markers reflect WRS tests comparing pre- and post-inactivation CP values within this epoch (p < 0.05 = * p < 0.01 = ** p < 0.001 = ***). Error bands represent the standard error of the mean (SEM) across sessions. The underlying numerical data are provided at https://doi.org/10.5281/zenodo.20583873. (TIFF) [file pbio.3003873.s004.tiff]

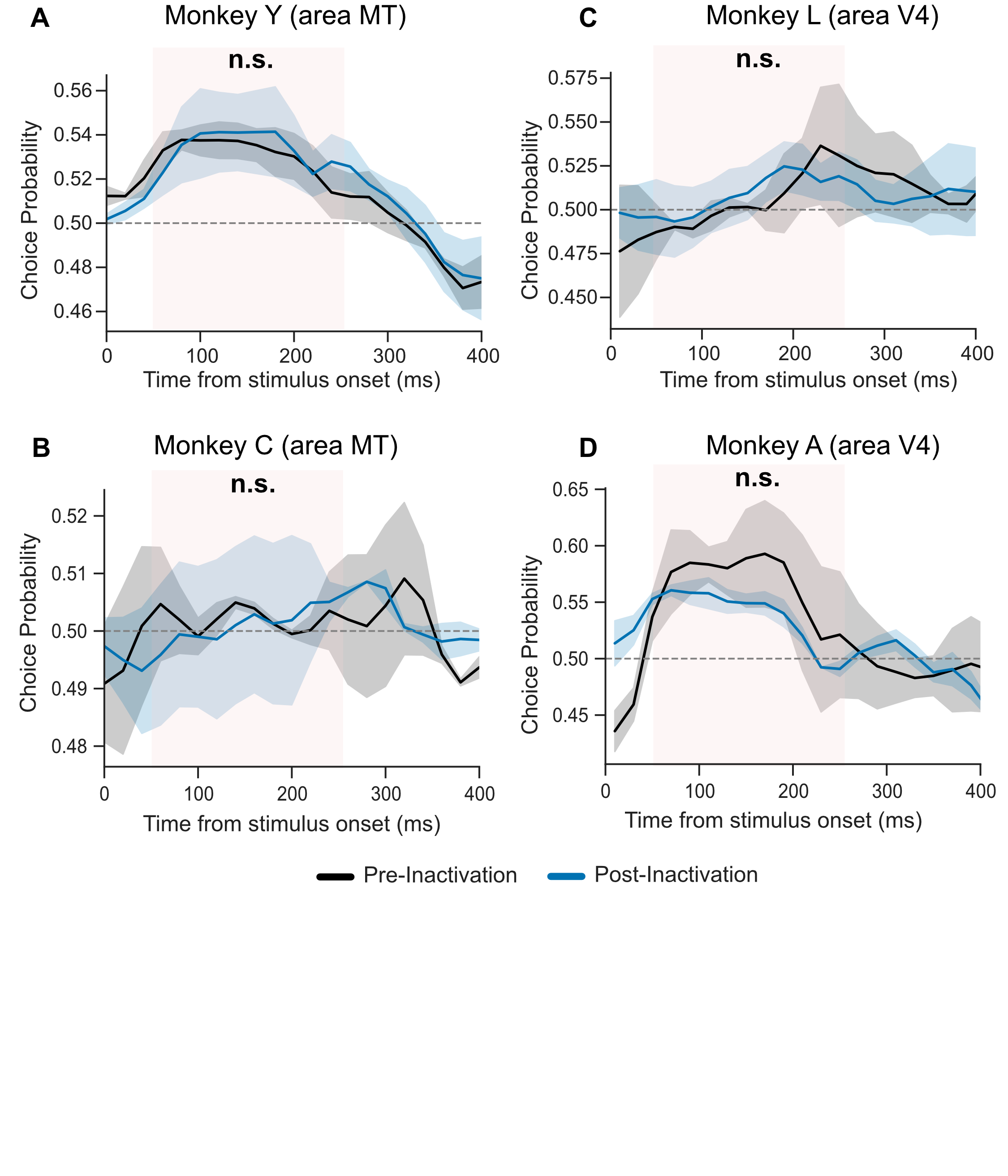

Supplement: S2 Fig — (A, B) CP ± SEM traces for the two monkeys recorded in area MT. (A) Monkey Y: pre-inactivation CP = 0.532 ± 0.009 (p < 0.001, WSR); post-inactivation CP = 0.535 ± 0.016 (p < 0.001, WSR); pre versus post inactivation: p = 0.984 (WRS). (B) Monkey C: pre-inactivation CP = 0.502 ± 0.003 (p = 0.155, WSR); post-inactivation CP = 0.501 ± 0.012 (p = 0.417, WSR); pre versus post inactivation: p = 0.950 (WRS). (C, D) CP traces for the two monkeys recorded in area V4. (C) Monkey L: pre-inactivation CP = 0.506 ± 0.015 (p = 0.452, WSR); post-inactivation CP = 0.509 ± 0.016 (p < 0.01, WSR); pre versus post inactivation: p = 0.192 (WRS). (D) Monkey A: pre-inactivation CP = 0.565 ± 0.040 (p < 0.001, WSR); post-inactivation CP = 0.538 ± 0.008 (p < 0.001, WSR); pre versus post inactivation: p = 0.384 (WRS). Black and blue traces denote pre- and post-inactivation sessions, respectively. The pink shaded region indicates the stimulus-response epoch (50–250 ms after stimulus onset), during which statistical comparisons were conducted. Significance markers reflect WRS tests comparing pre- and post-inactivation CP values within this epoch (“n.s.” denotes nonsignificant differences). Error bands represent the SEM across sessions. The underlying numerical data are provided at https://doi.org/10.5281/zenodo.20583873. (TIFF) [file pbio.3003873.s005.tiff]

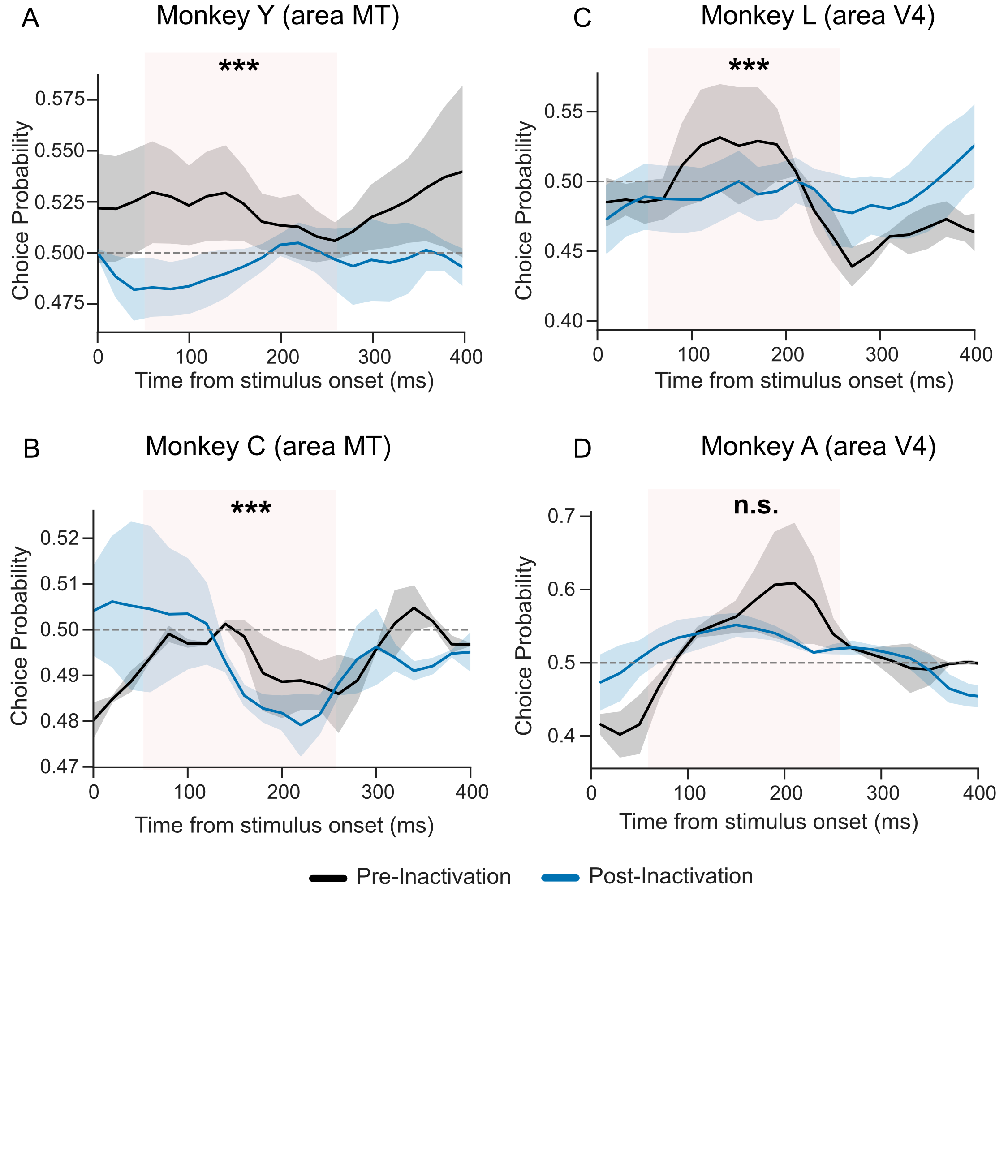

Supplement: S3 Fig — (A, B) CP ± SEM traces for the two monkeys recorded in area MT. (A) Monkey Y: pre-inactivation CP = 0.521 ± 0.018 (p < 0.001, WSR); post-inactivation CP = 0.493 ± 0.011 (p < 0.05, WSR). (B) Monkey C: pre-inactivation CP = 0.494 ± 0.003 (p < 0.001, WSR); post-inactivation CP = 0.492 ± 0.008 (p < 0.001, WSR). (C, D) CP traces for the two monkeys recorded in area V4. (C) Monkey L: pre-inactivation CP = 0.506 ± 0.025 (p = 0.180, WSR); post-inactivation CP = 0.491 ± 0.021 (p = 0.014, WSR); pre versus post inactivation: p = 0.014 (WRS). (D) Monkey A: pre-inactivation CP = 0.543 ± 0.034 (p < 0.001, WSR); post-inactivation CP = 0.532 ± 0.015 (p < 0.001, WSR); pre versus post inactivation: p = 0.197 (WRS). Black and blue traces denote pre- and post-inactivation sessions, respectively. The pink shaded region indicates the stimulus-response epoch (50–250 ms after stimulus onset) during which statistical comparisons were conducted. Significance markers reflect WRS tests comparing pre- and post-inactivation CP values within this epoch (p < 0.05 = * p < 0.01 = ** p < 0.001 = ***; n.s. = nonsignificant). Error bands represent the SEM across sessions. These results highlight the heterogeneous, monkey-specific patterns of alpha-beta decision signals. The underlying numerical data are provided at https://doi.org/10.5281/zenodo.20583873. (TIFF) [file pbio.3003873.s006.tiff]

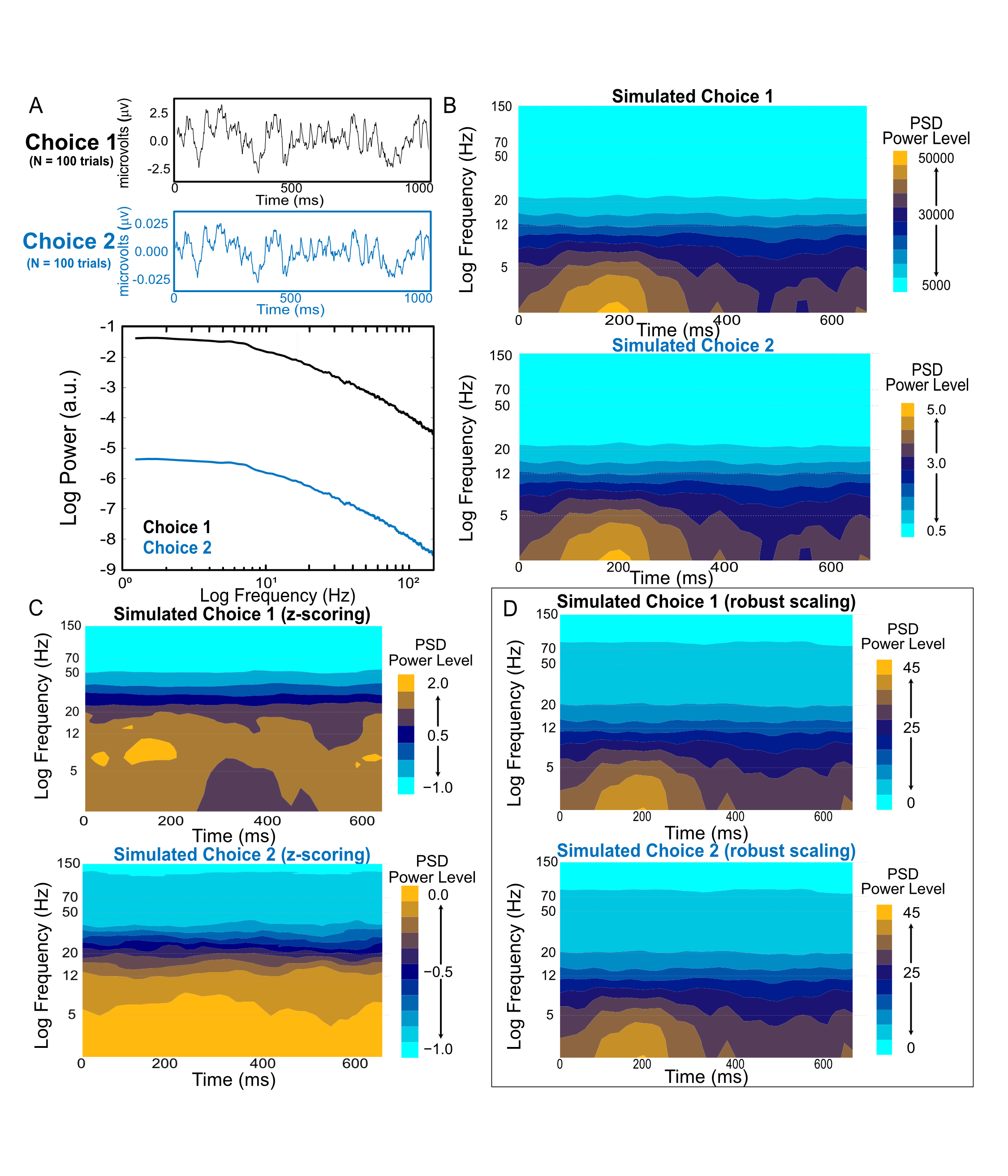

Supplement: S4 Fig — (A) A sample LFP trial trace for each choice is shown, respectively, and we simulated 100 trials for each choice using the NeuroDSP toolbox. The two choices’ averaged power spectral density (PSD) traces are shown in the log-log regime. The only difference between the two choices was the mean power of PSD across all frequencies. (B) Prior to normalization, the dynamic of LFP power as a function of frequency and time is shown. The patterns of the two choices looked identical, but choice 1’s PSD power levels were 10,000 times greater in amplitude than choice 2’s. (C) Normalization by balanced z-scoring failed to bring the two choices’ distributions of LFP power to the same scale, and PSD power level was distinctly different across two choices. (D) Robust scaling succeeded in normalizing LFP power. Note that the two choices shared the same scale of LFP power levels, and the LFP-based CPs computed based on these two choices were 0.5 over frequency and time. The accompanying custom code and data are provided at https://doi.org/10.5281/zenodo.20583873. (TIFF) [file pbio.3003873.s007.tiff]

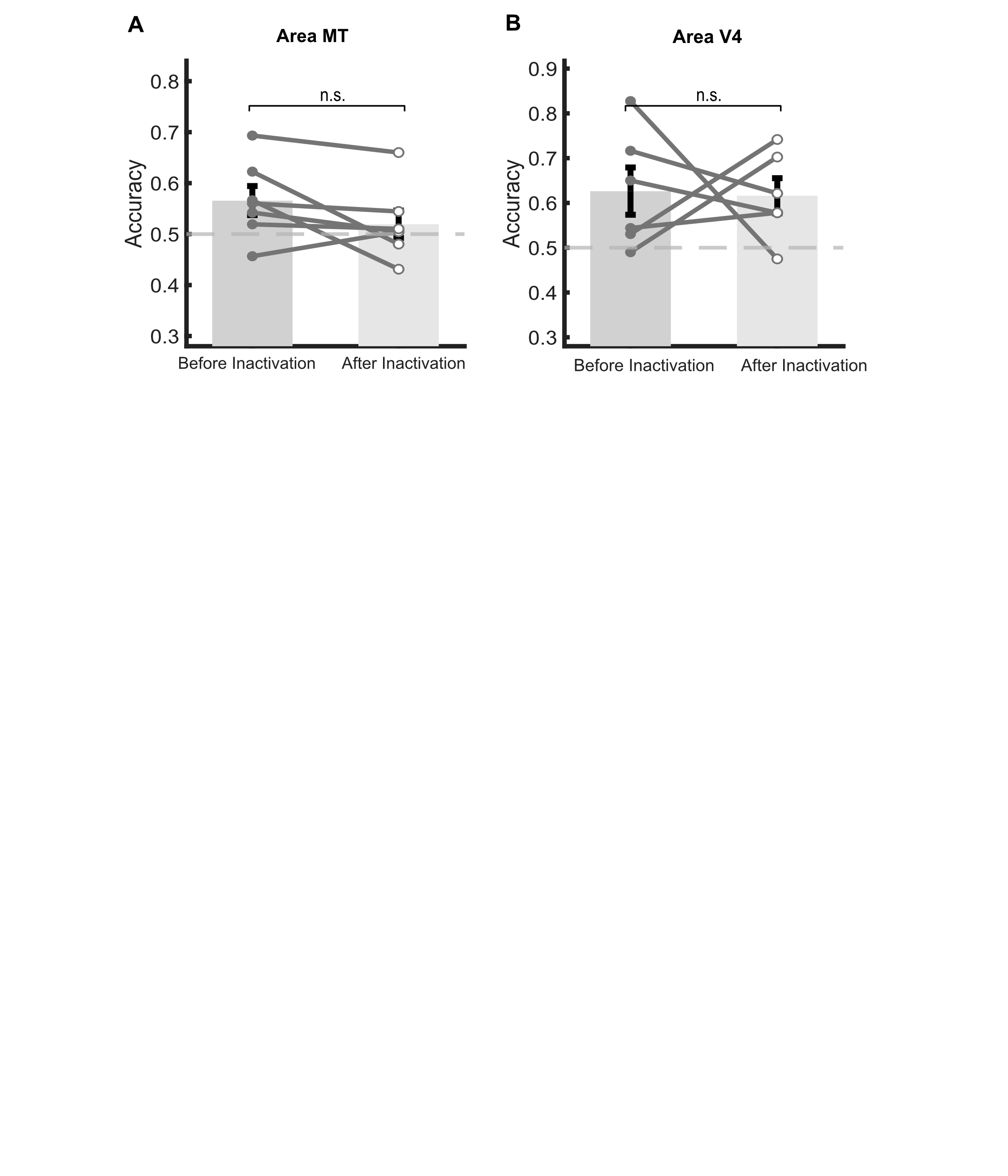

Supplement: S5 Fig — (A, B) A linear decoder was trained on simultaneously recorded high-gamma LFP spectra across channels to classify stimuli in (A) area MT and (B) area V4. Bars show mean ± SEM accuracy across sessions. Paired points indicate individual sessions. Filled circles represent pre-inactivation and open circles represent post-inactivation. The underlying numerical data are provided at https://doi.org/10.5281/zenodo.20583873. (TIFF) [file pbio.3003873.s008.tiff]

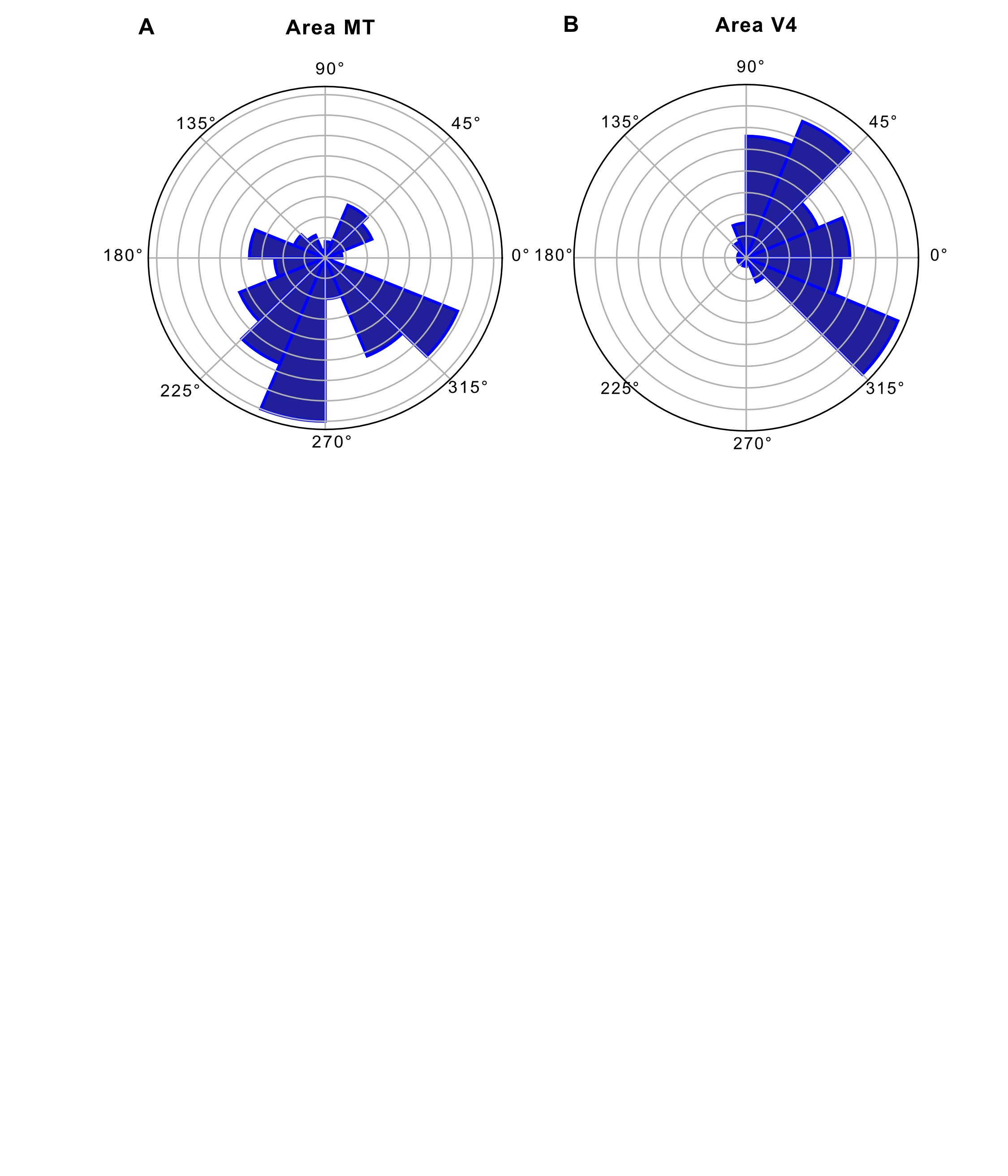

Supplement: S6 Fig — (A, B) Circular histograms show the wrapped phase difference between post- and pre-inactivation for matched channels in (A) area MT and (B) area V4. A systematic polarity reversal computed by the Hilbert transform (2–30 Hz) would produce phase differences concentrated near π radians. Instead, (A) MT phase differences were heterogeneous and showed no significant clustering toward either 0 or π, whereas (B) V4 phase differences were significantly concentrated near 0, indicating predominant polarity preservation. Thus, the polarity inversion visible in the illustrative trace of Fig 2A was not representative across channels. (TIFF) [file pbio.3003873.s009.tiff]

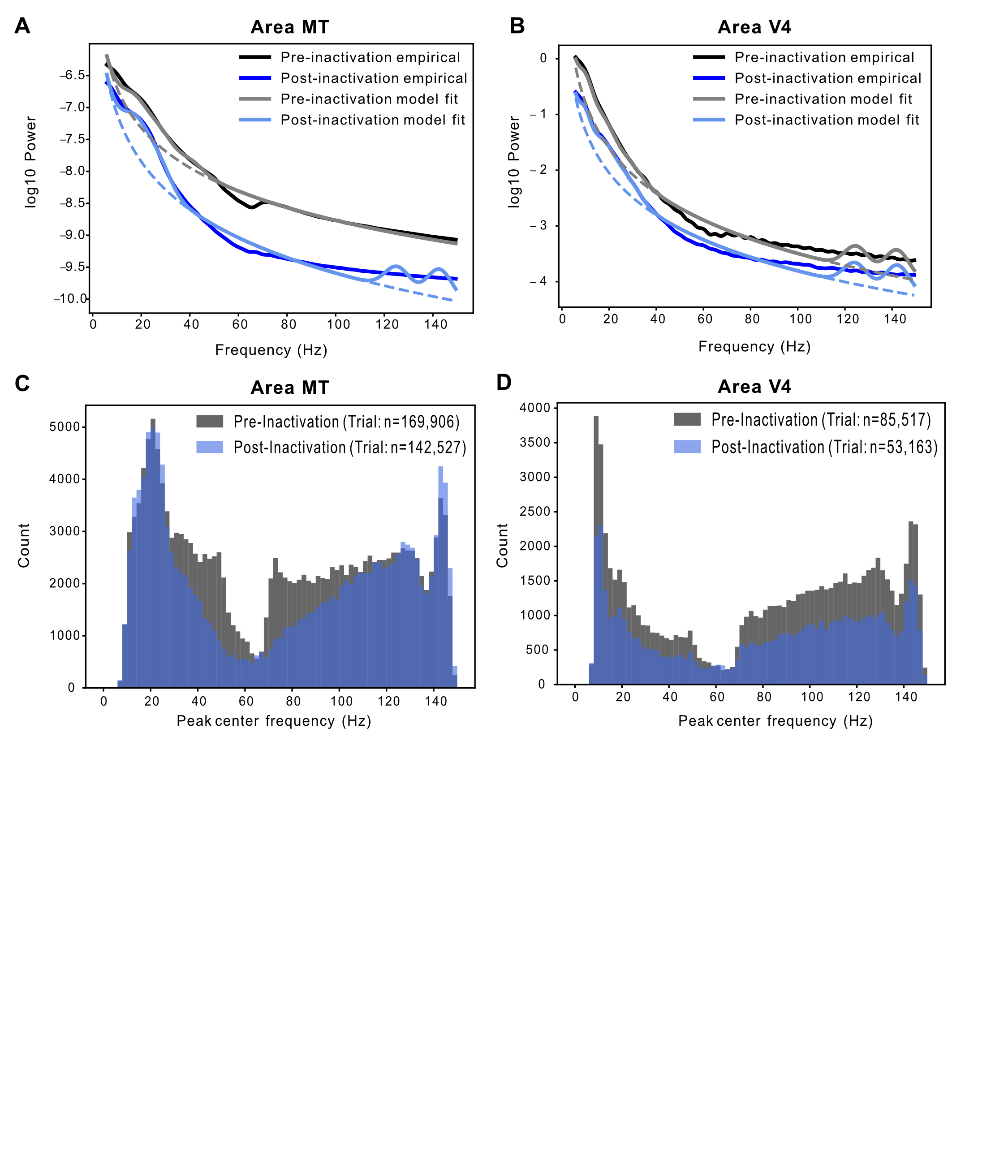

Supplement: S7 Fig — (A, B) Trial-averaged LFP power spectra computed during the stimulus-response epoch (50–250 ms after stimulus onset) are shown in solid curves before (black) and after (blue) inactivation for (A) area MT and (B) area V4. Dashed curves show the corresponding FOOOF model fits, which decompose power spectra into aperiodic and oscillatory components. (C, D) Histograms of fitted oscillatory peak center frequencies detected at the trial level, pooled across channels and sessions for pre- and post-inactivation conditions for (C) area MT and (D) area V4. (TIFF) [file pbio.3003873.s010.tiff]

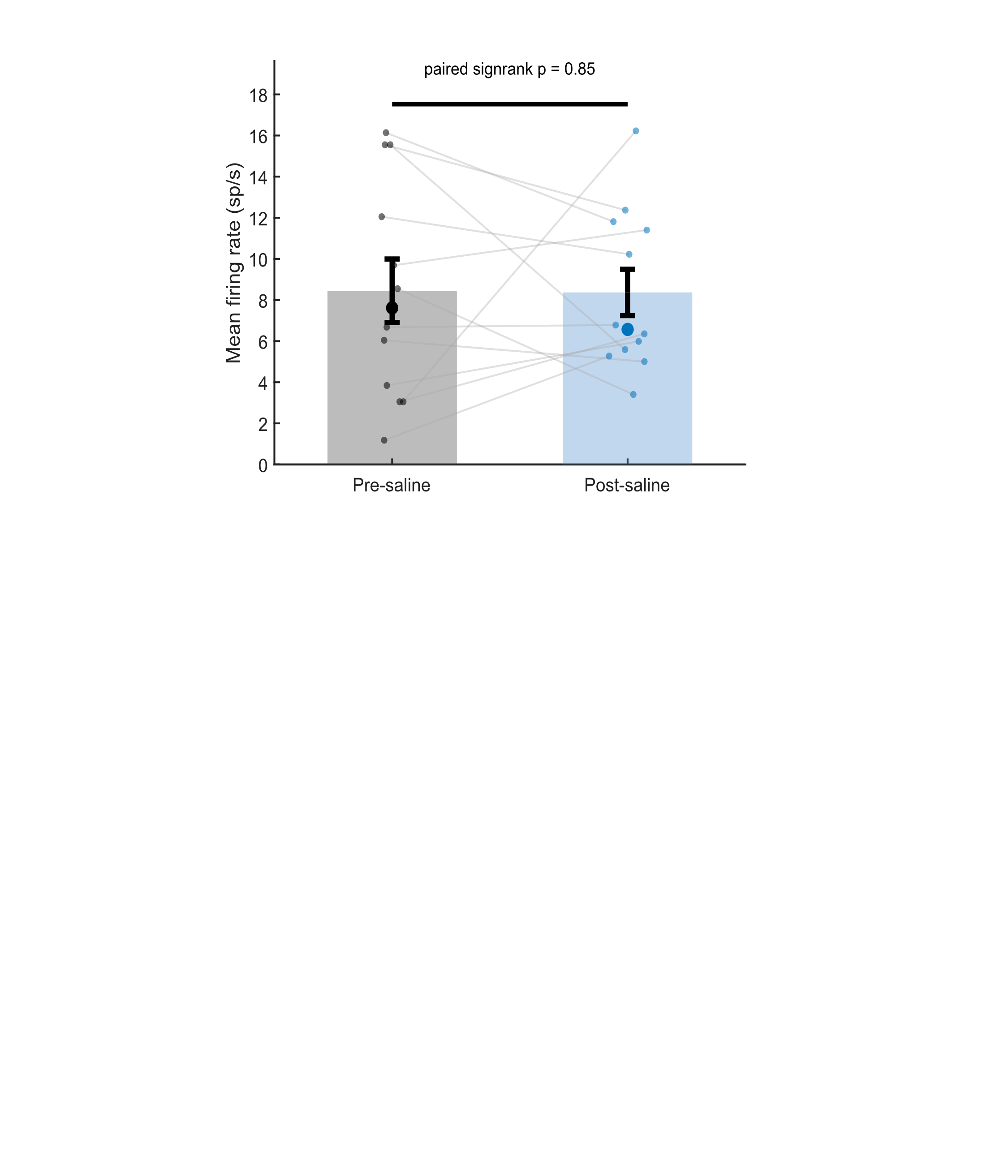

Supplement: S8 Fig — The session was conducted during receptive field (RF) mapping, in which the same set of images was presented before (black) and after (blue) saline injection. Each dot denotes the mean firing rate evoked by a single RF mapping image pooled across channels with isolated neurons; gray lines connect the same image pair across conditions. Semi-transparent bars indicate the group mean, error bars show SEM, and larger filled circles mark the median. The underlying numerical data are provided at https://doi.org/10.5281/zenodo.20583873. (TIFF) [file pbio.3003873.s011.tiff]
