# Supplementary material for: Distinct sources of decision-related signals in visual cortex are represented in different local field potential bands
Source: PLoS Biol. 2026 Jun 22;24(6):e3003873. doi: 10.1371/journal.pbio.3003873 (PMC13298987; doi:10.1371/journal.pbio.3003873)
Supplement: Attachment — Submitted filename: PBIO_response_to_reviewers_FINAL.docx [file pbio.3003873.s014.docx]

Dear Dr. Schnell and the Reviewers,

We would like to thank for your constructive feedback and suggestions on our manuscript. We were pleased to read that reviewers found that our study addresses an important question in systems neuroscience and that our approach is a “tour de force”. Below, we provide detailed point-by-point responses highlighted in blue, and corresponding quotes from the revised manuscript are indented with changes highlighted in red.

Thank you again for your time and expertise.

Sincerely,

Yueyue S. Hou, Pooya Laamerad, Liu D. Liu, Christopher C. Pack

Reviewer 1: Page 2

Reviewer 2: Page 10

Reviewer 3: Page 19

References: Page 25

**REVIEWER COMMENTS**

Reviewer #1 (signed as Michael C Schmid): Hou et al report on decision related spiking and LFP signals from areas V4 and MT of monkey visual cortex. An important aspect of their approach is the causal inactivation of these cortical areas via GABA agonist muscimol. Behaviorally, the authors show that muscimol affects perceptual decisions, and probably not performance per se. On a neural level they show how spiking and high gamma (70 - 150 Hz) activity are eliminated from the inactivation, while low-gamma (30-70 Hz) and lower frequencies (5-30 Hz) prevail. Lower frequencies also seem to reflect reward history. The work builds up on the authors' 2025 paper (Laamerad et al, Curr Biol, 2025), extends it to area MT and provides additional information from the spiking vs LFP comparison. This work has been a methodological tour de force and should be of wide interest to a large audience.

Thank you for the supportive comment.

In the following I outline my concerns with the current version.

Major:
1. The behavioural effects as presented in Figure 1 C - H are impressive, but will benefit from further clarification. Panels C-F: Are these single session examples, or summaries across experiments? Are the fits done session-by-session or together?

The behavioural effects shown in Fig. 1Panels C-F summarize behavioural performance pooled across all experimental sessions within each monkey, and a single Weibull function was fit per pre- and post- inactivation. We have now overlaid session-wise data onto the general fits.

In contrast, behavioural sensitivity in Fig.1 Panels G-H was computed from individual session-wise psychometric fits. Specifically, the Weibull function was fit separately for each session, and all statistical analyses were performed on these session-level sensitivity estimates. Now we combine these two panels into one as an updated Panel G.

This distinction is now made explicit in the Methods on line 567:

We plotted the resulting psychometric curves with 95% confidence intervals at each stimulus level using model-based prediction intervals. Pooled psychometric fits are shown in Figure 1C-F, whereas Figure 1G shows statistical analyses conducted on session-wise fits.

Fig. 1’s caption has been updated:

**(C–F)** Psychometric curves for individual monkeys before (black) and after (blue) muscimol inactivation of local spiking activity pooled across experimental sessions: **(C)** Monkey Y and **(D)** Monkey C from the MT task; **(E)** Monkey L and **(F)** Monkey A from the V4 task. Curves show model-based Weibull fits with 95% confidence intervals. Inactivation of MT or V4 caused a rightward shift in the psychometric functions, reflecting reduced sensitivity and confirming the causal contribution of these areas to their respective tasks. **(G)** Behavioral sensitivity (1/threshold) for each session and monkey. Gray lines linking paired sessions recorded on the same day. Bars represent mean ± standard error of mean (SEM) across sessions. “n.s.” denotes no significant differences that were observed between animals within either area MT or V4, indicating comparable behavioral performance across subjects.

In the main content on line 110, we add:

Figure 1C–F shows the behavioral results for each animal before and after inactivation pooled across all experimental sessions.

It would be good to see the raw data and how well they align with the fitting.

We have now overlaid session-wise behavioural data points on the psychometric curves in Figure 1C-F. Small semi-transparent dots indicate correct percentages computed separately for each session at each stimulus level; solid lines and shaded ribbons show the pooled Weibull fits and their 95% confidence intervals.

This clarification has been added to Fig.1’s caption:

Curves show model-based Weibull fits with 95% confidence intervals. Small semi-transparent dots indicate correct percentages computed separately for each session at each stimulus level.

Panels G-H: It seems the n=3 or 4 runs the risk to undersample and with a larger sample, inactivation may show a significant reduction in sensitivity.

We agree that the number of sessions per monkey is limited in same-day pharmacological inactivation experiments, and therefore this analysis was not intended to rule out inactivation-induced changes in behavioural sensitivity *per se*. Instead, they were included to illustrate the consistency of behavioural performance across monkeys and sessions within each task.

To address this concern, we have revised the corresponding paragraph to clarify this interpretation explicitly on line 117:

For each task, we quantified session-wise behavioral performance by fitting the Weibull function to generate psychometric curves (see *Methods* for details). Fig. 1G shows the sensitivity values for each monkey before and after inactivation, illustrating the consistency of behavioral performance across animals performing the same task. Specifically, sensitivity did not differ significantly between monkeys in either area MT (monkey C: 0.034 ± 0.014 vs. monkey Y: 0.034 ± 0.003; *p* = 0.492, Wilcoxon rank-sum (WRS) test) or area V4 (monkey A: 0.090 ± 0.021 vs. monkey L: 0.096 ± 0.014; *p* = 0.589, WRS).

What is the rationale for choosing the 81.6% threshold and reporting the inverse?

The choice of the 81.6% was derived from the conventional parameterization of the Weibull function. In this function,

$$\psi\left( x \right)= 100 \times(1-0.5 e^{-{(\frac{x}{\alpha})}^{\beta}})$$

the stimulus strength $x$ is normalized by the threshold parameter α. When the stimulus level $x$ is at the perceptual threshold α, independent of the slope parameter β, the equation becomes:

$$\psi\left( \alpha\right)= 100 \times\left( 1-0.5 e^{-1} \right)\approx81.6\%$$

We reported the inverse of threshold to facilitate interpretation: threshold values increase as performance worsens, whereas 1/threshold increases with sensitivity in the intuitive direction.

These details have been added to the Methods on lines 563 and 570:

where $\psi\left( x \right)$ is the predicted proportion of correct responses at stimulus level $x$ (motion coherence % for area MT and SNR % for area V4); α is the perceptual threshold, and β determines the slope of the function; and x is stimulus coherence or contrast in percentage. Under a conventional parameterization, α corresponds to $x$ yielding approximately 81.6% correct performance. Because the fit was obtained in log-percentage space, trials with 0% stimulus strength were excluded to avoid singularities under the log transform. We plotted the resulting psychometric curves with 95% confidence intervals at each stimulus level using model-based prediction intervals. Pooled psychometric fits are shown in Figure 1C-F, whereas Figure 1G shows statistical analyses conducted on session-wise fits. In Figure 1G, behavioral sensitivity was quantified as the inverse of the perceptual threshold at which behavioral performance reached 81.6% for each monkey in each recording session, such that larger values indicate better perceptual sensitivity.

2. Electrophysiology analysis: Figure 2, panel A suggests not only an amplitude reduction in the evoked LFP, but also a fundamental polarity change of the first component. I understand that panel A is an example, so one wonders whether this is consistent across experiments?

Thank you for noting the polarity change of the example trace in Figure 2A. We here quantify whether early evoked LFP polarity reversal is consistent across sessions and conditions by conducting the Hilbert transform.

A consistent polarity reversal would require the phase difference pre- and post-inactivation to cluster near π radians. If the distribution is rather broad or the cluster appears near 0 radian, then the polarity is not systematically inversed. To quantify this, for each matched channel we trial-average the evoked LFP, isolate the first evoked component by bandpass filtering the waveform from 2 to 30 Hz, and extract its phase using the Hilbert transform. We then compute the wrapped phase difference between the pre- and post-inactivation responses for each channel.

What we find is that the first evoked component is usually preserved, not reversed in V4. Same-polarity channels are 51/96 (53.1%, *p* < 0.001, permutation directional test), whereas near-reversal channels are 4/96 (4.2%, *p* = 1.0), with the phase-difference distribution concentrated near 0 rather than π (circular mean = 23.8°, R = 0.606). In MT, phase shifts are more heterogeneous (same polarity: 25/120 channels, 20.8%, *p* = 0.184; near-reversal: 25%, *p* = 0.816; other: 54.2%, *p* = 0.945), but again did not cluster near π.

We now include this control analysis in Fig. S6 and refer to it in the main text on Line 147:

In each session, we computed LFP-based CP before and after muscimol inactivation of spiking activity. Although muscimol consistently reduced spiking rates to zero (L. D. Liu & Pack, 2017), LFP activity persisted (Fig. 2A), suggesting that it contained information about input to the MT and V4 recording sites (Khawaja et al., 2009). A polarity analysis further shows that the evoked peak sign reversal in the lower panel of Fig. 2A only occurred in a subset of channels, whereas response magnitude was consistently reduced (Fig. S6). Indeed, following muscimol inactivation, LFP power in response to the visual stimuli decreased by an average of 76.0%, with no consistent difference across frequencies (Fig. 2C, Spearman’s ρ = 0.067, permutation test, *p* = 0.42).

**Fig. S6. Early evoked LFP polarity before and after inactivation. (A-B)** Circular histograms show the wrapped phase difference between post- and pre-inactivation for matched channels in **(A)** area MT and **(B)** area V4. A systematic polarity reversal computed by the Hilbert transform (2-30 Hz) would produce phase differences concentrated near π radians. Instead, (A) MT phase differences were heterogeneous and showed no significant clustering toward either 0 or π, whereas (B) V4 phase differences were significantly concentrated near 0, indicating predominant polarity preservation. Thus, the polarity inversion visible in the illustrative trace of Fig. 2A was not representative across channels.

I am not very convinced about the somewhat artificial separation into rather broad frequency bands: There is substantial evidence that broadband high-gamma power (≈70-150 Hz) is strongly influenced by action potential waveforms. Because spikes have broadband spectral content extending into the high-gamma range, this activity may reflect local population firing rate rather than an independent oscillatory LFP component. The result that muscimol eliminates then not only spiking, but also high-gamma is well expected and may reflect spike contamination, rather than an independent neural process.

We agree that broadband high-gamma power can be influenced by spike-related activity. However, we note that high-gamma LFPs in our data are not abolished by muscimol (Fig. 1C), indicating that some of the power in this range comes from other sources. Specifically, we found high-gamma power remained significantly above baseline after muscimol (one-sided WSR test, *p* < 0.05). This is not necessarily surprising, given that spike waveforms actually have very little power in any LFP band (Zanos et al., 2011). Thus, while we agree that the association between high-gamma LFPs and spiking activity is perhaps expected, this factor does not entirely explain our result.

Similarly, the evoked LFP response that provides the basis of the CP analysis (Figure 3) is typically broadband (eg Figure 2C, 50-250 ms). Before separating signals into CP frequency bands, it would be good to see the power spectrum and its consistency across experimental manipulation. If there are any oscillatory peaks, in the power spectrum, it would be good to adjust the frequency band selection accordingly.

We thank the reviewer for raising this important point regarding the spectral structure of the stimulus-evoked LFP underlying the CP analysis. To address this concern, we examine the power spectral density within 50-250 ms post-stimulus at the trial level, parameterizing each trial’s power spectrum to separate oscillatory peaks from broadband aperiodic structure using the FOOOF algorithm (Donoghue et al., 2020). The results are shown in Fig. S7:

**Figure S7. Stimulus-evoked LFP power spectra and spectral parameterization.** (A-B) Trial-averaged LFP power spectra computed during the stimulus-response epoch (50-250 ms after stimulus onset) are shown in solid curves before (black) and after (blue) inactivation for (A) area MT and (B) area V4. Dashed curves show the corresponding FOOOF model fits, which decompose power spectra into aperiodic and oscillatory components. (C-D) Histograms of fitted oscillatory peak center frequencies detected at the trial level, pooled across channels and sessions for pre- and post-inactivation conditions for (C) area MT and (D) area V4.

Across both cortical areas and experimental manipulation, LFP power spectra are well described by a dominant broadband (aperiodic) structure, with good model fits (R^2^ > 0.99 in all cases). Although modest oscillatory peaks are present, they are broadly distributed and largely consistent across conditions, with no sharp condition-specific peaks.

Because trial-level peak detection can overidentify small spectral fluctuations, particularly at higher frequency where relative power is low, we interpret the peak-frequency histograms as descriptive summaries that reveal no narrowband oscillatory peak that would motivate redefining frequency bands in a data-driven manner. We therefore retain predefined, canonical frequency bands as the operational grouping commonly used in the literature for the CP analysis.

We have now added this control analysis in the main text on line 162:

Because CP is sensitive to task difficulty (Wardak et al., 2002; Zhou & Freedman, 2019), we equated behavioral performance between pre- and post-inactivation sessions by matching stimulus difficulty levels (see *Methods* for details). We then computed LFP-based CP across three behavioral epochs: the baseline period (-200 to 0 ms, pre-stimulus fixation), the stimulus-response period (50 to 250 ms after stimulus onset), and the delay period (250 to 400 ms, prior to the presentation of two choices). Among these, the stimulus-response epoch is the most informative for interpreting CP, as this is when sensory evidence first reaches the recorded cortical sites and before the motor response is initiated. We considered CP in three canonical LFP frequency bands commonly reported in prior literature: alpha-beta (5 to 30 Hz (J. Liu & Newsome, 2006; Mendoza-Halliday et al., 2024; van Kerkoerle et al., 2014)), low gamma (30 to 70 Hz (Colgin et al., 2009; Garwood et al., 2021; Ray & Maunsell, 2011)), and high gamma (70 to 150 Hz (Canolty et al., 2006; Mendoza-Halliday et al., 2024)). Analysis of the stimulus-evoked LFP power spectra confirmed a largely broadband spectral structure before and after inactivation, with no change in narrowband oscillatory peaks (Fig. S7).

A new Methods subsection on spectral parameterization has been included on Page 14:

*Spectral parameterization of evoked LFPs:* To evaluate the spectral structure of the stimulus-evoked LFP prior to frequency band–based CP analyses, we computed power spectral density (PSD) during the stimulus-response epoch (50–250 ms post-stimulus) at the trial level. Each trial’s spectrum was parameterized using the FOOOF algorithm (Donoghue et al., 2020) over a range of 5-150 Hz, to separate broadband aperiodic structure from oscillatory peaks. To ensure robust fitting across a wide range of spectral shapes, we evaluated multiple FOOOF model configurations that varied in peak width limits (1–12 Hz), maximum number of peaks (6–8), minimum peak height (0.03–0.05), and aperiodic mode (fixed or knee). For each trial, the best-fitting model was selected based on a composite score that favored higher explained variance (R²) and lower fitting error, with a small penalty applied to excessive numbers of detected peaks. For each accepted fit, we extracted aperiodic components and oscillatory peak parameters. Peak center frequencies were pooled across trials, channels, and sessions to generate descriptive histograms of oscillatory peak distributions before and after inactivation in Fig. S7.

If this is not the case, I would advocate for a separation of low-frequencies into theta (4-8 Hz), alpha (8-12 Hz), beta (12-30 Hz) in order to better align with the literature.

We appreciate the suggestion to further subdivide the low-frequency range into theta, alpha, and beta bands to better align with literature. We note, however, that given the 200-ms analysis window imposed by trial structure shared across datasets, signals below 5 Hz may not be reliably captured if there is no full oscillatory cycle.

Nevertheless, in response to this request, we attempt to compute CP values based on the suggested bands and find the results highly similar to those obtained using our original alpha-beta band (5-30 Hz). We therefore retain our original definition while reporting the canonical subdivisions here:

| **Frequency band** | **Baseline** | **Stimulus** | **Delay** |
| --- | --- | --- | --- |
| **Theta** | Pre: 0.496 ± 0.010  Post: 0.483 ± 0.010 | Pre: 0.508 ± 0.013  Post: 0.483 ± 0.007 | Pre: 0.491 ± 0.017  Post: 0.476 ± 0.010 |
| **Alpha** | Pre: 0.494 ± 0.008  Post: 0.483 ± 0.011 | Pre: 0.507 ± 0.014  Post: 0.483 ± 0.010 | Pre: 0.488 ± 0.012  Post: 0.488 ± 0.007 |
| **Beta** | Pre: 0.495 ± 0.013  Post: 0.488 ± 0.010 | Pre: 0.525 ± 0.010  Post: 0.505 ± 0.009 | Pre: 0.505 ± 0.012  Post: 0.499 ± 0.011 |
| **Alpha–Beta** | Pre: 0.495 ± 0.010  Post: 0.487 ± 0.010 | Pre: 0.520 ± 0.009  Post: 0.499 ± 0.008 | Pre: 0.501 ± 0.012  Post: 0.494 ± 0.009 |

**Table 1.** Mean ± SEM LFP-based CP values pre- and post- inactivation, organized by frequency band (rows) and task epoch (columns), pooled across MT and V4 sessions.

Minor:
3. Figure 1, G & H: significance comparison not very graphically clear

Thanks for pointing this out. To improve clarity, we have revised Figure 1G-H to make the statistical comparison more explicit. Specifically, we now overlay individual data points for each session and clarify in the figure legend which comparisons are being tested in the updated Panel G.

4. Figure 2C: It seems to me that low frequencies might have a longer latency after inactivation.

We agree that the spectrogram in Fig. 2C can give the visual impression that low-frequency activity emerges later following inactivation. To test this explicitly, we quantify the latency of LFP power at low frequencies (5-30 Hz) by using a fraction-of-peak criterion. Specifically, we compute the baseline-corrected 5-30 Hz power time course and define latency as the first time point at which power exceeds 30% of the overall peak amplitude and remains above this threshold for at least 60 ms (30-ms step size for two consecutive time bins). We find no difference in low-frequency latency between pre- and post- inactivation (median latency = 71 ms in both conditions, *p* = 0.250, WSR). We then repeat with 20% and 40% thresholds, and the conclusion remains unchanged. Thus, muscimol primarily reduces response magnitude rather than delaying the evoked LFP onset.

5. Authors use Plexon probes. Does the muscimol injection eliminate spiking across all channels? How is this for saline injection?

Yes, in the present experiments, muscimol infusion produced complete suppression of detectable spiking activity across all channels. In contrast, saline injections at the same sites and volumes had no effect on spiking activity:

**Fig. S8. Saline control example session.** The session was conducted during receptive field mapping, in which the same set of images was presented before (black) and after (blue) saline injection. Each dot denotes the mean firing rate evoked by a single receptive field mapping image pooled across channels with isolated neurons; gray lines connect the same image pair across conditions. Semi-transparent bars indicate the group mean, error bars show SEM, and larger filled circles mark the median.

We have added this level of details to Methods on Page 13:

Behavioral testing commenced 40-50 minutes post-infusion at each site. Muscimol infusion resulted in complete suppression of detectable spiking activity across all contacts. The probes remained at the infusion depth within the cortex for the duration of the session, allowing us to verify that spiking activity remained silenced throughout the post-muscimol period, which typically lasted 2.5 to 3.5 hours. The inactivated area returned to its normal state typically within two days. Experiments involving muscimol were performed at a frequency not exceeding once per week.

As described previously (Laamerad et al., 2025; L. D. Liu & Pack, 2017), control saline injections did not alter spiking activity on any channel (Fig. S8) and had no effect on behavioral performance; there was no behavioral effect of injecting saline or of injecting muscimol at sites distant from the recording site, indicating that the muscimol results were due to local inactivation, rather than to any general effects of the injection.

6. The preservation of low-frequency LFP signals after inactivation is interesting. The authors may want to consider Schmiedt et al (J Neurosci, 2014, https://doi.org/10.1523/JNEUROSCI.0509-14.2014 for a related result in V4.

Thanks for bringing this paper into our attention. Schmiedt et al. found in V4 that beta-band activity persisted after V1 removal, so V4 beta was not dependent on feedforward V1 input. And they further discussed the possibility that the preserved or enhanced beta reflected alternative inputs, including thalamic and feedback pathways. These results complement our observation that alpha-beta and low-gamma CP persisted after inactivation, which upholds our conclusion that these signals were likely to be local output independent.

We now cite this paper in two places: first, in the Results section to support the interpretation of low-gamma CP, and second, in the Discussion to reinforce the broader interpretation that low-frequency CP reflected local-spike independent or modulatory signals on lines 228 and 376:

Thus, despite the elimination of local spiking, the continued presence of low gamma decision signals during stimulus presentation strongly suggests a component of CP that is not causally connected to behavior (Schmiedt et al., 2014).

The persistence of low-gamma CP signals (Fig. 3B) after muscimol inactivation could reflect feedback signals from decision-related areas (Cumming & Nienborg, 2016; Nienborg & Cumming, 2009; Wimmer et al., 2015). Previous studies found that low-frequency LFP components in V4 could remain intact when local spiking was strongly reduced, which suggests that such signals primarily reflected long-range synaptic input rather than local output (Kajikawa & Schroeder, 2011; Schmiedt et al., 2014). Alternatively, they could reflect shared noise correlations between neural activity in MT (or V4) and other areas, such as V3A (Nakhla et al., 2021), MST (Celebrini & Newsome, 1995), or IT (Laamerad et al., 2025). These could be feedforward in nature, in the sense that their sensory activity might inform decisions, rather than the other way around.

Reviewer #2 (signed as Valentin Dragoi): Hou et al. have investigated how decision-related signals observed in visual cortical areas should be interpreted in terms of causal versus non-causal influences on behavior. By combining simultaneous recordings of spiking activity and local field potentials (LFPs) in areas MT and V4 with pharmacological inactivation, the authors aim to dissociate the sources of choice probability (CP) across distinct frequency bands. They reported that CP in the high-gamma band is abolished following spiking inactivation, whereas CP in the low-gamma band persists, suggesting contributions from both causal and non-causal processes. In addition, the authors show that lower-frequency LFP signals reflect not only upcoming perceptual decisions but also information related to reward history. Together, these findings suggest that different components of perceptual decision-making are represented in distinct LFP frequency bands. Overall, the study addresses an important question concerning the frequency-specific organization of decision-related signals in visual cortex. Addressing the conceptual and methodological issues outlined below would further strengthen the rigor and interpretability of the conclusions.

Thanks for your comment. We hope our responses described below strengthen the rigor of our findings.

Major concerns:
1. Please note that panels D and E are missing in Figure 2.

Thanks for catching that mistake. Figures 2D and E were inadvertently retained from an earlier draft. These panels and their captions have now been fully removed.

2. The authors should quantify the latency of the signals in the various frequency bands to further quantify the feedforward and feedback nature of the decision signals observed in this study. This would help make the claims more quantitative.

We understand the rationale for this suggestion, though part of our motivation in this paper is the observation that signal timing does not reliably separate feedforward and feedback inputs (e.g., Hupé et al., 2001). In the revision we have examined latency with a threshold-free change-point analysis (Commenges & Seal, 1985; Seal et al., 1983).

As noted in our response to question 5 below, there is a numerical difference in latencies that seems to go in the expected direction. However, the difference is not statistically reliable. In other words, timing alone does not reliably dissociate the decision-related signals in our dataset. We have now added a new subsection to the Results on Page 8 to demonstrate our latency analysis, whose details are included in our response to Q5.

How we quantify latency has been added to Methods on Page 17:

*CP Latency:* We estimated CP latency using a threshold-free change-point analysis (Commenges & Seal, 1985; Seal et al., 1983). Specifically, we analyzed the CP trace from -200 to 250 ms relative to stimulus onset using a two-segment model. In this model, the CP trace was approximated by one mean level before a candidate transition time and a second mean level after that time. The transition time was constrained to occur within the post-stimulus interval (50–250 ms relative to stimulus onset). Before fitting, each trace was aligned to its dominant post-stimulus direction of deviation from 0.5 so that the main CP excursion was positive. We defined CP latency as the transition time that provided the best fit to the data, that is, the one that minimized the residual sum of squares. This procedure quantified the onset of a sustained shift in CP without relying on a fixed amplitude threshold. The latency analysis contained a 20 ms bin size, so each segment was required to include at least two samples (i.e., an effective minimum segment duration of 40 ms). We also measured the time of peak CP deviation within the same interval, defined as the time point at which the CP trace reached its maximum deviation from 0.5; the peak magnitude was defined as the corresponding maximum deviation from 0.5. Mean ± SEM summaries for latency, peak time, and peak magnitude were computed across sessions x frequency bins.

3. The authors showed that suppressing spiking activity eliminates the CP signal in the high gamma range although the LFP signal is still present. It would be helpful to additionally show using a decoder analysis, how the information content of the LFP signals change before and after inactivation that mirror the observed behavioral changes.

We appreciate this suggestion. To approach this, we ask whether stimulus information could be recovered from the population high-gamma LFP signal after inactivation. For each session, we train a linear decoder on simultaneously recorded high-gamma LFP power across channels to classify stimulus identity on a trial-by-trial basis. Analyses are restricted to correct trials and to high-evidence stimulus levels (high coherence in MT; low noise in V4), where stimulus information is expected to be the strongest.

We then compare decoding performance separately within the pre- and post-muscimol conditions. Across sessions, this analysis does not reveal a statistically significant change in high-gamma decoding after inactivation in either area. In MT, the separately trained decoders yield comparable accuracy before and after inactivation in matched sessions (pre: 0.566 ± 0.029; post: 0.519 ± 0.027; *p* = 0.156, WSR test; Fig. S5A). V4 shows a similar pattern (pre: 0.626 ± 0.053; post: 0.616 ± 0.039; *p* = 1.000, WSR test; Fig S5B). These results indicate that elimination of high-gamma CP after inactivation is not necessarily caused by a loss of stimulus coding, but rather by a block in the transmission of signals from visual cortex.

We add our approach to Methods on p.17:

## Population Decoding of Stimulus Information from LFP

We trained a simple linear decoder on simultaneously recorded high-gamma LFP spectra to classify stimulus information. For each session, we focused on correct trials and restricted to the high-evidence stimulus levels (MT coherence: 32%, 64%, 100%; V4 SNR: 25%, 60%, 100%), where stimulus information is expected to be the strongest. LFP power was computed during the stimulus response epoch (50-250 ms after stimulus onset), and features were restricted to the high-gamma band (70-150 Hz). We averaged log power within the high-gamma band for each simultaneously recorded channel, yielding one high-gamma feature per channel. Trials were balanced within each stimulus level by random downsampling. For each condition separately, a logistic regression classifier with L2 regularization (function *fitclinear*, MATLAB) was trained and evaluated using repeated stratified K-fold cross validation (5 folds, 20 repeats). Feature standardization was performed using training data only and applied to held-out test data within each condition. Decoder performance was quantified as held-out classification accuracy. For each session, the classification accuracy was first pooled across folds within each repeat and then averaged across repeats to obtain a session-level estimate. We trained decoders separately for pre- and post-inactivation data because this analysis was designed to quantify stimulus information that could be recovered from the population LFP signal within each condition. For this comparison between pre- and post-inactivation conditions, the total number of retained trials was matched across conditions within each session.

A new result paragraph is added in the main text on p.5:

In comparison to the decrease in CP, inactivation had a smaller effect on stimulus coding. A linear decoder trained on to classify stimulus identity from the population of high-gamma LFP signals (see *Methods*) maintained comparable accuracy before and after inactivation in MT (pre: 0.566 ± 0.029; post; 0.519 ± 0.027; *p* = 0.156, session-wise paired WSR test; Fig. S5A) and V4 (pre: 0.626 ± 0.053; post: 0.616 ± 0.039; *p* = 1.000, paired WSR test; Fig. S5B). Thus, the disruption of high-gamma CP with muscimol inactivation was likely not entirely due to a loss of stimulus encoding, but rather to a block in the transmission of sensory signals from visual cortex.

**Fig. S5. Population decoding of stimulus in areas MT and V4. (A-B)** A linear decoder was trained on simultaneously recorded high-gamma LFP spectra across channels to classify stimuli in **(A)** area MT and **(B)** area V4. Bar show mean ± SEM accuracy across sessions. Paired points indicate individual sessions. Filled circles represent pre-inactivation and open circles represent post-inactivation.

4. They showed that the CP signal persists in the low gamma range after inactivation and make claims about the non-causality of this signal given the drop in behavioral performance. However, can the authors comment on what happens to the information in this frequency band? Is this signal orthogonal to the readout axis that downstream areas use to make choices?

We thank the reviewer for this thoughtful question. To address it, we define a stimulus discrimination axis from the linear decoder trained on full LFP spectra during the stimulus epoch, and then quantify structure orthogonal to that axis after removing its projection. We find that this axis is consistently high-gamma weighted (high gamma: 0.618 ± 0.016 pre, 0.674 ± 0.020 post; low gamma: 0.227 ± 0.015 pre, 0.222 ± 0.011 post; $p<0.001$ in both conditions), whereas the leading null dimension orthogonal to the stimulus discrimination axis shows relatively more low-gamma weighted (low- vs. high-gamma: 0.493 ± 0.059 vs. 0.299 ± 0.046 pre; 0.482 ± 0.059 vs. 0.266 ± 0.052 post; all $p<0.05$). Thus, the persistent low-gamma signal does not primarily lie along the decoder-defined stimulus discrimination axis.

We have now added this new finding to our Results on p.6:

## Spectral Weighting on Decomposition of LFP Population Signals

Previous work has suggested that, at the population level, some decision-related signals are distinct from those that encode the relevant sensory stimuli (Zhao et al., 2020). This suggests a parcellation of population activity into stimulus-related and decision-related activity. Given the different roles for decision signals described in the previous sections, we wondered whether a similar distinction would be found in a population analysis of LFP signals.

For each session, we trained a linear classifier to discriminate stimulus identity, using the full LFP power spectrum (see *Methods*). The weight vector recovered by the decoder was taken as the stimulus axis. We then projected this axis out of the population activity and applied principal component analysis (PCA) to the residual matrix. The first principal component was taken as the leading null dimension orthogonal to the decoder-defined stimulus axis (Meirhaeghe et al., 2023; Zhao et al., 2020). We compared the spectral composition of the stimulus axis with that of the null dimension in the same population activity space (Fig. 4A).

Across areas and subjects, the stimulus axis was consistently high-gamma weighted (Fig. 4B). High-gamma loading energy on that axis was $0.618\pm0.016$ before inactivation and $0.674\pm0.020$ after inactivation, whereas low-gamma loading was substantially smaller (before: $0.227\pm0.015$, after: $0.222\pm0.011$; one-sided WSR test, *p* < 0.001 in both conditions). By contrast, the null dimension showed a different spectral tendency (Fig. 4C): low-gamma loading exceeded high-gamma loading both before and after inactivation (low- vs. high-gamma; before: $0.493\pm0.059$ vs. $0.299\pm0.046$ before inactivation; $0.482\pm0.059$ vs. $0.266\pm0.052$ after inactivation), reaching significance after inactivation (post: $p<0.05$, one-sided WSR test). This low-gamma weighting along the null dimension did not change significantly after inactivation (*p* = 0.787, two-sided WSR test).

Together, these results indicated that stimulus-aligned structure remained concentrated in high gamma, whereas the persistent low-gamma signal was preferentially expressed in a dimension orthogonal to the stimulus axis. This pattern was consistent with prior work showing that the dominant activity patterns in a source population did not necessarily coincide with the most task-relevant subspace (Semedo et al., 2019, 2020), and that choice-related signals can occupy dimensions misaligned with stimulus encoding (Laamerad et al., 2024; Zhao et al., 2020).

**Figure 4. Spectral weighting of the stimulus discrimination axis and the leading null dimension.**
**(A)** Schematic illustration of the population activity space. **(B)** Frequency-band loading energy for the stimulus axis, shown separately for low gamma and high gamma before (gray) and after (blue) inactivation. **(C)** Frequency-band loading energy for the leading null dimension, shown separately for low gamma and high gamma before (gray) and after (blue) inactivation. Bars indicate mean ± SEM across sessions, each dot represents one session, filled circles indicate before inactivation, open circles indicate after inactivation, and gray lines connect matched sessions. Asterisks denote significant comparisons and n.s. indicates a non-significant comparison.

The corresponding analytical details are available in Methods on p.18:

## Decomposition of LFP Population Signals

To compare stimulus-aligned and orthogonal structure in population LFP activity, we represented each session as a single-trial feature matrix $X\in\mathbb{R}^{T\times p}$, where each row corresponded to one trial and each column to one channel-by-frequency feature ($p=C\times F$). Features were the log-transformed stimulus-epoch LFP power values (5-150 Hz) concatenated across simultaneously recorded channels. Using this matrix, we defined two distinct types of axes.

First, we defined a decoder-based stimulus readout axis by training a linear classifier to discriminate stimulus identity. The resulting weight vector $w$ was normalized to unit length, $\hat{u}=w/\parallel w\parallel$, and taken as the stimulus axis, corresponding to the direction in population activity that best separated the two stimulus classes.

Second, to characterize activity structure not captured by that axis, we removed the projection of each trial onto the stimulus readout axis,

$$X_{\perp}=X-(X\hat{u})\hat{u}^{\top},$$

and applied principal component analysis (PCA) to the standardized residual matrix $X_{\perp}$. The first residual principal component was taken as the leading null dimension (Meirhaeghe et al., 2023; Zhao et al., 2020). To verify the geometry of this decomposition, we quantified the relationship between the null dimension $v_{\perp,1}$ and the stimulus axis $\hat{u}$ using the absolute cosine similarity, $\mid\hat{u}^{\top}v_{\perp,1}\mid$, and the corresponding angle, $\theta=arccos(\mid\hat{u}^{\top}v_{\perp,1}\mid)$, in degrees. As expected, the null dimension was orthogonal to the stimulus axis across sessions, with cosine alignment values on the order of ${10}^{-17}$ and corresponding angles of ${90}^{\circ}$.

To assess the spectral contribution of each axis, we quantified loading energy as the fraction of squared component weight assigned to features within each frequency band. For axis $k$with weight vector $v_{k}$, loading energy in band $b$ was defined as

$$E_{k,b}=\frac{\sum_{j\in I_{b}} v_{k,j}^{2}}{\sum_{j=1}^{p} v_{k,j}^{2}},$$

where $I_{b}$ denotes the set of features belonging to band $b$. Because this metric depends on squared weights, it is invariant to the arbitrary sign of the component vector.

5. Is there a statistically significant difference in the latency of the CP signal between the high and low gamma bands? One would expect that the latency should be lower for the feedforward vs the non-causal feedback signals in these 2 bands.

To address this issue, we have directly compared post-stimulus CP latency between the high- and low-gamma bands, using the threshold-free change point analysis described above (see response to Q2). Although there is a hint of evidence for longer latencies in low gamma (109.7 ± 3.6 ms in low gamma vs. 100.7 ± 2.1 ms in high gamma), these differences did not reach statistical significance (high-gamma vs. low-gamma, *p* = 0.655; WSR test).

To probe this issue further, we also compared the timing of peak CP between the two bands, but these were also not significantly different (both *p* > 0.05, WSR tests). Finally, we checked the overall magnitude of peak CP and again found no reliable difference. Thus, we do not find the dynamics of CP to be a reliable correlate of presumptive feedforward and feedback influences.

As noted above, we do not think that this non-result has direct relevance to the distinction between feedforward and feedback inputs. Indeed, this is one of our main motivations for the use of causal perturbations in the current work.

We have now added these results in the main text on p.8:

## CP Latency Across Frequency Bands

Intuitively, one might expect feedforward and feedback influences on decisions to appear at different time points in the neural response (Nienborg & Cumming, 2009; Wimmer et al., 2015). Indeed, feedforward and feedback inputs can occur with different latencies (Lamme & Roelfsema, 2000), though this is not always the case (Hupé et al., 2001). To address this issue, we quantified the latency of the emergence of CP in our data (see *Methods*). Before inactivation, the mean latencies were 100.7 ± 2.1 ms in high gamma, 109.7 ± 3.6 ms in low gamma, and 116.3 ± 4.1 ms in alpha-beta. However, paired comparisons across monkeys did not reveal reliable differences between bands (high-gamma vs. low-gamma, *p* = 0.655; high-gamma vs. alpha-beta, *p* = 0.179; low-gamma vs alpha-beta, *p* = 0.180, WSR tests). After inactivation, the mean change-point latencies were 112.1 ± 2.2 ms in high gamma, 101.9 ± 3.0 ms in low gamma, and 102.2 ± 4.4 ms in alpha-beta; again, none of the paired comparisons were statistically reliable (all *p* > 0.1). Thus, the relative temporal ordering across frequency bands was not significantly different across conditions.

Consistent with this result, the time of peak CP deviation was also similar across frequency bands. Mean peak times ranged from 144.2 to 154.1 ms before inactivation and from 138.0 to 150.1 ms after inactivation, and all paired comparisons were non-significant (all *p* > 0.1 before and after inactivation, WSR tests). Similar, the magnitudes of peak CP were not statistically different across frequency bands before or after inactivation (all *p* > 0.1). Together, neither latency nor magnitude could reliably separate mixed sources of decision-related signals in our dataset.

6. It would be intriguing to examine the spatial layout of these decision signals along the depth of the electrode. Although the authors have not performed laminar classification, however it is still possible to calculate the dependence of these signals as a function of electrode depth. This could reveal distinct aspects of feedforward and feedback signals and how it shapes behavior and could also reveal spatial heterogeneity in CP signals.

We agree that examining the spatial organization of CP signals is potentially informative. Although we are not able to perform laminar classification, we use two metrics to test whether CP varies with electrode depth.

Because probe angle and cortical geometry varied across sessions, channel index is used as a within-probe measure of relative depth. Channel-level CP values are not available for all contacts in all sessions, as CP estimation requires a minimum number of trials and balanced preferred and anti-preferred choices. Channels that did not meet these criteria were excluded.

For each session, we quantify depth dependence using two metrics:

1) a depth slope, computed as the linear regression of CP against relative channel depth, and
2) a superficial-deep contrast, defined as the difference between mean CP computed in contacts closer to the tip of the linear probe versus the ones more distal from the tip.

These analyses were performed separately for the three frequency bands, and statistics were evaluated at the session level without a strict channel pairing pre- and post-inactivation.

Across both MT and V4, we find no significant depth dependence of CP in any frequency band, either before or after inactivation. Neither depth slope nor superficial-deep contrast differs reliably from zero, and neither metric shows a consistent change following muscimol injection (all *p* > 0.05 across bands and areas). These results indicate that CP signals do not exhibit spatial heterogeneity along the electrode depth at the scale sampled by our recordings.

We have now added this piece of non-results in Discussion on line 371:

The strong reduction of high gamma CP signals following muscimol inactivation (Fig. 3A) supports the hypothesis that these signals represent feedforward processing (Lewis et al., 2021; Michalareas et al., 2016; Zanos et al., 2011). This might result from the fact that higher-frequency LFPs are generally found in the upper layers of cortex, which send feedforward projections to higher-level areas (Mendoza-Halliday et al., 2024), although we were unable to verify the laminar locations of our recordings. We attempted to examine the laminar dependency of CP signals in our data, but we found no reliable depth gradients or superficial-deep contrasts in any frequency band before and after inactivation (see *Methods*), presumably because we did not have a reliable way to estimate the laminar position of our recording channels (Hansen et al., 2012).

Its corresponding analytical approach is added in Methods on p.17:

*Depth Dependence of CP:* To test whether CP varied in terms of spatial organization, we analyzed CP at different frequency bands as a function of relative channel position along the linear probe. Because probe angle varied across sessions and laminar boundaries were not identified, channel index was treated as a within-session measure of relative depth. For each session and frequency band, we quantified depth dependence in two ways: (1) a linear slope relating CP to relative channel depth, and (2) a superficial-deep contrast defined as the difference between mean CP in contacts closer to the probe tip versus contacts away from the tip. Channels that did not have sufficient balanced trial counts for preferred and anti-preferred choices to estimate CP were excluded. Statistical tests were performed at the session level separately for each frequency band and condition.

7. In Figure 3A, B there is an increase in CP for the high and low gamma bands prior to stimulus onset for the inactivation condition. Are these changes significant and if so, what causes this increases?

In the high-gamma band, baseline CP values were close to chance in both areas and did not differ significantly from 0.5 either before or after inactivation (MT: pre = 0.503 ± 0.002, *p* > 0.1; post = 0.502 ± 0.002, *p* > 0.1; V4: pre = 0.487 ± 0.008, *p* > 0.1; post = 0.486 ± 0.008, *p* > 0.1; WSR). Moreover, baseline CP did not differ between pre- and post-inactivation conditions in either area (MT: *p* > 0.05; V4: *p* > 0.1; WRS).

Similarly, in the low-gamma band, baseline CP values were not significantly different from chance in either area (MT: pre = 0.500 ± 0.009, *p* > 0.05; post = 0.508 ± 0.005, *p* > 0.1; V4: pre = 0.488 ± 0.012, *p* > 0.1; post = 0.485 ± 0.009, *p* > 0.05; WSR), and no significant pre–post differences were observed (MT: p > 0.1; V4: *p* > 0.05; WRS).

Thus, although small fluctuations in baseline CP are visible in the time-resolved traces, these effects are not statistically reliable, are inconsistent across areas, and do not reflect a systematic increase in pre-stimulus CP values following inactivation. We therefore conclude that the decision-related effects do not arise from changes in pre-stimulus CP statistically.

8. In Figure 4 the authors quantify how reward history influences CP in the low frequency bands. However, is this effect solely due to reward history or is it a combination of both reward history and arousal. If the variables are highly correlated, then the claim that reward history is being encoded would be misleading. A simple model using both reward history and arousal (quantified via pupil diameter) could possibly answer this question. An alternative would be to match arousal levels across the 2 conditions and perform the same analysis if possible.

We thank the reviewer for raising the important possibility that reward-history effects on low-frequency CP may be mediated by arousal level, which is commonly quantified via pupil diameter. Unfortunately, pupil size cannot be obtained from either dataset.

To address this concern indirectly, we test whether the observed reward-history modulation is consistent with a global arousal state, which should be characterized by broadband and epoch-invariant neural effects (Lendner et al., 2020; Pfeffer et al., 2022). We find no evidence for such a global signature. Reward-history modulation does not generalize across frequency bands (Friedman test across alpha-beta, low-gamma, and high-gamma: χ² = 2.0, *p* = 0.37, pre- and post-inactivation), nor does it show consistent temporal effect across task epochs within the alpha-beta band (all *p* > 0.1).

These results indicate that alpha-beta CP is less likely to be a combination of both reward history and arousal, but future work to directly test this prediction with pupil size would be more helpful. Here, we claim that it is more consistent with a reward-contingent, frequency-selective internal state signal that covaries with choice.

We have now added this limitation into Discussion to guide future work on line 391:

CP signals in the alpha and beta bands were also found before and after inactivation (Fig. 3C). Given that the results were not consistent across monkeys, this alpha-beta component of CP can hardly be attributed to any mechanisms related to sensory processing. Rather, they seem to reflect the reward outcome of previous trials, as has been found for spiking activity (Lueckmann et al., 2018). One interpretation of this finding is that absence of reward transiently boosts arousal or attention, which influences both stimulus coding and task performance (Lueckmann et al., 2018; Macke & Nienborg, 2019). We could not test this possibility directly without quantifying arousal by pupil size, but we took an indirect approach assuming that arousal would affect broadband, epoch-invariant modulation (Lendner et al., 2020; Pfeffer et al., 2022). However, we still found no evidence to favor the interpretation that arousal mediated the relationship of alpha-beta CP and reward history. Alternatively, such reward-history dependent modulation may reflect changes in internal state, which would be better addressed in paradigms designed to study learning.

9. For all bar plots included in the manuscript please overlay individual data points to better represent the variability across sessions.

We have revised all bar plots to overlay individual data points. In the updated Figure 1G, overlaid points represent session-wise behavioral sensitivity estimates for each monkey. In the updated Figure 4, semi-transparent dots represent all the CP values computed within the specified time-frequency window across channels and sessions.

All corresponding figure captions have been updated to explicitly state what each overlaid data point represents:

**Fig. 1 (G).** Behavioral sensitivity (1/threshold) for each session and monkey. *Gray* lines linking paired sessions recorded on the same day. Each semi-transparent dot represents the sensitivity estimate obtained from a single session-level psychometric fit. Bars represent mean ± standard error of mean (SEM) across sessions. “n.s.” denotes no-significant differences that were observed between animals within either area MT or V4, indicating comparable behavioral performance across subjects.

**Fig. 4.** Black and blue bars indicate before and after inactivation sessions, respectively. Semi-transparent dots overlaid on each bar represent individual session-level CP values computed within the specified time-frequency window (-200 – 0 ms relative to stimulus onset, 5-30 Hz). Statistical significance markers reflect WRS tests comparing rewarded and non-rewarded trials aggregated across pre- and post-inactivation conditions within this epoch (p < 0.05, *, p < 0.01, **, p < 0.001, ***); n.s. denotes non-significant differences between reward conditions. Error bars represent the SEM across sessions.

Minor concerns

In Figure 2A, (upper panel), does the y-axis represent trial # or neuron #? It would be informative to include a figure that shows the spatial extent of suppression in the spiking activity along the depth of the electrode in addition to what is shown in the top panel of Figure 2B.

We thank the reviewer for this suggestion regarding figure clarity. In Figure 2A (upper panel), the y-axis represents trial number from a single example unit; we have now revised the panel subtitle and figure caption to explicitly indicate that this panel shows a single-unit spike raster across trials:

**Figure 2 Measuring and computing LFP-based choice probability (CP).** **(A)** A 16-channel linear electrode array was used to simultaneously record multi-unit spiking activity and local field potentials (LFPs) simultaneously from the same cortical site. Within each session, the GABAa receptor agonist muscimol was injected locally to silence spiking activity while preserving LFP signals. Example single-unit spike rasters across task trials and LFP traces from area MT illustrate the loss of spiking activity and persistence of LFP responses after inactivation. **(B)** The preferred and anti-preferred stimulus conditions for each recording site were determined from multi-unit spike counts during the stimulus-response period prior to muscimol injection (pink shading). The stimulus was either the neuron’s preferred or anti-preferred motion direction (MT) or form (V4). **(C)** Example time–frequency representations of LFP power before and after inactivation in area MT. Colors indicate power (in dB) normalized to the pre-stimulus baseline across frequencies from 0–150 Hz. LFP power decreased overall after inactivation, with a consistent change across frequencies.

We appreciate the suggestion to visualize the spatial extent of spiking suppression along the electrode depth. In the present experiments, however, spiking activity was completely suppressed across all electrode contacts by design, so there is unfortunately no electrode-wise variation in post-inactivation spiking to visualize.

Reviewer #3: General Comments:

This study valiantly attempts to make headway on an important unsolved mystery in systems neuroscience: the origin and functional role(s) of choice-predictive activity in sensory cortex. The authors approach this by analyzing correlations between local field potentials and perceptual choices, before and after chemical inactivation with muscimol. The logic is, although spiking activity (and hence the output of a local region of cortex) is abolished by the drug, some LFP power survives (reflecting the input and residual local processing), and thus any remaining correlation between LFP power and behavioral choices cannot be 'causal' in nature. Indeed they found that CPs computed from high-gamma LFP disappeared after inactivation (hence 'causal'), whereas those based on low-gamma persisted ('non-causal').

It's an interesting idea, and the study is well-executed.

Thanks for these supportive comments.

The main problem I have is that the interpretation is far from straightforward, and it may depend on one's definition (or philosophy) of causality. In this case the authors adopt a very local definition, taking the perspective of the experimenter's electrodes.

From this perspective, post-inactivation signals cannot affect behavior because there are no (measured) spikes that could carry them 'downstream'. Yet these signals are likely generated, at least in part, by distal sites that are unaffected by the drug, so they could certainly be 'causal' from the point of view of the circuits that gave rise to them.

Agreed, but we are only asking the narrower question of whether decision signals *that appear locally in visual cortex* are causally linked to behavior. This has been a topic of debate and discussion since at least the 1990s: (e.g., Britten et al., 1996: “Our interpretation is that signals from this subset of neurons are being used to form the behavioral choice.”).

We are also considering a specific alternative to Britten’s hypothesis, namely that the signals that are causally linked to behavior are echoed in visual cortex. One can of course widen this debate to encompass *any* indirect source of correlation between neural activity and behavior, but this is a general criticism of most neuroscience studies and hardly something we can tackle in a single experiment.

More generally, the cortex is not a simple feedforward system, so the conventional linear signal-processing logic may be inadequate to support the causal inferences being made.

We certainly didn’t mean to suggest that the cortex is a simple feedforward system, and in fact much of the paper is devoted to exploring the potential roles of other kinds of influences on brain activity and behavior.

Pushing on this a bit more, one may ask how *any* LFPs can be causal, according to the strict local definition. Only in the Earl-Millerian sense (i.e. ephaptic coupling) can this be so, which I don't think the authors endorse.

We also didn’t mean to suggest that LFP signals are causal, and we agree that there is no obvious way that they can be. The point was just that different anatomical sources of presynaptic input are differentially represented in different LFP frequencies, at least according to various other papers that we referenced throughout our manuscript (Fries, 2009; Michalareas et al., 2016; Richter et al., 2017).

Rather, causality here must mean 'reflects a computation that contributes to the current decision' — but this only highlights the point I am making: such a computation should be attributed to the neurons whose activity (presynaptically) generates the LFP signal in the first place, not to the extracellular voltage fluctuations at the recording/inactivation site.

One could always say that some brain signal might be attributed to computations performed elsewhere, except perhaps at the sensory periphery. This is not unique to LFPs. We are just asking about the implications of locally measured choice signals for our understanding of sensory cortex, which is also the preoccupation of most of the previous literature on the topic.

Still, it might be argued that the decomposition into frequency bands helps reify (or provide a tool to study) the putative distinct components of choice-related activity. Maybe, but then the advance here is limited by the fact that high-gamma is already known to be associated with (if not contaminated by*) spikes. So even though you can still measure some power in that band after inactivation, abolishing its correlation with behavior does not say much about the role or content of that signal, other than it is effectively a proxy for spikes, as shown in many other studies (and noted by the authors e.g. on line 206).

We agree that high-gamma is most often linked to local spiking activity, though the content of the two signals is not identical. To examine this issue more directly, we have now compared stimulus decoding performance in the high-gamma LFP in the pre- and post-muscimol conditions. Somewhat surprisingly, this analysis does not reveal a statistically significant change in stimulus decoding after inactivation in either area. In MT, decoders yield comparable performance (classification accuracy) before and after inactivation in matched sessions (pre: 0.566 ± 0.029; post: 0.519 ± 0.027; *p* = 0.156, session-wise paired WSR test; Fig. S5A). V4 shows a similar pattern (pre: 0.626 ± 0.053; post: 0.616 ± 0.039; *p* = 1.000, paired WSR test; Fig S5B). These results indicate that elimination of high-gamma CP after inactivation is not necessarily caused by a loss of stimulus coding, but rather by a block in the transmission of signals from visual cortex.

We add our approach to Methods on p.17:

## Population Decoding of Stimulus Information from LFP

We trained a simple linear decoder on simultaneously recorded high-gamma LFP spectra to classify stimulus information. For each session, we focused on correct trials and restricted to the high-evidence stimulus levels (MT coherence: 32%, 64%, 100%; V4 SNR: 25%, 60%, 100%), where stimulus information is expected to be the strongest. LFP power was computed during the stimulus response epoch (50-250 ms after stimulus onset), and features were restricted to the high-gamma band (70-150 Hz). We averaged log power within the high-gamma band for each simultaneously recorded channel, yielding one high-gamma feature per channel. Trials were balanced within each stimulus level by random downsampling. For each condition separately, a logistic regression classifier with L2 regularization (function *fitclinear*, MATLAB) was trained and evaluated using repeated stratified K-fold cross validation (5 folds, 20 repeats). Feature standardization was performed using training data only and applied to held-out test data within each condition. Decoder performance was quantified as held-out classification accuracy. For each session, the classification accuracy was first pooled across folds within each repeat and then averaged across repeats to obtain a session-level estimate. We trained decoders separately for pre- and post-inactivation data because this analysis was designed to quantify stimulus information that could be recovered from the population LFP signal within each condition. For this comparison between pre- and post-inactivation conditions, the total number of retained trials was matched across conditions within each session.

A new result paragraph is added in the main text on p.5:

In comparison to the decrease in CP, inactivation had a smaller effect on stimulus coding. A linear decoder trained on to classify stimulus identity from the population of high-gamma LFP signals (see *Methods*) maintained comparable accuracy before and after inactivation in MT (pre: 0.566 ± 0.029; post; 0.519 ± 0.027; *p* = 0.156, session-wise paired WSR test; Fig. S5A) and V4 (pre: 0.626 ± 0.053; post: 0.616 ± 0.039; *p* = 1.000, paired WSR test; Fig. S5B). Thus, the disruption of high-gamma CP with muscimol inactivation was likely not entirely due to a loss of stimulus encoding, but rather to a block in the transmission of sensory signals from visual cortex.

**Fig. S5. Population decoding of stimulus in areas MT and V4. (A-B)** A linear decoder was trained on simultaneously recorded high-gamma LFP spectra across channels to classify stimuli in **(A)** area MT and **(B)** area V4. Bar show mean ± SEM AUC across sessions. Paired points indicate individual sessions. Filled circles represent pre-inactivation and open circles represent post-inactivation.

[The alpha/beta CPs are interesting and do not suffer from the issues raised above, but the authors do not generate much new insight from this aspect of the results, other than the existence of a correlation with previous-trial reward. It would be cool to study this phenomenon in relation to learning but I realize this is beyond the current scope.]

As far as we know the finding about reward in the alpha/beta band is new, and surprising, at least to us. The other reviewers also seemed to find it interesting. We note also that the paper reveals the existence of CP in the low-gamma band after spiking inactivation, which is new and permits a quantification of the relative magnitude and timing of the presumptive feedforward and feedback sources of CP. The revised paper now includes a decomposition of population signals into stimulus-related and orthogonal dimensions (see our response to Q4).

We agree that it would be interesting to analyze the effect of learning and now mention this in the Discussion on line 395:

CP signals in the alpha and beta bands were also found before and after inactivation (Fig. 3C). Given that the results were not consistent across monkeys, this alpha-beta component of CP can hardly be attributed to any mechanisms related to sensory processing. Rather, they seem to reflect the reward outcome of previous trials, as has been found for spiking activity (Lueckmann et al., 2018). One interpretation of this finding is that absence of reward transiently boosts arousal or attention, which influences both stimulus coding and task performance (Lueckmann et al., 2018; Macke & Nienborg, 2019). Alternatively, such reward-history-dependent modulation may reflect changes in internal state, which would be better addressed in paradigms designed to study learning.

The paper concludes by saying "LFPs can be useful in revealing information about perceptual decision-making that is not readily apparent in spiking activity" — but we already knew that choice-related activity comprises multiple distinct signals with different origins, and that different frequency bands of LFP are associated with spiking activity to varying degrees. Beyond a clever and novel way to demonstrate this, I'm not sure what else we have learned.

We appreciate this point and agree that our original concluding sentence was too broad.

We now emphasize the novel scientific findings of the paper in two ways. First, we directly compared the timing and magnitude of the high- and low-gamma CP signals and found that, before inactivation, they were similar, according to our response to Reviewer 2’s Q5. This highlights the value of using LFP frequency bands to dissect different sources of choice-related activity. It suggests further that the practice of inferring these different contributions from their timing in spiking activity is not likely to be reliable, and it provides a simple alternative that should be present in most of the data collected in previous studies. Second, in a low-dimensional analysis of LFP population signals, high gamma and low gamma preferentially weighted different axes, with low gamma relatively enriched in a null dimension orthogonal to the stimulus discrimination axis that remained evident after inactivation (response to Reviewer 2’s Q4). We therefore interpret the persistent high- and low-gamma CP as distinct components of choice-related structure.

Specific/Minor:

*Re: contamination or 'bleed-through' of spike waveforms into the high-gamma band, did the authors take any steps to minimize this (for the pre-inactivation data)? The lack of a pre-post power difference in this band argues against this being a major concern, but it would be nice to add something about it in Methods.

As noted above, we actually find that the amount of stimulus information in the high-gamma LFP is unchanged by muscimol, despite the complete lack of detectable spiking activity. This suggests that the change in CP with muscimol is more due to a decreased influence of the relevant cortical areas on decisions than to the lack of spiking *per se*.

In previous work, we have shown that the direct contribution of spike waveforms to LFPs is very small, except when it is magnified by the analysis methods (spike-field correlations; Zanos et al., 2011). We now mention this in the Methods on line 684:

We statistically quantified by averaging power across three frequency bands: alpha-beta (5 to 30 Hz), low gamma (30 to 70 Hz), and high gamma (70 to 150 Hz). We noted that spike waveforms have statistically little effect on any broadband LFP power (Zanos et al., 2011).

34-35: I know this is subfield-specific shorthand, but feedforward/feedback should not be conflated with causal/non-causal. Many (perhaps most) types of feedback in the nervous system play some causal role in behavior, given a long enough time frame. But even for a given decision, a significant fraction of the feedback that drives CP could emerge prior to decision commitment and therefore contribute to it. This is part of the set of open questions that the current approach might be adapted to address in future studies.

We appreciate this comment. We have rather taken a relatively simplistic view of feedforward/feedback contributions, but we acknowledge that it is possible that feedback can be causal (Wang, 2008). Because the trials in our task were extremely short, we expect such slower recurrent contributions to be limited in the present datasets.

We have revised the Discussion to make this point explicitly for future studies to address on line 382:

The persistence of low-gamma CP signals (Fig. 3B) after muscimol inactivation could reflect feedback signals from decision-related areas (Cumming & Nienborg, 2016; Nienborg & Cumming, 2009; Wimmer et al., 2015). Previous studies found that low-frequency LFP components in V4 could remain intact when local spiking was strongly reduced, which suggests that such signals primarily reflected long-range synaptic input rather than local output (Kajikawa & Schroeder, 2011; Schmiedt et al., 2014). Alternatively, they could reflect shared noise correlations between neural activity in MT (or V4) and other areas, such as V3A (Nakhla et al., 2021), MST (Celebrini & Newsome, 1995), or IT (Laamerad et al., 2025). These could be feedforward in nature, in the sense that their sensory activity might inform decisions, rather than the other way around. We have taken a simplified view of feedforward and feedback contributions here, but feedback needs not be non-causal; in recurrent decision circuits it can help drive commitment itself, for example by accelerating ramping toward the bound (Wang, 2008), and the very short trials in our task likely limited such effects.

Some passages seem to cite the wrong literature, or at least the connection between the statement and cited work is unclear/indirect. E.g. "CP is sensitive to task difficulty [28,29]", those two are inactivation studies which do not mention CP.

We thank the reviewer for pointing this out. We agree that Wardak et al. (2002) and Zhou & Freedman (2019) did not explicitly compute choice probability and claimed that this computation is sensitive to task difficulty. Our intent in citing these works referred more generally to inactivation differences in behavioral sensitivity, and its dependence on task difficulty.

In Wardak et al. (2002), they quantified the probability of contraversive versus ipsiversive saccades as a function of target onset asynchrony (Fig. 4), providing a difficulty-dependent measure closely related to CP pre- and post-inactivation. Similarly, Zhou & Freedman (2019) used ROC-based analyses linking neural activity to behavioral choice across stimulus strengths, which is conceptually aligned with CP even if labeled differently.

To avoid ambiguity, we have revised the text to clarify that these studies demonstrate difficulty-dependent neural–behavioral correlations, rather than explicitly reporting CP on line 152:

Because neural-behavioral correlations have been shown sensitive to task difficulty (Wardak et al., 2002; Zhou & Freedman, 2019), we equated behavioral performance between pre- and post-inactivation sessions by matching stimulus difficulty levels (see *Methods* for details).

Figure 2: panels D+E, as described in the legend, sound quite useful but are omitted from this version of the figure.

Sorry, Figure 2 D+E were remnants from an earlier draft, and to avoid redundancy, we have removed these panels and their caption.

194-195: V4 is said to be no longer different from chance, yet the p value is 0.032 (< 0.05). Was a correction for multiple comparisons applied (though not mentioned in Methods)? The corresponding CP value (0.503) is barely above 0.5, so the statement made about V4 passes the 'sniff test' if not the formal hypothesis test. But this raises the additional question of how the standard errors on those CP values could be so small (0.001-0.002). SEs for a noisy measure like CP are typically on the order of 0.01 or more (as in the error bars/bands in Fig 3). How were these error values calculated, and why are many of them an order of magnitude smaller than depicted in the figure (and in the following paragraph)?

We thank the reviewer for pointing out this. The post-inactivation high-gamma CP in V4 (CP = 0.503 ± 0.001) shows a marginal deviation from 0.5 (*p* = 0.032, uncorrected). However, it does not survive correction for multiple comparisons, and its insignificance is supported by the mixed-effect ANOVA. We have therefore revised the text to clarify this result as *not reliably different from chance*, rather than categorically non-significant on line 189:

With inactivation and at an equivalent behavioral performance level, average CP values decreased significantly following inactivation (two-sided Wilcoxon rank-sum (WRS) test to compare before and after inactivation; area MT: *p* < 0.001; area V4: *p* < 0.001) and were no longer reliably different from chance, despite a marginal deviation in V4 (area MT: 0.499 ± 0.002, *p* = 0.342; area V4: 0.503 ± 0.001, *p* = 0.032; WSR test), which did not survive correction for multiple comparisons and was negligible in magnitude. These findings were consistent across individual monkeys (Supplementary Fig. 1). In contrast, inactivation had no effect on CP measured during the baseline (*p* = 1.000, WRS) or the delay (*p* = 0.597, WRS) epochs (Fig. 3A). These results were further confirmed by a linear mixed-effects ANOVA (Supplementary Table 1), in which the epoch x inactivation interaction was marginally significant in the high-gamma band (*p* = 0.049) with no other significant main or interaction effects (all other *p* > 0.2).

Regarding the small SEM values, these arise because CP values reported in the text reflect epoch-averaged CP computed at the session level, such that the SEM reflects *between-session variability*. In contrast, the shaded error bands in Figure 3 represent the SEM of time-resolved CP traces, which incorporate additional temporal variability and therefore appear larger. We have now explicitly clarified this distinction in Figure 3’s captions:

Significance markers reflect results from Wilcoxon rank-sum (WRS) tests comparing pre- and post-inactivation CP values within this epoch (p < 0.05, *, p < 0.01, **, p < 0.001, ***, “n.s.” denotes non-significant difference). Shaded regions in the left and middle panels indicate the SEM of time-resolved CP across sessions at each time point; smaller SEM values reported in the right panel and text reflect epoch-averaged CP collapsed across time.

319: merely -> hardly? unlikely to be?

We have changed the wording to “hardly”. Thanks for pointing out this phrasing, which could otherwise introduce ambiguity about the strength of the effect. The current line number is 388.

321: reward -> absence of reward? The way it's currently written would make sense if post-rewarded trials showed higher CP, whereas the opposite was true.

Indeed we meant the opposite, and the wording has been revised as suggested. The current line number is 390.

339: to what does "both sources" refer?

We have expanded this phrase to improve clarity. It now explicitly refers to as “causal and non-causal sources”. The current line number is 413.

Reference:

Canolty, R. T., Edwards, E., Dalal, S. S., Soltani, M., Nagarajan, S. S., Kirsch, H. E., Berger, M. S., Barbaro, N. M., & Knight, R. T. (2006). High Gamma Power Is Phase-Locked to Theta Oscillations in Human Neocortex. *Science*, *313*(5793), 1626–1628. https://doi.org/10.1126/science.1128115

Celebrini, S., & Newsome, W. T. (1995). Microstimulation of extrastriate area MST influences performance on a direction discrimination task. *Journal of Neurophysiology*, *73*(2), 437–448. https://doi.org/10.1152/jn.1995.73.2.437

Colgin, L. L., Denninger, T., Fyhn, M., Hafting, T., Bonnevie, T., Jensen, O., Moser, M.-B., & Moser, E. I. (2009). Frequency of gamma oscillations routes flow of information in the hippocampus. *Nature*, *462*(7271), 353–357. https://doi.org/10.1038/nature08573

Commenges, D., & Seal, J. (1985). The analysis of neuronal discharge sequences: Change-point estimation and comparison of variances. *Statistics in Medicine*, *4*(1), 91–104. https://doi.org/10.1002/sim.4780040113

Cumming, B. G., & Nienborg, H. (2016). Feedforward and feedback sources of choice probability in neural population responses. *Current Opinion in Neurobiology*, *37*, 126–132. https://doi.org/10.1016/j.conb.2016.01.009

Donoghue, T., Haller, M., Peterson, E. J., Varma, P., Sebastian, P., Gao, R., Noto, T., Lara, A. H., Wallis, J. D., Knight, R. T., Shestyuk, A., & Voytek, B. (2020). Parameterizing neural power spectra into periodic and aperiodic components. *Nature Neuroscience*, *23*(12), 1655–1665. https://doi.org/10.1038/s41593-020-00744-x

Fries, P. (2009). Neuronal Gamma-Band Synchronization as a Fundamental Process in Cortical Computation. *Annual Review of Neuroscience*, *32*(Volume 32, 2009), 209–224. https://doi.org/10.1146/annurev.neuro.051508.135603

Garwood, I. C., Chakravarty, S., Donoghue, J., Mahnke, M., Kahali, P., Chamadia, S., Akeju, O., Miller, E. K., & Brown, E. N. (2021). A hidden Markov model reliably characterizes ketamine-induced spectral dynamics in macaque local field potentials and human electroencephalograms. *PLoS Computational Biology*, *17*(8), e1009280. https://doi.org/10.1371/journal.pcbi.1009280

Hansen, B. J., Chelaru, M. I., & Dragoi, V. (2012). Correlated Variability in Laminar Cortical Circuits. *Neuron*, *76*(3), 590–602. https://doi.org/10.1016/j.neuron.2012.08.029

Hupé, J.-M., James, A. C., Girard, P., Lomber, S. G., Payne, B. R., & Bullier, J. (2001). Feedback Connections Act on the Early Part of the Responses in Monkey Visual Cortex. *Journal of Neurophysiology*, *85*(1), 134–145. https://doi.org/10.1152/jn.2001.85.1.134

Kajikawa, Y., & Schroeder, C. E. (2011). How Local Is the Local Field Potential? *Neuron*, *72*(5), 847–858. https://doi.org/10.1016/j.neuron.2011.09.029

Khawaja, F. A., Tsui, J. M. G., & Pack, C. C. (2009). Pattern Motion Selectivity of Spiking Outputs and Local Field Potentials in Macaque Visual Cortex. *The Journal of Neuroscience*, *29*(43), 13702–13709. https://doi.org/10.1523/JNEUROSCI.2844-09.2009

Laamerad, P., Krause, M. R., Guitton, D., & Pack, C. C. (2025). Inactivation of primate cortex reveals inductive biases in visual learning. *Current Biology*, S0960982225010930. https://doi.org/10.1016/j.cub.2025.08.027

Laamerad, P., Liu, L. D., & Pack, C. C. (2024). Decision-related activity and movement selection in primate visual cortex. *Science Advances*, *10*(22), eadk7214. https://doi.org/10.1126/sciadv.adk7214

Lamme, V. A. F., & Roelfsema, P. R. (2000). The distinct modes of vision offered by feedforward and recurrent processing. *Trends in Neurosciences*, *23*(11), 571–579. https://doi.org/10.1016/S0166-2236(00)01657-X

Lendner, J. D., Helfrich, R. F., Mander, B. A., Romundstad, L., Lin, J. J., Walker, M. P., Larsson, P. G., & Knight, R. T. (2020). An electrophysiological marker of arousal level in humans. *eLife*, *9*, e55092. https://doi.org/10.7554/eLife.55092

Lewis, C. M., Ni, J., Wunderle, T., Jendritza, P., Lazar, A., Diester, I., & Fries, P. (2021). Cortical gamma-band resonance preferentially transmits coherent input. *Cell Reports*, *35*(5), 109083. https://doi.org/10.1016/j.celrep.2021.109083

Liu, J., & Newsome, W. T. (2006). Local Field Potential in Cortical Area MT: Stimulus Tuning and Behavioral Correlations. *The Journal of Neuroscience*, *26*(30), 7779–7790. https://doi.org/10.1523/JNEUROSCI.5052-05.2006

Liu, L. D., & Pack, C. C. (2017). The Contribution of Area MT to Visual Motion Perception Depends on Training. *Neuron*, *95*(2), 436-446.e3. https://doi.org/10.1016/j.neuron.2017.06.024

Lueckmann, J.-M., Macke, J. H., & Nienborg, H. (2018). Can Serial Dependencies in Choices and Neural Activity Explain Choice Probabilities? *The Journal of Neuroscience*, *38*(14), 3495–3506. https://doi.org/10.1523/JNEUROSCI.2225-17.2018

Macke, J. H., & Nienborg, H. (2019). Choice (-history) correlations in sensory cortex: Cause or consequence? *Current Opinion in Neurobiology*, *58*, 148–154. https://doi.org/10.1016/j.conb.2019.09.005

Meirhaeghe, N., Riehle, A., & Brochier, T. (2023). Parallel movement planning is achieved via an optimal preparatory state in motor cortex. *Cell Reports*, *42*(2), 112136. https://doi.org/10.1016/j.celrep.2023.112136

Mendoza-Halliday, D., Major, A. J., Lee, N., Lichtenfeld, M. J., Carlson, B., Mitchell, B., Meng, P. D., Xiong, Y. (Sophy), Westerberg, J. A., Jia, X., Johnston, K. D., Selvanayagam, J., Everling, S., Maier, A., Desimone, R., Miller, E. K., & Bastos, A. M. (2024). A ubiquitous spectrolaminar motif of local field potential power across the primate cortex. *Nature Neuroscience*, *27*(3), 547–560. https://doi.org/10.1038/s41593-023-01554-7

Michalareas, G., Vezoli, J., van Pelt, S., Schoffelen, J.-M., Kennedy, H., & Fries, P. (2016). Alpha-Beta and Gamma Rhythms Subserve Feedback and Feedforward Influences among Human Visual Cortical Areas. *Neuron*, *89*(2), 384–397. https://doi.org/10.1016/j.neuron.2015.12.018

Nakhla, N., Korkian, Y., Krause, M. R., & Pack, C. C. (2021). Neural Selectivity for Visual Motion in Macaque Area V3A. *Eneuro*, *8*(1), ENEURO.0383-20.2020. https://doi.org/10.1523/ENEURO.0383-20.2020

Nienborg, H., & Cumming, B. G. (2009). Decision-related activity in sensory neurons reflects more than a neuron’s causal effect. *Nature*, *459*(7243), 89–92. https://doi.org/10.1038/nature07821

Pfeffer, T., Keitel, C., Kluger, D. S., Keitel, A., Russmann, A., Thut, G., Donner, T. H., & Gross, J. (2022). Coupling of pupil- and neuronal population dynamics reveals diverse influences of arousal on cortical processing. *eLife*, *11*, e71890. https://doi.org/10.7554/eLife.71890

Ray, S., & Maunsell, J. H. R. (2011). Different Origins of Gamma Rhythm and High-Gamma Activity in Macaque Visual Cortex. *PLoS Biology*, *9*(4), e1000610. https://doi.org/10.1371/journal.pbio.1000610

Richter, C. G., Thompson, W. H., Bosman, C. A., & Fries, P. (2017). Top-Down Beta Enhances Bottom-Up Gamma. *The Journal of Neuroscience*, *37*(28), 6698–6711. https://doi.org/10.1523/JNEUROSCI.3771-16.2017

Schmiedt, J. T., Maier, A., Fries, P., Saunders, R. C., Leopold, D. A., & Schmid, M. C. (2014). Beta Oscillation Dynamics in Extrastriate Cortex after Removal of Primary Visual Cortex. *The Journal of Neuroscience*, *34*(35), 11857–11864. https://doi.org/10.1523/JNEUROSCI.0509-14.2014

Seal, J., Commenges, D., Salamon, R., & Bioulac, B. (1983). A statistical method for the estimation of neuronal response latency and its functional interpretation. *Brain Research*, *278*(1–2), 382–386. https://doi.org/10.1016/0006-8993(83)90279-2

Semedo, J. D., Gokcen, E., Machens, C. K., Kohn, A., & Yu, B. M. (2020). Statistical methods for dissecting interactions between brain areas. *Current Opinion in Neurobiology*, *65*, 59–69. https://doi.org/10.1016/j.conb.2020.09.009

Semedo, J. D., Zandvakili, A., Machens, C. K., Yu, B. M., & Kohn, A. (2019). Cortical Areas Interact through a Communication Subspace. *Neuron*, *102*(1), 249-259.e4. https://doi.org/10.1016/j.neuron.2019.01.026

van Kerkoerle, T., Self, M. W., Dagnino, B., Gariel-Mathis, M.-A., Poort, J., van der Togt, C., & Roelfsema, P. R. (2014). Alpha and gamma oscillations characterize feedback and feedforward processing in monkey visual cortex. *Proceedings of the National Academy of Sciences*, *111*(40), 14332–14341. https://doi.org/10.1073/pnas.1402773111

Wang, X.-J. (2008). Decision Making in Recurrent Neuronal Circuits. *Neuron*, *60*(2), 215–234. https://doi.org/10.1016/j.neuron.2008.09.034

Wardak, C., Olivier, E., & Duhamel, J.-R. (2002). Saccadic Target Selection Deficits after Lateral Intraparietal Area Inactivation in Monkeys. *The Journal of Neuroscience*, *22*(22), 9877–9884. https://doi.org/10.1523/JNEUROSCI.22-22-09877.2002

Wimmer, K., Compte, A., Roxin, A., Peixoto, D., Renart, A., & De La Rocha, J. (2015). Sensory integration dynamics in a hierarchical network explains choice probabilities in cortical area MT. *Nature Communications*, *6*(1), 6177. https://doi.org/10.1038/ncomms7177

Zanos, T. P., Mineault, P. J., & Pack, C. C. (2011). Removal of Spurious Correlations Between Spikes and Local Field Potentials. *Journal of Neurophysiology*, *105*(1), 474–486. https://doi.org/10.1152/jn.00642.2010

Zhao, Y., Yates, J. L., Levi, A. J., Huk, A. C., & Park, I. M. (2020). Stimulus-choice (mis)alignment in primate area MT. *PLOS Computational Biology*, *16*(5), e1007614. https://doi.org/10.1371/journal.pcbi.1007614

Zhou, Y., & Freedman, D. J. (2019). Posterior parietal cortex plays a causal role in perceptual and categorical decisions. *Science*, *365*(6449), 180–185. https://doi.org/10.1126/science.aaw8347
