# Supplementary material for: Distinct sources of decision-related signals in visual cortex are represented in different local field potential bands
Source: PLoS Biol. 2026 Jun 22;24(6):e3003873. doi: 10.1371/journal.pbio.3003873 (PMC13298987; doi:10.1371/journal.pbio.3003873)
Supplement: Peer review history

# Editor report: Decision Letter 0

**DOI:** 10.1371/journal.pbio.3003873.r001

**Authors:** Christian Schnell, PhD

5 Dec 2025

Dear Dr Hou,

Thank you for submitting your manuscript entitled "Decision signals in the absence of spiking activity in primate visual cortex" for consideration as a Short Reports by PLOS Biology.

Your manuscript has now been evaluated by the PLOS Biology editorial staff as well as by an academic editor with relevant expertise and I am writing to let you know that we would like to send your submission out for external peer review.

However, before we can send your manuscript to reviewers, we need you to complete your submission by providing the metadata that is required for full assessment. To this end, please login to Editorial Manager where you will find the paper in the 'Submissions Needing Revisions' folder on your homepage. Please click 'Revise Submission' from the Action Links and complete all additional questions in the submission questionnaire.

Once your full submission is complete, your paper will undergo a series of checks in preparation for peer review. After your manuscript has passed the checks it will be sent out for review. To provide the metadata for your submission, please Login to Editorial Manager (https://www.editorialmanager.com/pbiology) within two working days, i.e. by Dec 09 2025 11:59PM.

If your manuscript has been previously peer-reviewed at another journal, PLOS Biology is willing to work with those reviews in order to avoid re-starting the process. Submission of the previous reviews is entirely optional and our ability to use them effectively will depend on the willingness of the previous journal to confirm the content of the reports and share the reviewer identities. Please note that we reserve the right to invite additional reviewers if we consider that additional/independent reviewers are needed, although we aim to avoid this as far as possible. In our experience, working with previous reviews does save time.

If you would like us to consider previous reviewer reports, please edit your cover letter to let us know and include the name of the journal where the work was previously considered and the manuscript ID it was given. In addition, please upload a response to the reviews as a 'Prior Peer Review' file type, which should include the reports in full and a point-by-point reply detailing how you have or plan to address the reviewers' concerns.

During the process of completing your manuscript submission, you will be invited to opt-in to posting your pre-review manuscript as a bioRxiv preprint. Visit http://journals.plos.org/plosbiology/s/preprints for full details. If you consent to posting your current manuscript as a preprint, please upload a single Preprint PDF.

Feel free to email us at plosbiology@plos.org if you have any queries relating to your submission.

Kind regards,

Christian

Christian Schnell, PhD

Senior Editor

PLOS Biology

cschnell@plos.org

# Peer review: Decision Letter 1

**DOI:** 10.1371/journal.pbio.3003873.r002

**Authors:** Christian Schnell, PhD

21 Jan 2026

Dear Dr Hou,

Thank you for your patience while your manuscript "Decision signals in the absence of spiking activity in primate visual cortex" was peer-reviewed at PLOS Biology. It has now been evaluated by the PLOS Biology editors, an Academic Editor with relevant expertise, and by several independent reviewers.

In light of the reviews, which you will find at the end of this email, we would like to invite you to revise the work to thoroughly address the reviewers' reports.

As you will see below, the reviewers agree that the topic and question that you study are interesting and important, but they also list a number of concerns that need to be addressed. Addressing the concerns from Reviewer 1 and Reviewer 2, in addition to a more nuanced interpretation of the results, will also help to alleviate Reviewer 3's concerns about the causality claims.

Given the extent of revision needed, we cannot make a decision about publication until we have seen the revised manuscript and your response to the reviewers' comments. Your revised manuscript is likely to be sent for further evaluation by all or a subset of the reviewers.

In addition to these revisions, you will need to complete some formatting changes, which you will receive in a follow up email. A member of our team will be in touch with a set of requests shortly.

We expect to receive your revised manuscript within 3 months. Please email us (plosbiology@plos.org) if you have any questions or concerns, or would like to request an extension.

At this stage, your manuscript remains formally under active consideration at our journal; please notify us by email if you do not intend to submit a revision so that we may withdraw it.

**IMPORTANT - SUBMITTING YOUR REVISION**

Your revisions should address the specific points made by each reviewer. Please submit the following files along with your revised manuscript:

1. A 'Response to Reviewers' file - this should detail your responses to the editorial requests, present a point-by-point response to all of the reviewers' comments, and indicate the changes made to the manuscript.

*NOTE: In your point-by-point response to the reviewers, please provide the full context of each review. Do not selectively quote paragraphs or sentences to reply to. The entire set of reviewer comments should be present in full and each specific point should be responded to individually, point by point.

You should also cite any additional relevant literature that has been published since the original submission and mention any additional citations in your response.

2. In addition to a clean copy of the manuscript, please also upload a 'track-changes' version of your manuscript that specifies the edits made. This should be uploaded as a "Revised Article with Changes Highlighted" file type.

*Re-submission Checklist*

When you are ready to resubmit your revised manuscript, please refer to this re-submission checklist: https://plos.io/Biology_Checklist

To submit a revised version of your manuscript, please go to https://www.editorialmanager.com/pbiology/ and log in as an Author. Click the link labelled 'Submissions Needing Revision' where you will find your submission record.

Please make sure to read the following important policies and guidelines while preparing your revision:

*Published Peer Review*

Please note while forming your response, if your article is accepted, you may have the opportunity to make the peer review history publicly available. The record will include editor decision letters (with reviews) and your responses to reviewer comments. If eligible, we will contact you to opt in or out. Please see here for more details:

https://blogs.plos.org/plos/2019/05/plos-journals-now-open-for-published-peer-review/

*PLOS Data Policy*

Please note that as a condition of publication PLOS' data policy (http://journals.plos.org/plosbiology/s/data-availability) requires that you make available all data used to draw the conclusions arrived at in your manuscript. If you have not already done so, you must include any data used in your manuscript either in appropriate repositories, within the body of the manuscript, or as supporting information (N.B. this includes any numerical values that were used to generate graphs, histograms etc.). For an example see here: http://www.plosbiology.org/article/info%3Adoi%2F10.1371%2Fjournal.pbio.1001908#s5

*Blot and Gel Data Policy*

We require the original, uncropped and minimally adjusted images supporting all blot and gel results reported in an article's figures or Supporting Information files. We will require these files before a manuscript can be accepted so please prepare them now, if you have not already uploaded them. Please carefully read our guidelines for how to prepare and upload this data: https://journals.plos.org/plosbiology/s/figures#loc-blot-and-gel-reporting-requirements

*Protocols deposition*

To enhance the reproducibility of your results, we recommend that if applicable you deposit your laboratory protocols in protocols.io, where a protocol can be assigned its own identifier (DOI) such that it can be cited independently in the future. Additionally, PLOS ONE offers an option for publishing peer-reviewed Lab Protocol articles, which describe protocols hosted on protocols.io. Read more information on sharing protocols at https://plos.org/protocols?utm_medium=editorial-email&utm_source=authorletters&utm_campaign=protocols

Thank you again for your submission to our journal. We hope that our editorial process has been constructive thus far, and we welcome your feedback at any time. Please don't hesitate to contact us if you have any questions or comments.

Sincerely,

Christian

Christian Schnell, PhD

Senior Editor

PLOS Biology

cschnell@plos.org

------------------------------------

REVIEWS:

Reviewer #1 (signed as Michael C Schmid): Hou et al report on decision related spiking and LFP signals from areas V4 and MT of monkey visual cortex. An important aspect of their approach is the causal inactivation of these cortical areas via GABA agonist muscimol. Behaviorally, the authors show that muscimol affects perceptual decisions, and probably not performance per se. On a neural level they show how spiking and high gamma (70 - 150 Hz) activity are eliminated from the inactivation, while low-gamma (30-70 Hz) and lower frequencies (5-30 Hz) prevail. Lower frequencies also seem to reflect reward history. The work builds up on the authors' 2025 paper (Laamerad et al, Curr Biol, 2025), extends it to area MT and provides additional information from the spiking vs LFP comparison. This work has been a methodological tour de force and should be of wide interest to a large audience. In the following I outline my concerns with the current version.

Major:

1. The behavioural effects as presented in Figure 1 C - H are impressive, but will benefit from further clarification. Panels C-F: Are these single session examples, or summaries across experiments? Are the fits done session-by-session or together? It would be good to see the raw data and how well they align with the fitting. Panels G-H: It seems the n=3 or 4 runs the risk to undersample and with a larger sample, inactivation may show a significant reduction in sensitivity. What is the rationale for choosing the 81.6% threshold and reporting the inverse?

2. Electrophysiology analysis: Figure 2, panel A suggests not only an amplitude reduction in the evoked LFP, but also a fundamental polarity change of the first component. I understand that panel A is an example, so one wonders whether this is consistent across experiments?

I am not very convinced about the somewhat artificial separation into rather broad frequency bands: There is substantial evidence that broadband high-gamma power (≈70-150 Hz) is strongly influenced by action potential waveforms. Because spikes have broadband spectral content extending into the high-gamma range, this activity may reflect local population firing rate rather than an independent oscillatory LFP component. The result that muscimol eliminates then not only spiking, but also high-gamma is well expected and may reflect spike contamination, rather than an independent neural process.

Similarly, the evoked LFP response that provides the basis of the CP analysis (Figure 3) is typically broadband (eg Figure 2C, 50-250 ms). Before separating signals into CP frequency bands, it would be good to see the power spectrum and its consistency across experimental manipulation. If there are any oscillatory peaks, in the power spectrum, it would be good to adjust the frequency band selection accordingly. If this is not the case, I would advocate for a separation of low-frequencies into theta (4-8 Hz), alpha (8-12 Hz), beta (12-30 Hz) in order to better align with the literature.

Minor:

3. Figure 1, G & H: significance comparison not very graphically clear

4. Figure 2C: It seems to me that low frequencies might have a longer latency after inactivation.

5. Authors use Plexon probes. Does the muscimol injection eliminate spiking across all channels? How is this for saline injection?

6. The preservation of low-frequency LFP signals after inactivation is interesting. The authors may want to consider Schmiedt et al (J Neurosci 2014, https://doi.org/10.1523/JNEUROSCI.0509-14.2014) for a related result in V4.

Reviewer #2 (signed as Valentin Dragoi): Hou et al. have investigated how decision-related signals observed in visual cortical areas

should be interpreted in terms of causal versus non-causal influences on behavior. By combining

simultaneous recordings of spiking activity and local field potentials (LFPs) in areas MT and V4

with pharmacological inactivation, the authors aim to dissociate the sources of choice probability

(CP) across distinct frequency bands. They reported that CP in the high-gamma band is abolished

following spiking inactivation, whereas CP in the low-gamma band persists, suggesting

contributions from both causal and non-causal processes. In addition, the authors show that

lower-frequency LFP signals reflect not only upcoming perceptual decisions but also information

related to reward history. Together, these findings suggest that different components of

perceptual decision-making are represented in distinct LFP frequency bands. Overall, the study

addresses an important question concerning the frequency-specific organization of decision-

related signals in visual cortex. Addressing the conceptual and methodological issues outlined

below would further strengthen the rigor and interpretability of the conclusions.

Major concerns:

1. Please note that panels D and E are missing in Figure 2.

2. The authors should quantify the latency of the signals in the various frequency bands to further

quantify the feedforward and feedback nature of the decision signals observed in this study. This

would help make the claims more quantitative.

3. The authors showed that suppressing spiking activity eliminates the CP signal in the high gamma

range although the LFP signal is still present. It would be helpful to additionally show using a

decoder analysis, how the information content of the LFP signals change before and after

inactivation that mirror the observed behavioral changes.

4. They showed that the CP signal persists in the low gamma range after inactivation and

make claims about the non-causality of this signal given the drop in behavioral performance.

However, can the authors comment on what happens to the information in this frequency band? Is this

signal orthogonal to the readout axis that downstream areas use to make choices?

5. Is there a statistically significant difference in the latency of the CP signal between the high

and low gamma bands? One would expect that the latency should be lower for the feedforward

vs the non-causal feedback signals in these 2 bands.

6. It would be intriguing to examine the spatial layout of these decision signals along the depth of

the electrode. Although the authors have not performed laminar classification, however it is still

possible to calculate the dependence of these signals as a function of electrode depth. This could

reveal distinct aspects of feedforward and feedback signals and how it shapes behavior and could

also reveal spatial heterogeneity in CP signals.

7. In Figure 3A, B there is an increase in CP for the high and low gamma bands prior to stimulus

onset for the inactivation condition. Are these changes significant and if so, what causes this

increases?

8. In Figure 4 the authors quantify how reward history influences CP in the low frequency bands.

However, is this effect solely due to reward history or is it a combination of both reward history

and arousal. If the variables are highly correlated, then the claim that reward history is being

encoded would be misleading. A simple model using both reward history and arousal (quantified

via pupil diameter) could possibly answer this question. An alternative would be to match

arousal levels across the 2 conditions and perform the same analysis if possible.

9. For all bar plots included in the manuscript please overlay individual data points to better

represent the variability across sessions.

Minor concerns

In Figure 2A, (upper panel), does the y-axis represent trial # or neuron #?

It would be informative to include a figure that shows the spatial extent of suppression in the

spiking activity along the depth of the electrode in addition to what is shown in the top panel of

Figure 2B.

Reviewer #3: General Comments:

This study valiantly attempts to make headway on an important unsolved mystery in systems neuroscience: the origin and functional role(s) of choice-predictive activity in sensory cortex. The authors approach this by analyzing correlations between local field potentials and perceptual choices, before and after chemical inactivation with muscimol. The logic is, although spiking activity (and hence the output of a local region of cortex) is abolished by the drug, some LFP power survives (reflecting the input and residual local processing), and thus any remaining correlation between LFP power and behavioral choices cannot be 'causal' in nature. Indeed they found that CPs computed from high-gamma LFP disappeared after inactivation (hence 'causal'), whereas those based on low-gamma persisted ('non-causal').

It's an interesting idea, and the study is well-executed. The main problem I have is that the interpretation is far from straightforward, and it may depend on one's definition (or philosophy) of causality. In this case the authors adopt a very local definition, taking the perspective of the experimenter's electrodes. From this perspective, post-inactivation signals cannot affect behavior because there are no (measured) spikes that could carry them 'downstream'. Yet these signals are likely generated, at least in part, by distal sites that are unaffected by the drug, so they could certainly be 'causal' from the point of view of the circuits that gave rise to them. More generally, the cortex is not a simple feedforward system, so the conventional linear signal-processing logic may be inadequate to support the causal inferences being made.

Pushing on this a bit more, one may ask how *any* LFPs can be causal, according to the strict local definition. Only in the Earl-Millerian sense (i.e. ephaptic coupling) can this be so, which I don't think the authors endorse. Rather, causality here must mean 'reflects a computation that contributes to the current decision' — but this only highlights the point I am making: such a computation should be attributed to the neurons whose activity (presynaptically) generates the LFP signal in the first place, not to the extracellular voltage fluctuations at the recording/inactivation site.

Still, it might be argued that the decomposition into frequency bands helps reify (or provide a tool to study) the putative distinct components of choice-related activity. Maybe, but then the advance here is limited by the fact that high-gamma is already known to be associated with (if not contaminated by*) spikes. So even though you can still measure some power in that band after inactivation, abolishing its correlation with behavior does not say much about the role or content of that signal, other than it is effectively a proxy for spikes, as shown in many other studies (and noted by the authors e.g. on line 206).

[The alpha/beta CPs are interesting and do not suffer from the issues raised above, but the authors do not generate much new insight from this aspect of the results, other than the existence of a correlation with previous-trial reward. It would be cool to study this phenomenon in relation to learning but I realize this is beyond the current scope.]

The paper concludes by saying "LFPs can be useful in revealing information about perceptual decision-making that is not readily apparent in spiking activity" — but we already knew that choice-related activity comprises multiple distinct signals with different origins, and that different frequency bands of LFP are associated with spiking activity to varying degrees. Beyond a clever and novel way to demonstrate this, I'm not sure what else we have learned.

Specific/Minor:

*Re: contamination or 'bleed-through' of spike waveforms into the high-gamma band, did the authors take any steps to minimize this (for the pre-inactivation data)? The lack of a pre-post power difference in this band argues against this being a major concern, but it would be nice to add something about it in Methods.

34-35: I know this is subfield-specific shorthand, but feedforward/feedback should not be conflated with causal/noncausal. Many (perhaps most) types of feedback in the nervous system play some causal role in behavior, given a long enough time frame. But even for a given decision, a significant fraction of the feedback that drives CP could emerge prior to decision commitment and therefore contribute to it. This is part of the set of open questions that the current approach might be adapted to address in future studies.

Some passages seem to cite the wrong literature, or at least the connection between the statement and cited work is unclear/indirect. E.g. "CP is sensitive to task difficulty [28,29]", those two are inactivation studies which do not mention CP.

Figure 2: panels D+E, as described in the legend, sound quite useful but are omitted from this version of the figure.

194-195: V4 is said to be no longer different from chance, yet the p value is 0.032 (< 0.05). Was a correction for multiple comparisons applied (though not mentioned in Methods)? The corresponding CP value (0.503) is barely above 0.5, so the statement made about V4 passes the 'sniff test' if not the formal hypothesis test. But this raises the additional question of how the standard errors on those CP values could be so small (0.001-0.002). SEs for a noisy measure like CP are typically on the order of 0.01 or more (as in the error bars/bands in Fig 3). How were these error values calculated, and why are many of them an order of magnitude smaller than depicted in the figure (and in the following paragraph)?

319: merely -> hardly? unlikely to be?

321: reward -> absence of reward? The way it's currently written would make sense if post-rewarded trials showed higher CP, whereas the opposite was true.

339: to what does "both sources" refer?

# Author comment: Author response to Decision Letter 2

**DOI:** 10.1371/journal.pbio.3003873.r003

19 Apr 2026

Attachment Submitted filename: PBIO_response_to_reviewers_FINAL.docx

# Peer review: Decision Letter 2

**DOI:** 10.1371/journal.pbio.3003873.r004

**Authors:** Christian Schnell, PhD

29 May 2026

Dear Dr Hou,

Thank you for your patience while we considered your revised manuscript "Decision signals in the absence of spiking activity in primate visual cortex" for publication as a Short Reports at PLOS Biology. This revised version of your manuscript has been evaluated by the PLOS Biology editors, the Academic Editor and the original reviewers.

Based on the reviews and on our Academic Editor's assessment of your revision, we are likely to accept this manuscript for publication, provided you satisfactorily address the following data and other policy-related requests:

* We would like to suggest a different title to improve its accessibility for our broad audience:

Causal and non-causal components of decision-related signals in visual cortex are represented in distinct local field potential bands

* Please add the links to the funding agencies in the Financial Disclosure statement in the manuscript details.

* Please mention in the competing interests statement that Christopher C Pack is a member our PLOS Biology's editorial board.

* Please note that per journal policy, the model system/species studied should be clearly stated in the abstract of your manuscript.

* We can allow only four figures for Short Reports. Please reduce the number of figures by one, maybe by combining figures 4 and 5?

* DATA POLICY:

You may be aware of the PLOS Data Policy, which requires that all data be made available without restriction: http://journals.plos.org/plosbiology/s/data-availability. For more information, please also see this editorial: http://dx.doi.org/10.1371/journal.pbio.1001797

Note that we do not require all raw data. Rather, we ask that all individual quantitative observations that underlie the data summarized in the figures and results of your paper be made available in one of the following forms:

1) Supplementary files (e.g., excel). Please ensure that all data files are uploaded as 'Supporting Information' and are invariably referred to (in the manuscript, figure legends, and the Description field when uploading your files) using the following format verbatim: S1 Data, S2 Data, etc. Multiple panels of a single or even several figures can be included as multiple sheets in one excel file that is saved using exactly the following convention: S1_Data.xlsx (using an underscore).

2) Deposition in a publicly available repository. Please also provide the accession code or a reviewer link so that we may view your data before publication.

Regardless of the method selected, please ensure that you provide the individual numerical values that underlie the summary data displayed in the following figure panels as they are essential for readers to assess your analysis and to reproduce it: 1G, 4BC, 5ABCD, S5AB and S8

NOTE: the numerical data provided should include all replicates AND the way in which the plotted mean and errors were derived (it should not present only the mean/average values).

Please also ensure that figure legends in your manuscript include information on where the underlying data can be found, and ensure your supplemental data file/s has a legend.

Please ensure that your Data Statement in the submission system accurately describes where your data can be found.

* CODE POLICY

Per journal policy, if you have generated any custom code during the course of this investigation, please make it available without restrictions. Please ensure that the code is sufficiently well documented and reusable, and that your Data Statement in the Editorial Manager submission system accurately describes where your code can be found. More information on our Code Policy, what and how to share can be found here: https://journals.plos.org/plosbiology/s/code-availability

Please note that we cannot accept sole deposition of code in GitHub, as this could be changed after publication. However, you can archive this version of your publicly available GitHub code to Zenodo. Once you do this, it will generate a DOI number, which you will need to provide in the Data Accessibility Statement (you are welcome to also provide the GitHub access information). See the process for doing this here: https://docs.github.com/en/repositories/archiving-a-github-repository/referencing-and-citing-content

As you address these items, please take this last chance to review your reference list to ensure that it is complete and correct. If you have cited papers that have been retracted, please include the rationale for doing so in the manuscript text, or remove these references and replace them with relevant current references. Any changes to the reference list should be mentioned in the cover letter that accompanies your revised manuscript.

In addition to these revisions, you may need to complete some formatting changes, which you will receive in a follow up email. A member of our team will be in touch with a set of requests shortly. If you do not receive a separate email within a few days, please assume that checks have been completed, and no additional changes are required.

We expect to receive your revised manuscript within two weeks.

To submit your revision, please go to https://www.editorialmanager.com/pbiology/ and log in as an Author. Click the link labelled 'Submissions Needing Revision' to find your submission record. Your revised submission must include the following:

- a cover letter that should detail your responses to any editorial requests, if applicable, and whether changes have been made to the reference list

- a Response to Reviewers file that provides a detailed response to the reviewers' comments (if applicable, if not applicable please do not delete your existing 'Response to Reviewers' file.)

- a track-changes file indicating any changes that you have made to the manuscript.

NOTE: If Supporting Information files are included with your article, note that these are not copyedited and will be published as they are submitted. Please ensure that these files are legible and of high quality (at least 300 dpi) in an easily accessible file format. For this reason, please be aware that any references listed in an SI file will not be indexed. For more information, see our Supporting Information guidelines:

https://journals.plos.org/plosbiology/s/supporting-information

*Published Peer Review History*

Please note that you may have the opportunity to make the peer review history publicly available. The record will include editor decision letters (with reviews) and your responses to reviewer comments. If eligible, we will contact you to opt in or out. Please see here for more details:

https://plos.org/published-peer-review-history/

*Press*

Should you, your institution's press office or the journal office choose to press release your paper, please ensure you have opted out of Early Article Posting on the submission form. We ask that you notify us as soon as possible if you or your institution is planning to press release the article.

*Protocols deposition*

To enhance the reproducibility of your results, we recommend that if applicable you deposit your laboratory protocols in protocols.io, where a protocol can be assigned its own identifier (DOI) such that it can be cited independently in the future. Additionally, PLOS ONE offers an option for publishing peer-reviewed Lab Protocol articles, which describe protocols hosted on protocols.io. Read more information on sharing protocols at https://plos.org/protocols?utm_medium=editorial-email&utm_source=authorletters&utm_campaign=protocols

Please do not hesitate to contact me should you have any questions.

Sincerely,

Christian

Christian Schnell, PhD

Senior Editor

cschnell@plos.org

PLOS Biology

------------------------------------------------------------------------

Reviewer remarks:

Reviewer #1 (Michael C Schmid): Thank you for addressing all my previous comments.

Reviewer #2: I scrutinized the revised manuscript, and the authors have performed new analyses to answer most of the major comments that we had. Although, in some of the new analyses they did not get a statistically significant difference, I think the new analyses still answer the questions we had. Additionally, regarding the comments on laminar analysis, they performed new analyses with respect to the depth of the electrode. Although it does not explicitly answer questions about layers, but again I think they had an honest attempt to answer the reviewer question and perfectly acceptable from my end. I would accept this manuscript with no further revisions required.

Valentin Dragoi and Sunny Nigam

Reviewer #3: The central logic of the paper is still a head-scratcher to me. Let me try again, just for my sanity if nothing else.

The rebuttal states that the question being addressed is "whether decision signals that appear locally in visual cortex are causally linked to behavior." The word 'signals' in this sentence must refer to LFPs, because the object of study here is the correlation between LFPs and behavior. Yet they go on to agree that there is "no obvious way" that LFPs can be causal, and that instead, the parsing into high/low gamma is a way of isolating "different anatomical sources of presynaptic input." This is the exact point I made previously: the causality of LFPs must be attributed to their presynaptic source.

But now we have a quandary. If LFPs cannot affect local spiking, how could it be that abolishing local spiking removes the correlation between (high-gamma) LFP and behavior? It can only be that the causality goes in the opposite direction: local spiking drives high-gamma into patterns that correlate with behavior, because those spikes themselves are causal — as we already knew from previous work and the current Figure 1. So, again, what have we learned exactly?

(I also don't see how the phrasing of the "specific alternative to Britten's hypothesis, namely that the signals that are causally linked to behavior are echoed in visual cortex" does justice to what (at least the authors claim) is new here. It is well established from prior work that CPs reflect 'top-down' signals rather than (or in addition to) the feedforward causal effect of the neurons. But I digress.)

Nevertheless, being in the minority I would have been inclined to acquiesce, even without considering the cool new analyses and many improvements to the manuscript in response to the other reviewers. With considering them, the study is clearly above threshold and I happily endorse publication. Let's see if anyone else has the same reaction I did!

# Author comment: Author response to Decision Letter 3

**DOI:** 10.1371/journal.pbio.3003873.r005

7 Jun 2026

Attachment Submitted filename: PBIO_response_to_reviewers_FINAL_auresp_3.docx

# Editor report: Decision Letter 3

**DOI:** 10.1371/journal.pbio.3003873.r006

**Authors:** Christian Schnell, PhD

9 Jun 2026

Dear Dr Hou,

Thank you for the submission of your revised Short Reports "Distinct sources of decision-related signals in visual cortex are represented in different local field potential bands" for publication in PLOS Biology. On behalf of my colleagues and the Academic Editor, Simon Hanslmayr, I am pleased to say that we can in principle accept your manuscript for publication, provided you address any remaining formatting and reporting issues. These will be detailed in an email you should receive within 2-3 business days from our colleagues in the journal operations team; no action is required from you until then. Please note that we will not be able to formally accept your manuscript and schedule it for publication until you have completed any requested changes.

While you attend to these requests, please also ensure that you provide a link in the corresponding figure legends to the source data, for example: The underlying numerical data are provided at https://doi.org/10.5281/zenodo.20583873.

Please take a minute to log into Editorial Manager at http://www.editorialmanager.com/pbiology/, click the "Update My Information" link at the top of the page, and update your user information to ensure an efficient production process.

PRESS

We frequently collaborate with press offices. If your institution or institutions have a press office, please notify them about your upcoming paper at this point, to enable them to help maximize its impact. If the press office is planning to promote your findings, we would be grateful if they could coordinate with biologypress@plos.org. If you have previously opted in to the early version process, we ask that you notify us immediately of any press plans so that we may opt out on your behalf.

We also ask that you take this opportunity to read our Embargo Policy regarding the discussion, promotion and media coverage of work that is yet to be published by PLOS. As your manuscript is not yet published, it is bound by the conditions of our Embargo Policy. Please be aware that this policy is in place both to ensure that any press coverage of your article is fully substantiated and to provide a direct link between such coverage and the published work. For full details of our Embargo Policy, please visit http://www.plos.org/about/media-inquiries/embargo-policy/.

Thank you again for choosing PLOS Biology for publication and supporting Open Access publishing. We look forward to publishing your study.

Sincerely,

Christian

Christian Schnell, PhD

Senior Editor

PLOS Biology

cschnell@plos.org
